# Purchases of Fruit and Vegetables for at Home Consumption During COVID-19 in the UK: Trends and Determinants

**DOI:** 10.3389/fnut.2022.847996

**Published:** 2022-04-01

**Authors:** Cesar Revoredo-Giha, Carlo Russo, Edward Kyei Twum

**Affiliations:** ^1^Department of Rural Economy, Environment and Society, Scotland's Rural College, Edinburgh, United Kingdom; ^2^Department of Economics and Law, University of Cassino and Lazio Meridionale, Cassino, Italy

**Keywords:** UK fruit and vegetable consumption, COVID-19, online shopping, panel data analysis, impact response framework

## Abstract

This paper addresses the issue of fruit and vegetable purchases in the UK during the COVID-19 pandemic. The study is motivated by the importance of fruit and vegetables for human nutrition, health and reduction of population obesity, especially in the UK where per capita consumption is still below recommended levels. A rich panel dataset was used reporting actual shopping places and quarterly expenditure for at-home consumption of fruit and vegetable purchases of 12,492 households in years 2019 and 2020. The unique dataset allowed us to compare expenditure for fruit and vegetables before and after the COVID-19 outbreak and to identify the main drivers of changes in purchases. Regression analysis found that expenditure increased ~3% less than what expected given the overall increase in the numbers of at-home meals during lockdown. Also, Online shopping was found to be an alternative source for fruit and vegetables purchase during the pandemic. However, the expenditure for processed products grew more than the one for fresh products, resulting in a reduction of the relative share of the latter and possible deterioration of the diet quality.

## Introduction

Fruit and vegetable consumption are an important part of human nutrition and a key component of the UK strategy to reduce obesity. Despite this, the UK per capita consumption of fruit and vegetables is below the recommended levels (1). The lockdown that followed the COVID-19 epidemic in March 2020 brought a number of constraints to households such as restrictions regarding access to shopping locations and allocation of time for shopping and cooking. These constraints as well as other factors coming from the market environment may have changed households' demand for fruit and vegetables.

In the described context, the purpose of this paper is to investigate how COVID-19 affected the purchases of fruit and vegetables in the UK. The analysis addresses not only the overall purchases but also a possible substitution between fresh and processed or preserved products during the pandemic. The objective of the research is not only to measure any change in diet, but also to identify the social and economic drivers of the change. The analysis of the causes is important to assess how much of the diet change was due to the pandemic *per se* and how much was due to the containment measures that were adopted by the government and ultimately to stipulate on future trends.

### An Impact-Response Conceptual Framework

The effect of COVID-19 on fruit and vegetable purchases can be represented by an Impact-Response (IR) framework (2). In this model, changes in the variable of interest (purchases of fruit and vegetables) are determined by the response of a social group (UK consumers) to the impact (the social and economic consequences) of an exogenous event (the pandemic outbreak). The framework has been used in several COVID-19 related work [e.g., (3–8)]. The IR framework helps to understand interaction among variables and the outcome of the interaction. In this case, how the purchases of fruit and vegetables, UK consumers, and the consequences of the COVID-19 outbreak interact resulting in possible changes in fruit and vegetables purchases can be better explained. In particular, IR framework contributes to the understanding of the effects of COVID-19 on nutrition because it allows researchers to investigate the causes of a possible change in diet during the pandemic. To this end, this paper not only assess the difference in fruit and vegetables purchases during the period of interest, but it identifies the main factors driving the change.

**Figure 1:**
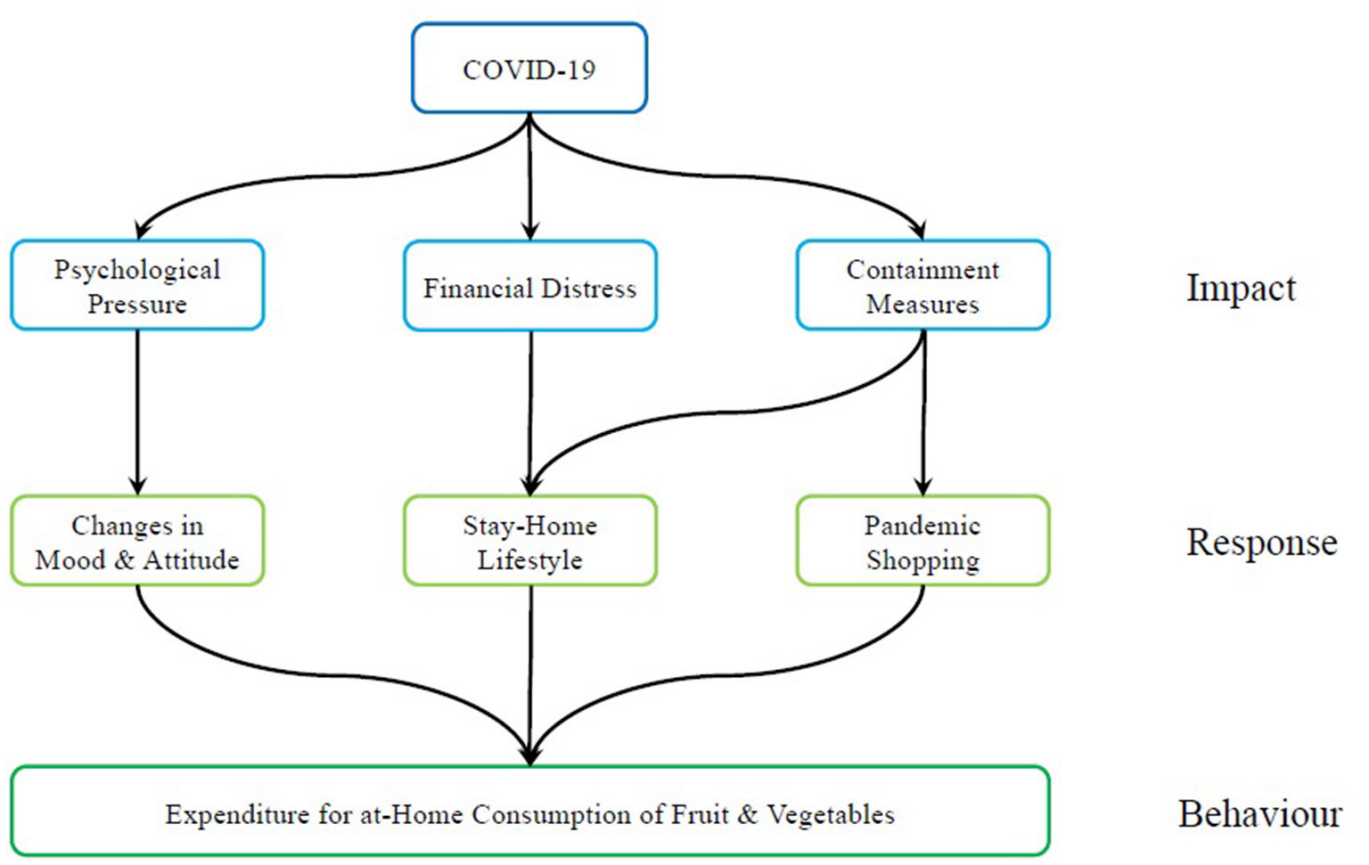
Impact-response framework.

Figure 1 summarizes the application of the IR framework. Based on a review of the literature we identified three main areas of impact of COVID-19: Psychological pressure, Financial distress and Containment measures. Consumers responded to these impacts in several ways: changing their mood, lifestyle and shopping habits and ultimately changing the purchases of fruit and vegetables.

### Impact Areas of COVID-19

Psychological pressure is defined as the effect of the COVID-19 outbreak on the psychological wellbeing of UK populace [e.g., (9)]. In general, the pandemic emergency was associated with psychological issues such as stress, fear, anxiety, depression and frustration [e.g., (10, 11)]. An extensive literature suggested that COVID-19 Psychological pressure may influence food-purchasing behavior [e.g., (12)].

Financial distress is the second area of impact. COVID-19 affected the UK economy in multiple ways, including increasing business uncertainty (13), disruption of supply chains (14, 15) and ultimately higher unemployment rate (16). Despite large public subsidies, the economic consequences of the pandemic have been dire for many UK households (17). The resulting financial distress is expected to affect the purchases of fruit and vegetables depending on income elasticity of demand.

Finally, the spreading of the disease required adopting drastic containment measures, including lockdown, mobility limitations, school and non-essential business closure, and voluntary social distancing. This area of impact was particularly severe during the 1st months of the emergency, when government restrictions were in place and became less compelling at later stages of the pandemic (18).

### Expected Responses of UK Consumers

The three areas of impact elicited responses from UK consumers. Based on our review of the literature, three main types were identified: Changes in mood and attitude, At-home lifestyle, and Pandemic shopping (Table 1).

**Table 1:** Consumer response to COVID-19, their expected effects on expenditure for fruit and vegetables and on the relative preference for fresh products over preserved/processed ones.

**Consumer responses**		**Expected effects**	
		**On expenditure for fruit and vegetables**	**On preference for fresh over preserved fruit and vegetables**
Changes in mood and attitude	Panic & hoarding	Increase	Decrease of fresh
	Health focus	Increase	Increase of fresh
	Comfort-seeking	Decrease	-
At-home lifestyle	Eating at home	Increase	-
	Adjusting budget	Decrease	Decrease of fresh
Pandemic shopping	Online shopping	-	-
	Fewer trips to store	-	Decrease of fresh
	Store choice	-	-

*Source: Own elaboration based on the literature review*.

#### Changes in Mood and Attitude

Response to Psychological pressure triggered changes in consumer mood and attitude, resulting in new purchasing behavior. In the early stages of the pandemic, even before the national emergency was declared, UK consumers displayed panic buying, and stockpiling behavior [e.g., (19, 20), which may be interpreted as a precautionary response to the fear of future scarcity and restrictive measures on mobility. Other studies showed that COVID-19 induced emotional status such as anxiety, depression or stress were associated with changes in eating habits such as emotional eating or binging (21, 22).

Concerns about future food availability, leading to hoarding and stockpiling behavior (19, 23), may result in a relative preference toward non-perishables processed fruit and vegetables over fresh ones. O'Connell et al. (24) analyzed a balanced panel data of 17,093 UK households from January 1, 2019, to August 9, 2020, and found that, there was a spike in household purchases prior to the first nationwide lockdown in March 23, 2020. The authors observed sharp increase in staples (including canned products) purchases relative to perishable products (including fresh fruit and vegetables) which showed a moderate increase only. Richards and Rickard (25) analyzed the Canadian fruit and vegetable market and indicated that consumers stockpiled frozen fruit and vegetables. The authors also predicted a switch toward online shopping after observing closure of food services. In the United States, Litton and Beavers (26) analyzed a recall dataset of Michigan State residents (survey was conducted from June 17 to June 29) and found that food-insecure residents were more likely to consume less fruits and vegetables (being it fresh, frozen, or canned) during the COVID-19 pandemic.

Psychological pressure can affect dietary choices in other ways as well. On the one hand, health concerns may result in a relative preference toward fresh fruit and vegetables due to a focus on healthy nutritional balance, hoping to boost human immune system and possibly the resistance to contagion (27–29). On the other hand, anxiety and fear may lead to an increase in purchases of comfort food such as snacks, confectionery, sweets, alcohol (30) to the possible detriment of fruit and vegetables.

Based on the literature, it is possible to conclude that changes in consumer mood and attitude due to psychological pressure may drive purchases of fruit and vegetables into different directions and the net effect on purchases depends on which component prevails.

#### Stay-Home Lifestyle

Response to the COVID-19 impact include adopting a stay-home lifestyle. Due to fear of contagion, restrictive measures, at-home working or involuntary unemployment, UK consumers spent more time at home than they did before the pandemic. The obvious consequence was a sharp decrease in the number of times they ate out and an increase in the budget expenditure for grocery product (including fruit and vegetables). Studies about the effect of stay-home lifestyle on dietary habits found conflicting results (31). On the one hand, it was associated with healthy eating due to home cooking [e.g., (32)]. On the other hand, confinement was found to lead to increase in consumption of comfort food, less exercise and more time spent on watching TV (33). The net effect of the two trends is an empirical question. Furthermore, it must be noted that changes in employment status may affect consumer response deeply.

#### Pandemic Shopping

Finally, UK consumers responded to the impact of COVID-19 by adjusting their shopping behavior, that is, the way they purchased food. Online purchases increased during the pandemic both from de-specialized retailers (such as Amazon) and specialized food retailers developing online services along their traditional “brick and mortar” stores (such as Tesco online) [e.g., (34, 35)]. Consumers optimized shopping frequency and store choice given the new sets of constraints to mobility and accounting for the possibility of contagion [e.g., (36)]. The emerging shopping behavior is expected to affect purchases of fruit and vegetables in two main ways. Firstly, changing food source (for examples, from far, large supermarket to local stores or to online); consumers are exposed to different assortments, and this may result in an adjustment in purchases. Secondly, the objective of reducing shopping frequency may lead to a preference for non-perishable goods in order to avoid waste and extend the time before a new trip to the store is needed.

### Change in Fruit and Vegetables Purchases

The final objective of the study is to break down the overall change in fruit and vegetables purchases, measuring the effects of each response. The final outcome is a quantitative evaluation comparing the relative magnitude of each factor.

## Materials and Methods

In order to achieve these objectives, a two-step statistical approach was developed. First, the variables describing changes in expenditure for at home consumption of fruit and vegetables and the three consumer responses to COVID-19 impacts were identified and measured. Second, an econometric model was used to estimate the contribution (relative effect) of each response to the change in diet.

The econometric model was necessary to investigate the causal relationship that is embedded in the IR framework. According to econometric theory, the regression coefficients measure the expected change in the expenditure for at-home consumption of fruit and vegetables due to a change in a response variable, keeping all other explanatory variables constant [e.g., (37)]. In this way, it is possible to single out and compare the contribution of each response.

### Identification and Measurement of Variables

#### The Dataset

In order to explain the changes in expenditure, a subset of Kantar Worldpanel Homescan panel dataset was used. This recurring survey collects data about grocery purchases of a representative sample of UK households. Data are collected and certified by Kantar.

The use of this extensive survey allows us to provide a general estimate of the UK trends. However, because this is a multipurpose survey with predetermined questionnaire, it was impossible to collect *ad-hoc* information. Instead, it was necessary to use existing variables to measure the phenomena of interest. Therefore, the generality of the large sample was achieved at the cost of approximation of measurement.

The available dataset reported information about 12,492 UK households. For each individual household, data were reported about expenditure for fruit and vegetables and shopping places starting from January 1^st^, 2019 to December 31^st^, 2020. The information was aggregated by quarters of 13 weeks, which means that every household was observed 8 quarters in the dataset. In total, the dataset was composed of 99,936 observations (12,492 households in 8 quarters).

The use of quarterly data raises the issue of a proper identification and development of the pandemic period. In fact, the statistical analysis is based on the comparison of expenditure for at home consumption for fruit and vegetables before and after the COVID-19 outbreak. In the UK, the early cases were reported during February 2020, and the containment measure were adopted in March. Therefore, data of the first quarter 2020 report purchases before and after the disease outbreak. Because a precise measurement was not possible with the available data, it was assumed that the first quarter 2020 is part of the “before COVID-19” period. This choice was made because the disease became epidemic in the UK at the end of the quarter, and the majority of purchases in that period happened before the outbreak. Thus, the dataset is conventionally broken into two periods: from January 1^st^ 2019 to March 31^st^ 2020 is the “before COVID-19” period and from April 1^st^ to December 31^st^ 2020 is the “after COVID-19” period.

The panel was geographically balanced, and it covered seven regions: East with 1,349 households (10,792 observations), London with 913 households (7,304 observations), Midlands with 2,119 households (16,952 observations), North with 3,405 households (27,240 observations), South with 2,970 households (23,760 observations), Scotland with 1,104 households (8,832 observations) and Wales with 632 households (5,056 observations).

It was possible to use only a limited subset of the variables in the Kantar Worldpanel for this research. Available variables are described in Table 2. They included information regarding the purchases of fruit and vegetables, total grocery purchases, shopping places and individual household characteristics, such as age, sex and number of adults and children.

**Table 2:** Dataset description and measurement of consumer responses to COVID-19.

**Description**	**Variables**	
Per capita expenditure for fresh fruit &vegetables	PCEX_FFV	Dependent variables of the regression models
Per capita expenditure for processed fruit &veg.	PCEX_PFV	
Expenditure for Fresh Fr. &Veg/Total Fr. &Veg. Exp.	SHARE_F	
Per capita expenditure for all grocery products	PCEX_TOT	Meas. Lifestyle R.
Convenience store share of total exp. for all grocery	CONV	Measures of the pandemic shopping response
Discount store share of total exp. for all grocery	DISC	
Large store share of total exp. for all grocery	LARGE	
Club, Barg. & Other Store Share of To. Exp. for All Gr.	OTHER	
Online Share of Total Exp. for All Grocery.	ONLINE	
Seasonal binary variable (1 if 2^nd^ quarter ‘19, 0 otherw.)	Q2_19_	Measures of mood and attitude response
Seasonal binary variable (1 if 3^nd^ quarter ‘19, 0 otherw.)	Q3_19_	
Seasonal binary variable (1 if 4^nd^ quarter ‘19, 0 otherw.)	Q4_19_	
Seasonal binary variable (1 if 2^nd^ quarter ‘20, 0 otherw.)	Q2_20_	
Seasonal binary variable (1 if 3^nd^ quarter ‘20, 0 otherw.)	Q3_20_	
Seasonal binary variable (1 if 4^nd^ quarter ‘20, 0 otherw.)	Q4_20_	
Age of primary shopper	AGE	Auxiliary household information
Sex of primary shopper (1 if male, 0 otherw.)	SEX	
Number of children in the household	NCH	
Number of adults in the household	NAD	

The dataset reported the per capita expenditure for fruit and vegetables as well. In the original dataset, fruit and vegetables were classified in the following 8 categories: fresh and processed potatoes (e.g., fresh new potatoes and mashed potatoes); fresh green vegetables (e.g., lettuce), other fresh vegetables (e.g., carrots) and processed vegetables (e.g., sweet pickles) and fresh fruits (e.g., apples), processed fruits (e.g., fruit salad) and fruit juices (e.g., apple juice). In order to focus on the substitution between fresh and non-fresh fruit and vegetable products, the 8 categories have been summarized into two groups: Fresh Fruit and Vegetables (including fresh potatoes, fresh green vegetables, other fresh vegetables and fresh fruit) and Processed Fruit and Vegetables (all other categories).

The information regarding shopping places included the expenditure for grocery products at different outlets. The shops were classified in 6 groups namely: club and bargain store (e.g., Costco), convenience (e.g., Holland and Barrett), discounter (e.g., Lidl), large store (e.g., Tesco), online (i.e., any purchase done via the internet such as Tesco online) and other retailers (e.g., farmshop/stall). The dataset reported the per capita expenditure for grocery by store type for each household in each quarter.

#### Using Dataset Variables to Measure Consumer Responses

The Stay-Home Lifestyle response was measured by per capita expenditure for all grocery products. The variable can summarize the two main drivers of the response: on the one hand, the increase in the number of meals that are consumed at home (leading to an increase in expenditure), and on the other hand, the possible income loss due to impact of the pandemic on the economy (resulting in a decrease in expenditure depending on income elasticity).

The Pandemic Shopping response was measured through the shares of total grocery expenditure of each store type, including online. In this way the model can account for changes in the choice of shopping places due to restrictions to consumer mobility.

Finally, the Mood response was elusive to capture with the available data and it was measured as a residual effect. By assumption, any systematic change in expenditure for fruit and vegetables after the pandemic outbreak that could not be explained by Stay-Home Lifestyle or Pandemic Shopping is attributed to this response. In order to purge the estimation from other factors as much as possible, individual characteristics of the households have been considered in the regression model. In this way, the estimation of the Mood and Attitude response is not affected by changes in fruit and vegetables purchases due to the individual factors. In the econometric model, the residual effect is computed using three binary variables identifying the second, third and fourth quarters of 2020 (Table 2).

### Econometric Model

In order to estimate effects of the three types or response on fruit and vegetable expenditure, a random effect regression model was used. Appropriate statistical tests on the regression coefficients can be used to prove the existence and measure the magnitude of each response separately.

Equation (1) describes the functional form of the model:


(1) $$y_{i,t}=\beta_{0}+\sum_{k=1}^{K}\left(\beta_{k}+\gamma_{k}a_{t}\right)X_{i,t}^{k}+\sum_{j=1}^{J}\beta_{j}Z_{i,t}^{j}+u_{i}+e_{i,t}$$

where subscripts *i* and *t* refer to the *i*^*th*^ household in quarter *t* (when possible, the subscripts will be dropped for the sake of simple notation), and

- *y*_*i, t*_ is the dependent variable.
- *a*_*t*_ is a binary variable that is equal to 1 if the observation refers to the “after COVID-19” period (the second, third or fourth quarter 2020) and zero otherwise.
- $X_{i,t}^{k}$ are the set of variables that are used to measure the Mood and Attitude, Stay-Home Lifestyle and Pandemic Shopping responses as listed in Table 2. Their effect on *y*_*i, t*_ is expected to change after the COVID-19 outbreak, because of impact of the three effects on consumer behavior.
- $Z_{i,t}^{j}$ are the set of *auxiliary household information* which effect on *y*_*i, t*_ is expected to be unchanged after the COVID-19 outbreak.
- β's and γ's are regression parameters.
- *u*_*i*_ and *e*_*i, t*_ are error terms.

According to the textbook dummy variable regression technique, an estimate of a γ_*k*_ parameter that is statistically different from zero allow us to reject the null hypothesis that the effect of *X*^*k*^ on *y* did not change after the COVID-19 outbreak. This property was used to test for the three effects. A change in the regression parameters of interest change after the COVID-19 outbreak can be considered as statistical evidence of the effect of the response to COVID-19 on expenditure for at home consumption of fruit and vegetables. The statistical tests are structured as follows:

A. The test for the effect of Lifestyle response on the dependent variable is based on the following hypotheses:
H_0_: γ_PCEX_TOT_ = 0 (no effect)
H_1_: γ_PCEX_TOT_ ≠ 0 (Lifestyle response affected expenditure)

where γ_PCEX_TOT_ is the γ coefficient of the variable PCEX_TOT. If the null hypothesis is rejected, it is concluded that Lifestyle response affect the expenditure for fruit and vegetables. A positive (negative) γ_PCEX_TOT_ implies that the dependent variable increased more (less) than expected given the increase in the total expenditure for grocery. This means that after COVID-19, having more meal at home does not result in an increase in expenditure for fruit and vegetables in the same proportion as before COVID-19.

B. The test for the effects of Pandemic Shopping response are:
H_0_: β_STR_+γ_STR_ = 0 ∀ STR = {CONV, DISC, LARGE,
OTHER, ONLINE}
H_1_: at least one β_STR_+γ_STR_≠0

where γ_STR_ and β_STR_ are the regression coefficients of the expenditure share of the corresponding store type. If the null hypothesis is rejected, a change in the shopping place affected the dependent variable in the after COVID-19 period. In this case, it is possible to conclude that mobility restrictions affected not only the type of stores but also the way consumer shopped during the pandemic and ultimately their expenditure for fruit and vegetables.

C. The test for the effect of Mood and Attitude response is:
H_0_: γ_Qi_ = 0 ∀ Q_i_ = {2020 quarter 2, 2020 quarter 3, 2020
quarter 4}
H_1_: at least one γ_Qi_ ≠ 0

If the null hypothesis is rejected, the regression intercepts after the COVID-19 differ from those before. This means that there is a systematic component that is not captured by the other variables, that can be attributed to this response.

In the next section, descriptive statistics and the results of the regression estimates are presented and discussed.

## Results

### Descriptive Statistics

In this section the data are described, with a focus on the measures of Lifestyle and Pandemic Shopping responses that have been introduced in the previous section. A descriptive analysis of the trends in expenditure for Fresh and Processed Fruit and Vegetables is presented as well.

#### Measuring Stay-Home Lifestyle Response

**Figure 2:**
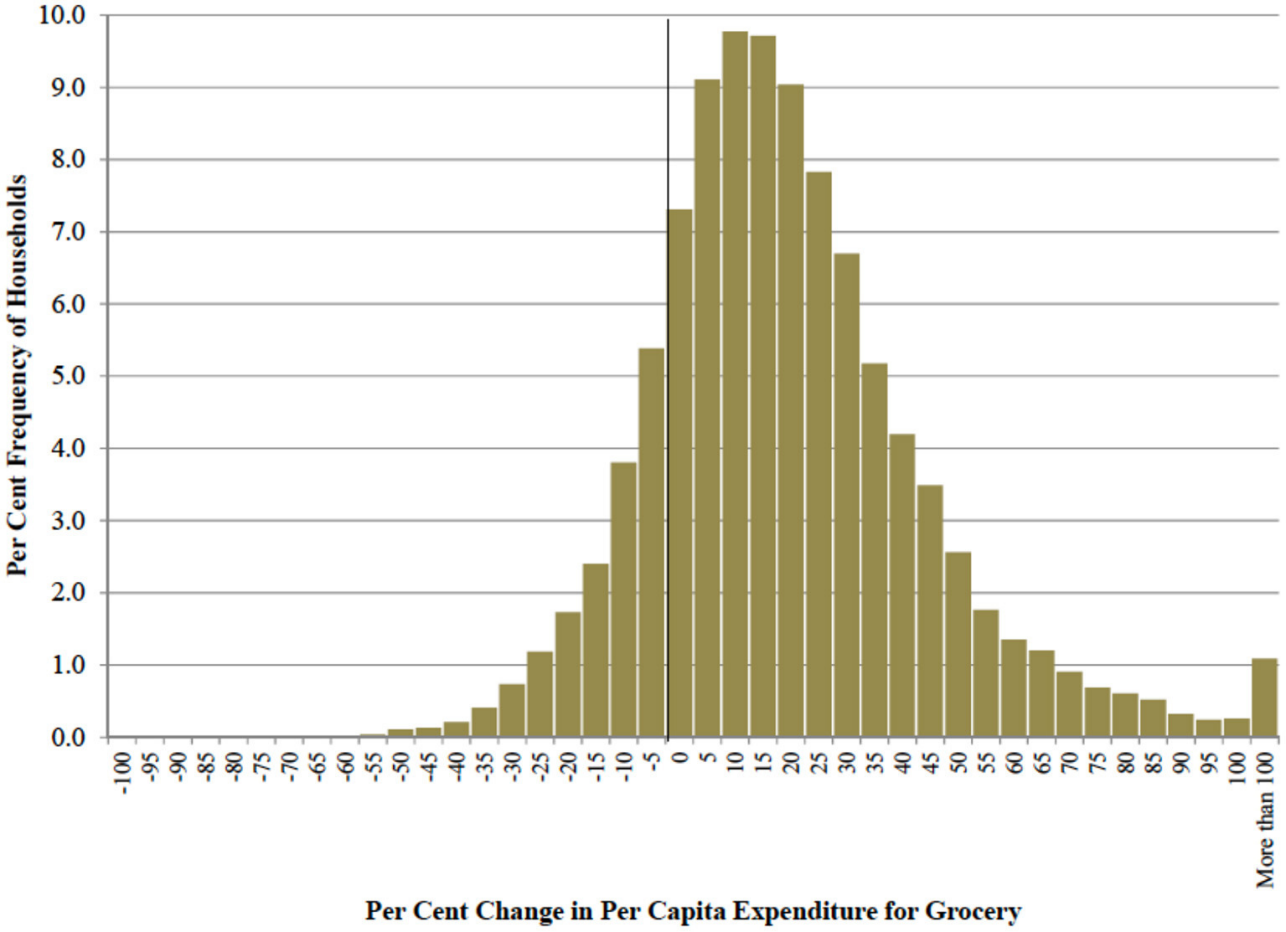
Distribution of UK households by class of per-cent change in per capita expenditure for grocery goods before and after COVID-19 lockdown (per cent frequencies).

Total expenditure for grocery was used as concise measure of changes in lifestyle. Figure 2 reports the distribution of the per cent change in the UK per capita expenditure for all grocery goods before and after the COVID-19 outbreak. The mean value of the distribution is 17.2% and the standard deviation is 26.6. The 95% confidence interval for mean is between [16.7, 17.6]. The median of the distribution is 14% and the first and the third quartile are 0.9 and 29.5%, respectively. The share of UK households experiencing a reduction in per capita grocery expenditure is 16.2%. Summarizing, majority of UK households increased their grocery expenditure between 10 and 30% after COVID-19 outbreak and approximately one household out of six reduced the expenditure.

The data confirm the expected increase in per capita grocery expenditure due to the stay-home lifestyle. However, the analysis of the distribution shows that a non-negligible number of households experienced a reduction in the budget for grocery, possibly because of the financial distress impact of COVID-19.

#### Measuring Pandemic Shopping Response

After the COVID-19 outbreak, consumer mobility was constrained by government measures and fear of contagion. Changes in shopping behavior are used to measure such effect.

**Figure 3:**
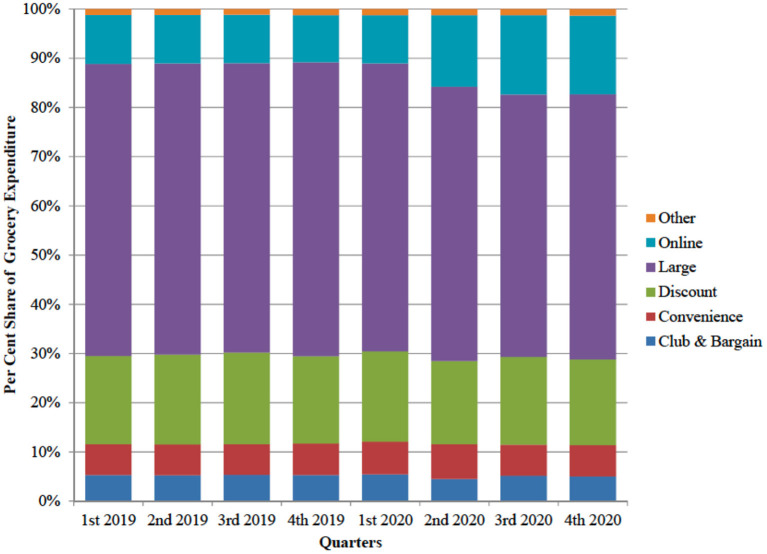
Expenditure for all grocery goods by types of store (UK, per cent share).

Figure 3 reports the per cent shares of expenditure for all grocery product by type of store. The per cent share of online purchases increased from an average of 9.8% before COVID-19 outbreak to an average of 15.6% after the outbreak. In the same period, the share of large stores decreased from 59.1% to 54.3%. The remaining store types exhibited minor changes in share.

**Figure 4:**
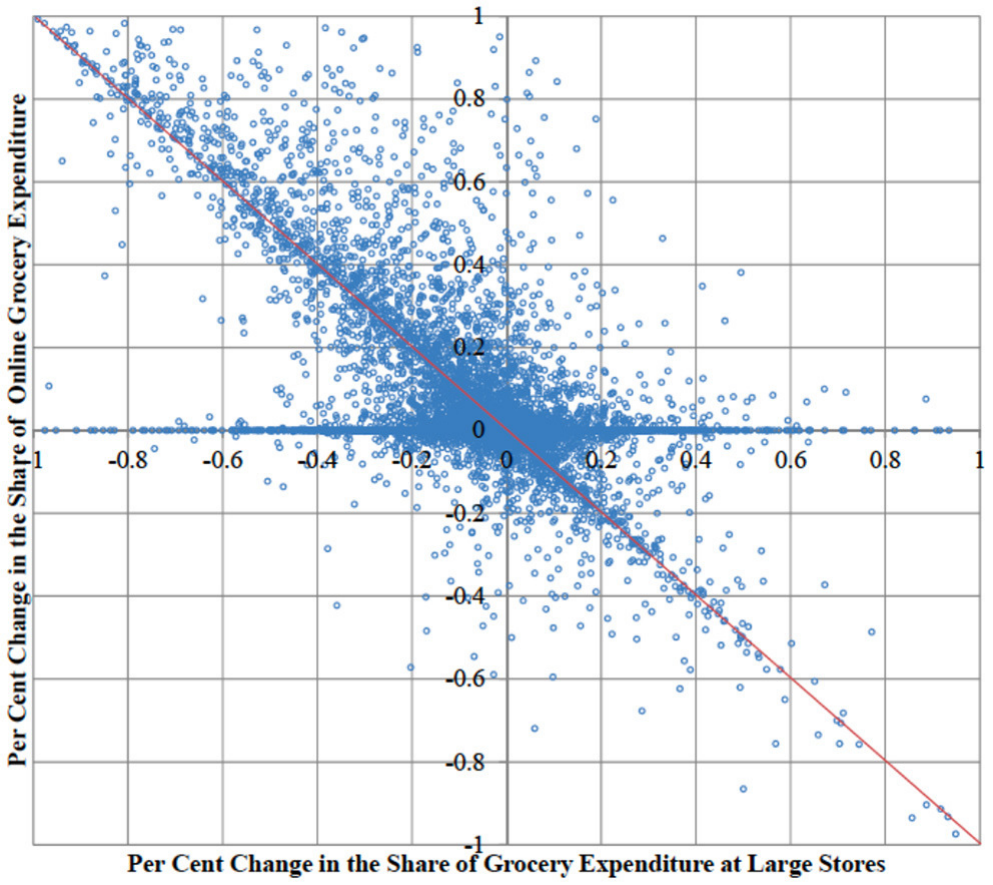
Plot of per cent changes in household grocery expenditure at large stores and online before and after Covid-19 outbreak (UK, data are individual households).

Although the aggregate data suggest an overall shift of consumer expenditure from large stores to online, individual behaviors exhibit heterogeneous patterns. Figure 4 reports a by-plot comparing the per cent change in the expenditure for all grocery goods at large stores and the one from online purchases. The majority of observations lie in the second cartesian quadrant of the plot, confirming the strong substitution effect between the two outlets. On the diagonal of the second quadrant, a reduction in the share of expenditure at large stores is matched by an increase of the share of online purchases exactly. However, the substitution is not perfect, because a remarkable number of observations is far from the perfect negative correlation line (the red line in the figure), suggesting that online shopping acted as substitute for other outlets as well.

#### Trends in Expenditure for Fruit and Vegetables

Using the dataset, the average per-capita expenditure for at-home consumption of fresh fruit and vegetables and processed fruit and vegetables was computed in each quarter of 2019 and 2020. The results were compared with the average per-capita expenditure for grocery goods in the same period, in order to account for seasonality in fruit and vegetables consumption. Table 3 reports the results.

**Table 3:** Average per-capita expenditure for at-home consumption of fresh fruit and vegetables, processed fruit and vegetables and grocery goods (UK 2019–20, pounds).

		**1^**st**^ Quarter**	**2^**nd**^ Quarter**	**3^**rd**^ Quarter**	**4^**th**^ Quarter**
Fresh fruit & vegetables	2019	38.0	39.8	38.2	33.4
	2020	38.0	46.7	41.3	36.5
	Diff.	0.0	6.8^(*)^	3.0^(*)^	3.1^(*)^
	% Diff.	0.0	17.1	7.9	9.2
Processed fruit & vegetables	2019	26.1	26.0	25.1	26.8
	2020	27.6	30.9	28.4	30.0
	Diff.	1.5^(*)^	4.8^(*)^	3.3^(*)^	3.1^(*)^
	% Diff.	5.8	18.5	13.3	11.6
All grocery goods	2019	356.3	361.5	351.3	387.8
	2020	375.0	427.0	400.3	429.3
	Diff.	18.6^(*)^	65.5^(*)^	49.0^(*)^	41.5^(*)^
	% Diff.	5.2	18.1	14.0	10.7

(*)Difference in the average expenditure between 2019 and 2020 is statistically significant at 95% confidence level.

*The statistical test used is the t-test of equality of sample means*.

The data in Table 3 show a statistically significant increase in the expenditure for at home consumption of both categories of fruit and vegetables during the COVID-19 period. However, the per-capita expenditure for fresh fruit and vegetables in the fourth quarter of 2020 grew less than the per-capita expenditure for all grocery goods, suggesting a change in the composition of the consumption basket.

**Figure 5:**
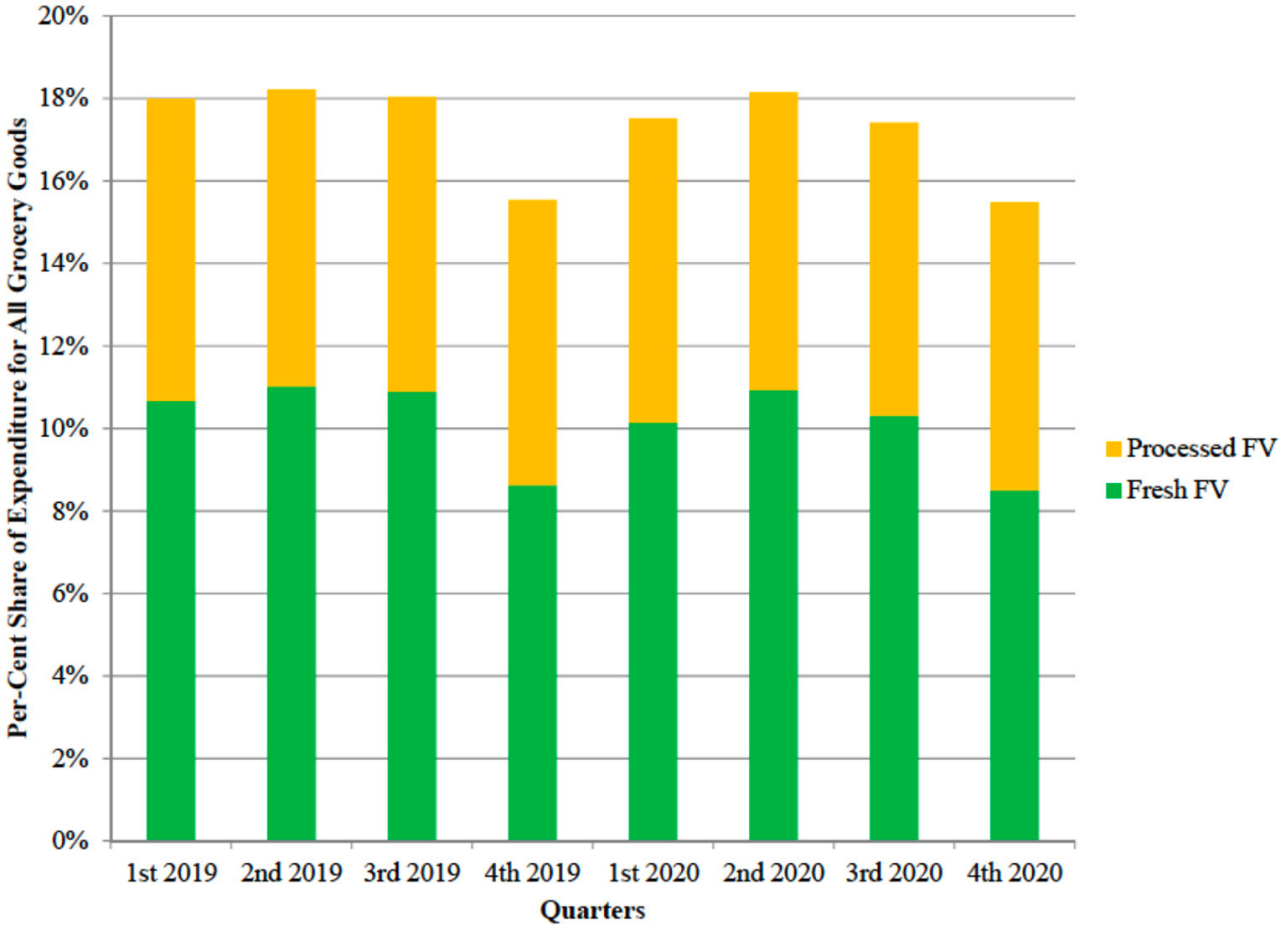
Per-cent share of per-capita expenditure for all grocery goods of fresh fruit and vegetables and processed fruit and vegetables (UK, years 2019–2020).

Figure 5 compares the percentage share of fresh fruit and vegetables and processed fruit and vegetables per-capita expenditure on the per-capita expenditure for all grocery goods. The graph shows minor differences between shares in 2019 and 2020 once seasonality has been accounted for. Only in the first and third quarter of 2020, the expenditure for fruit and vegetables was a lower share of grocery expenditure than in 2019, but even in those quarters, the difference was limited (about 0.5% points). The data suggests that COVID-19 had a limited effect on consumers' decisions on the allocation of the overall budget for grocery to fruit and vegetables purchases.

**Figure 6:**
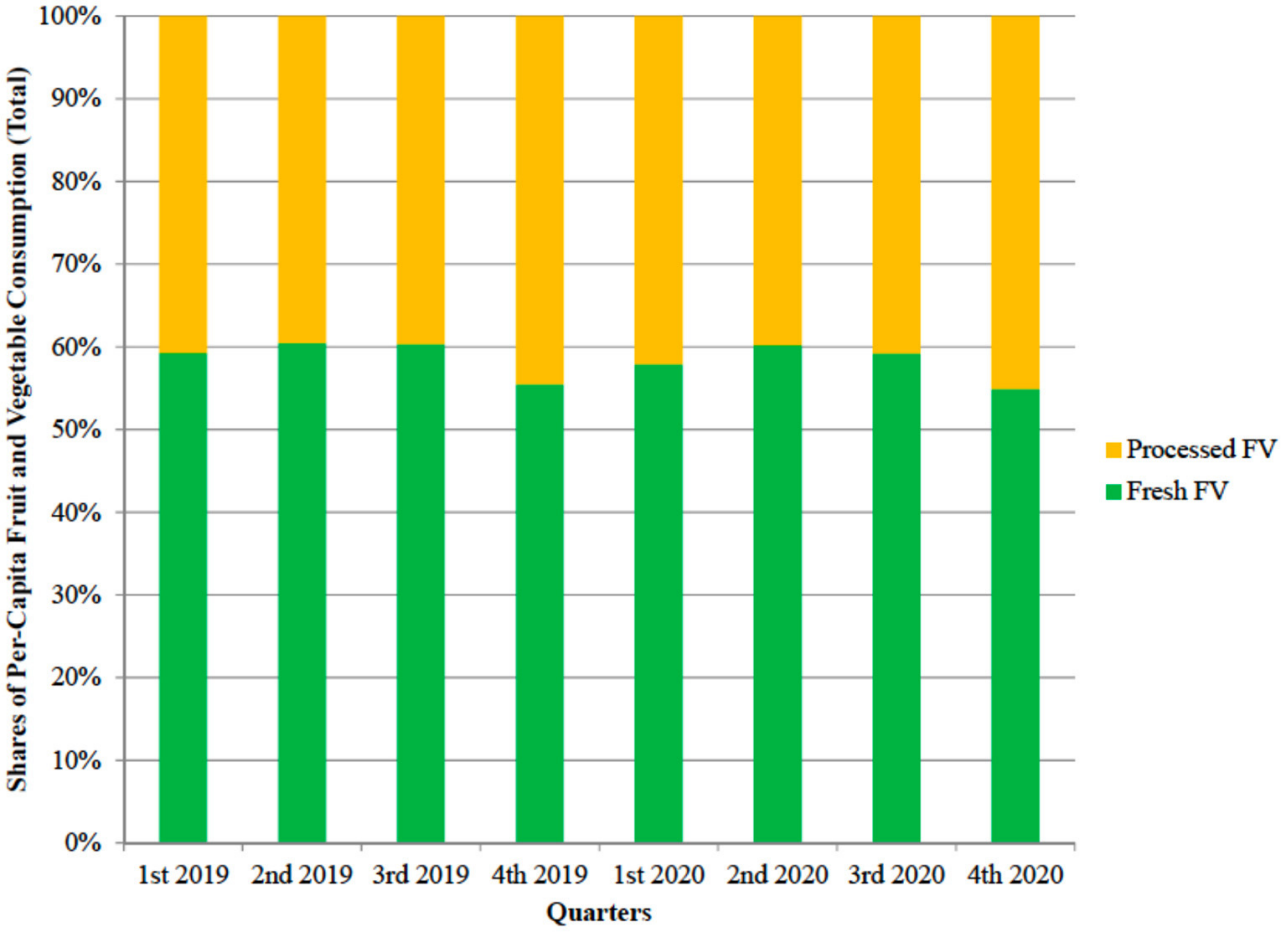
Break down of per-capita expenditure for fruit and vegetables (total) into expenditure shares for fresh fruit and vegetables and processed fruit and vegetables (per-cent shares, UK, years 2019–2020).

Similarly, the expenditure shift from fresh to processed fruit and vegetables was limited. Figure 6 reports the per-cent shares of the two categories on total fruit and vegetable expenditure. Again, after accounting for seasonality, only minor differences are observed.

**Figure 7:**
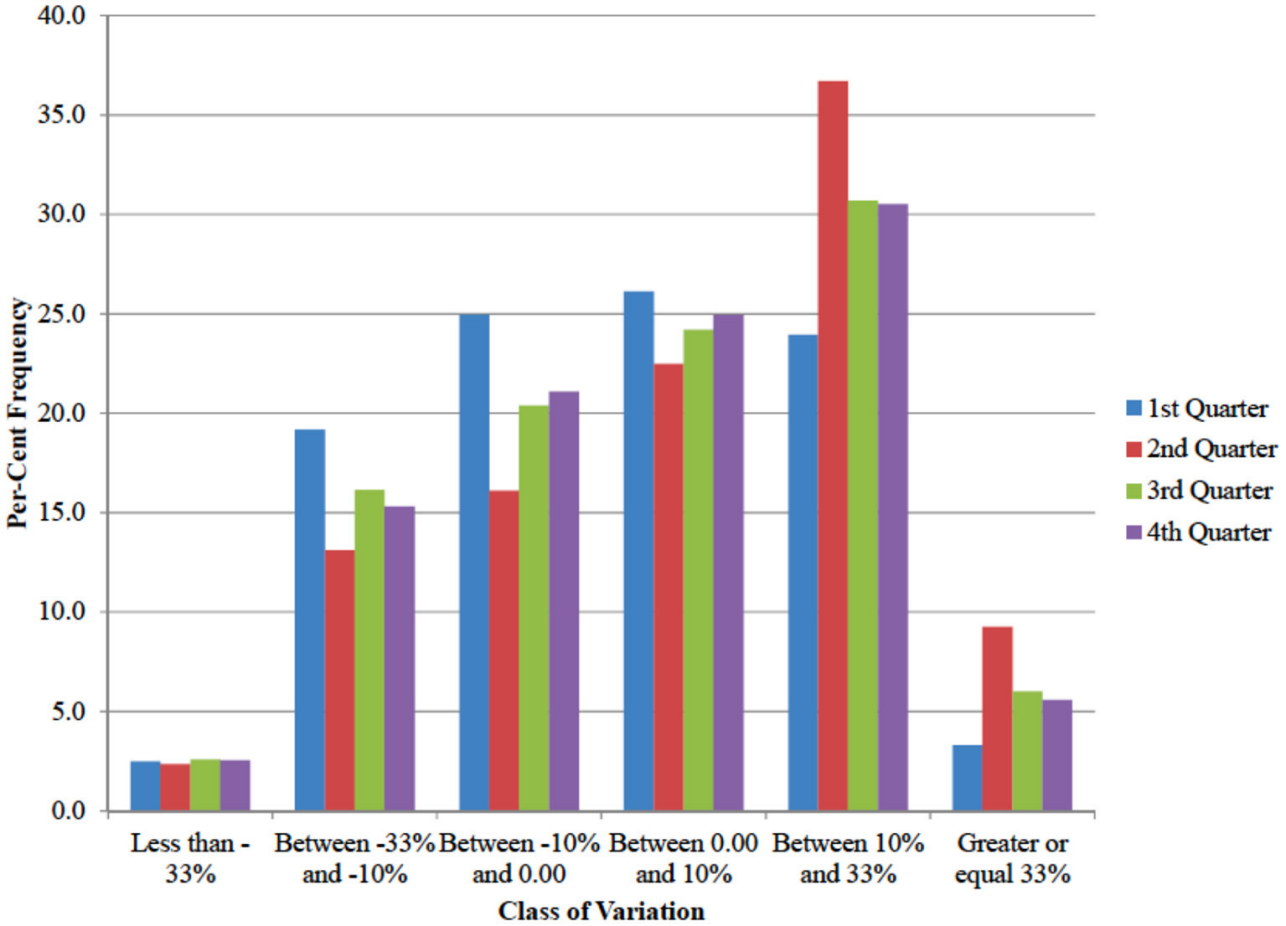
Distribution of households by class of per-cent change in household expenditure for fruit and vegetables (per-cent frequencies, quarters 2020 compared to same quarter in 2019).

In order to account for the heterogeneity in consumer responses to COVID-19 further, the household distribution of the expenditure for fruit and vegetables was investigated. Figure 7 shows that on average 36.6% of consumers experienced a decrease in expenditure for fruit and vegetables after COVID-19, compared to the same quarter in the previous year. The figure was 46.6% in the before COVID-19 winter quarter. At the same time, 39.6% of the consumers in the sample increased their expenditure by more than 10% with respect to the same quarter in 2019. In the period before COVID-19 winter quarter they were 27.2%.

**Figure 8:**
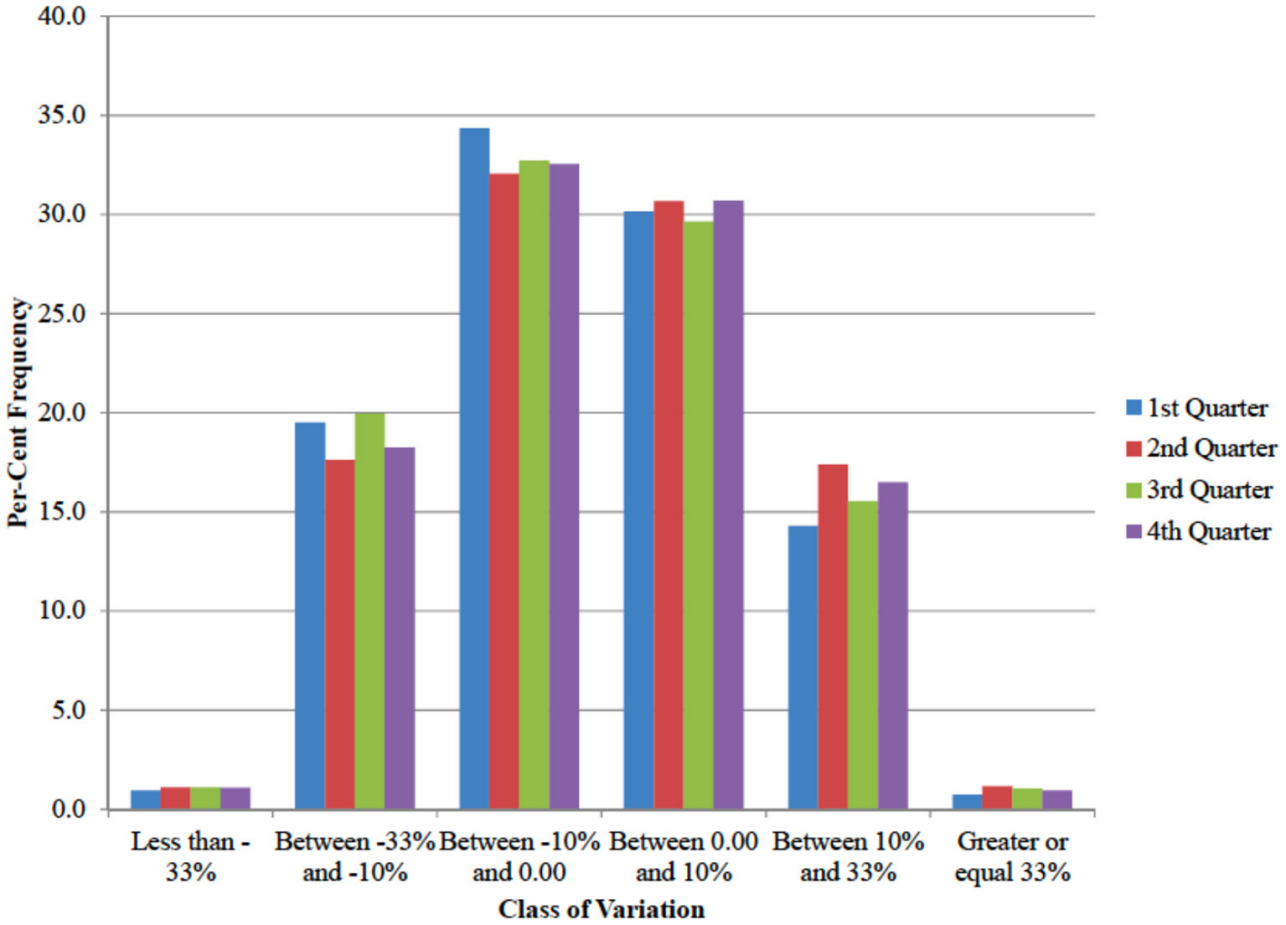
Distribution of households by class of change in expenditure share of fresh fruit and vegetable over all fruit and vegetables. (percentage frequencies, quarters 2020 compared to same quarter in 2019).

The analysis of quarterly data (Table 3) showed a spike in expenditure for at home consumption of fruit and vegetables during the second quarter 2020, that is right after the COVID-19 outbreak. Similarly, the unchanging mean in expenditure share for fresh fruit and vegetables is the result of a symmetric distribution where roughly half of the consumers increased the relative expenditure for fresh produce and the other half reduced it (Figure 8). To account for data heterogeneity, a regression model on individual household data was run.

### Regression Results

Table 4 reports the description and summary statistics of the variables used in the regression model. Data report an increase in the expenditure for both Fresh and Processed Fruit and Vegetables in the after COVID-19 period. The share of Fresh Fruit and Vegetables expenditure did not change after the pandemic outbreak.

**Table 4:** Descriptive statistics.

			**Bef. Covid-19**		**After Covid-19**	
**Description**	**Variables**	**Units**	**Mean**	**St. Dev**	**Mean**	**St. Dev**
Per Capita Expenditure for Fresh F&V	PCEX_FFV	£	37.51	32.74	41.47	35.50
Per Capita Expenditure for Processed F&V	PCEX_PFV	£	26.35	19.12	29.76	21.57
Expenditure for Fresh F&V/ Total F&V Exp.	SHARE_F	Share	0.55	0.18	0.55	0.18
Per Capita Total Grocery Expenditure	PCEX_TOT	£	366.38	204.04	418.88	230.40
Exp. Convenience Stores/Total Grocery Exp.	CONV	Share	0.07	0.12	0.07	0.13
Exp. Discount Stores /Total Grocery Exp.	DISC	Share	0.20	0.26	0.20	0.27
Exp. Large Stores / /Total Grocery Exp.	LARGE	Share	0.58	0.31	0.54	0.33
Exp. from CB & Other Stores/Total Gr. Exp.	OTHER	Share	0.07	0.09	0.06	0.10
Online Expenditure/Total Grocery Exp.	ONLINE	Share	0.08	0.22	0.13	0.28
Age of Primary Shopper	AGE	Years	52.91	13.17	52.91	13.17
Sex of Primary Shopper (1 = male)	SEX	Binary	0.27	0.44	0.27	0.44
Number of Children in the Household	NCH	Number	0.49	0.88	0.49	0.88
Number of Adults in the Household	NAD	Number	2.20	2.73	2.20	2.73

*Before Covid-19 period ranges from first quarter 2019 to first quarter 2020 (included), After Covid-19 period ranges from second quarter 2020 to fourth quarter 2020*.

To identify the factors affecting the change in expenditure, a set of three regressions were run, with dependent variables being per capita expenditure for Fresh Fruit and Vegetables (PCEX_F), per capita expenditure for Processed Fruit (PCEX_P) and Vegetables and expenditure share of Fresh Fruit and Vegetables on total expenditure for fruit and vegetables (SHARE_F). Table 5 reports the results. It must be noted that the regression of the fresh fruit and vegetable share (Equation 3) reports a very low value of *R*^2^ suggesting caution in the interpretation of the results from this estimation. The fitting of the other two regression is satisfactory, the *R*^2^ being equal to 0.36 in the case of expenditure for Fresh Fruit and Vegetables and 0.41 for Processed Fruit and Vegetables.

**Table 5:** Results of the regressions of per capita expenditure for fresh fruit and vegetables (PCEX_F), per capita expenditure for processed fruit and vegetables (PCEX_P) and share of expenditure for fresh fruit and vegetables on total expenditure for fruit and vegetables (SHARE_F).

		**Regression 1 PCEX_F**			**Regression 2 PCEX_P**			**Regression 3 SHARE_F**		
		***R*** **^2^: 0.361**			***R*** **^2^: 0.407**			***R*** **^2^: 0.058**		
		Wald χ^2^: 10311.02^(***)^			Wald χ^2^: 7591.15^(***)^			Wald χ^2^: 6584.78^(***)^		
**Description**	**Variables**	**Coeff**.	**S.E**.	***P*** **-value**	**Coeff**.	**S.E**.	***P*** **-value**	**Coeff**.	**S.E**.	***P*** **-value**
Per capita total grocery expenditure	PCEX_T	0.080	0.002	0.000^(***)^	0.060	0.001	0.000^(***)^	−0.002	0.000	0.000^(***)^
Exp. convenience stores/total grocery exp.	CONV	−5.761	2.536	0.023^(**)^	−6.551	0.980	0.000^(***)^	1.635	0.907	0.072^(*)^
Exp. discount stores/total grocery exp.	DISC	4.673	0.721	0.000^(***)^	−3.923	0.481	0.000^(***)^	8.497	0.515	0.000^(***)^
Exp. large stores/total grocery exp.	LARGE	−1.491	0.643	0.020^(**)^	−2.416	0.413	0.000^(***)^	1.028	0.418	0.014^(**)^
Exp. from CB & Other Stores/Total Gr. Exp.	OTHER	−18.632	1.608	0.000^(***)^	−10.280	0.867	0.000^(***)^	−6.366	1.021	0.000^(***)^
2 ^nd^ Quarter 2019 (binary variable)	Q2_19_	2.102	0.103	0.000^(***)^	−0.606	0.074	0.000^(***)^	1.984	0.082	0.000^(***)^
3 ^rd^ Quarter 2019 (binary variable)	Q3_19_	1.308	0.120	0.000^(***)^	−0.924	0.079	0.000^(***)^	1.646	0.089	0.000^(***)^
4 ^th^ Quarter 2019 (binary variable)	Q4_19_	−6.387	0.110	0.000^(***)^	−1.373	0.080	0.000^(***)^	−3.088	0.085	0.000^(***)^
Interactions with the indicator (binary variable) *a*	PCEX_T × *a*	−0.003	0.001	0.003^(***)^	−0.002	0.001	0.006^(***)^	0.000	0.000	0.702
identifying the post-COVID-19 periods	CONV × *a*	3.651	1.692	0.031^(**)^	1.510	0.949	0.111	1.113	0.821	0.175
	DISC × *a*	−0.956	0.597	0.109	−0.598	0.418	0.152	−1.024	0.420	0.015^(**)^
	LARGE × *a*	−1.019	0.548	0.063^(*)^	−0.239	0.405	0.555	−0.295	0.367	0.422
	OTHER × *a*	−3.275	1.176	0.005^(***)^	−1.663	0.828	0.045^(**)^	0.367	0.976	0.707
2 ^nd^ Quarter 2020 (binary variable)	Q2_20_	3.549	0.621	0.000^(***)^	1.835	0.432	0.000^(***)^	0.334	0.380	0.380
3 ^rd^ Quarter 2020 (binary variable)	Q3_20_	1.050	0.619	0.090^(*)^	1.306	0.426	0.002^(***)^	−0.610	0.377	0.105
4 ^*th*^ Quarter 2020 (binary variable)	Q4_20_	1.713	0.648	0.008^(***)^	1.573	0.444	0.000^(***)^	−0.134	0.379	0.723
Age of Primary Shopper	AGE	0.157	0.023	0.000^(***)^	−0.129	0.012	0.000^(***)^	0.198	0.013	0.000^(***)^
Sex of Primary Shopper (1 = male)	SEX	−1.816	0.557	0.001^(***)^	1.219	0.311	0.000^(***)^	−2.538	0.333	0.000^(***)^
Number of children in the household	NCH	−1.510	0.230	0.000^(***)^	−1.043	0.136	0.000^(***)^	−0.173	0.181	0.337
Number of Adults in the Household	NAD	−3.060	0.267	0.000^(***)^	−1.381	0.151	0.000^(***)^	−0.451	0.177	0.011^(**)^
	Constant	11.490	1.800	0.000^(***)^	17.141	1.065	0.000^(***)^	47.777	1.059	0.000^(***)^

Asterisks indicates coefficients that are statistically significant at 90% (*), 95% (**), or 99% (***) confidence level.

The products of the indicator variable *a* (identifying quarters in the post-COVID-19 period) with the variables related to per capita expenditure for all grocery goods (PCEX_TOT) and to the share of total grocery expenditure by store type (CONV, DISC, LARGE and OTHER) have been added to the model in order to test the hypothesis as described in the methodological section.

#### Measuring Existence and Magnitude of Stay-Home Lifestyle Response

The regression confirmed that the expenditure for both types of fruit and vegetables increase with the total expenditure for grocery. Before COVID-19, an additional pound of grocery expenditure resulted in 8 pence increase in fresh fruit and vegetables expenditure and in 6 pence increase in processed fruit and vegetables. The figures are roughly consistent with the observed 18% share of the grocery budget for both categories (Figure 5). After COVID-19, the marginal effect of an increase in grocery expenditure decreased by a small but statistically significant amount (0.3 and 0.2 pence for Fresh and Processed Fruit and Vegetables, respectively).

An increase of £1 in grocery expenditure has a negative but extremely small effect in the share of fresh fruit and vegetables on total fruit and vegetables (Table 6 using regression 3). The effect did not change after COVID-19. Based on the regression results, we can conclude that a limited effect of Stay-Home Lifestyle response was detected.

**Table 6:** Expected change in expenditure for fresh and processed fruit and vegetables due to a unit increase in online expenditure share for grocery products and an equal amount reduction in the share of other types of stores (values in £).

	**Fresh F&V**		**Processed F&V**	
**A unit Reduction**	**Bef. Covid**	**After Covid**	**Bef. Covid**	**After Covid**
Convenience stores	5.761	2.11^(**)^	6.55	5.04
Discount stores	−4.673	−3.72	3.92	4.52
Large Stores	1.491	2.51^(*)^	2.416	2.66
Other Stores	18.632	21.91^(***)^	10.280	11.94^(**)^

-The difference between before and after Covid-19 is statistically significant at: ^(*)^ 90% confidence level, ^(**)^ 95% confidence level, ^(***)^ 99% confidence level.

*-Before Covid-19 period ranges from first quarter 2019 to first quarter 2020 (included), After Covid-19 period ranges from second quarter 2020 to fourth quarter 2020*.

*-Figures in the table report the expected (average) change in expenditure due to a change in a household choice of shopping type. The results simulate the hypothetical effect on fruit and vegetable consumption of changing the usual shopping outlets because of the pandemic*.

### Measuring Existence and Magnitude of the Pandemic Shopping Response

The choice of store type has a statistically significant impact on the expenditure for fruit and vegetables. The coefficients can be interpreted as the effect on fruit and vegetables expenditure of increasing the share of grocery expenditure in the store type by 1, while reducing the share of online grocery expenditure by the same amount. Note that because a linear model was used, the opposite of the coefficient estimate provides an estimation of the change in fruit and vegetables expenditure due to an increase in online expenditure share obtained reducing the expenditure in a given store type by the same amount.

Increase in online purchase is associated with an increase in the expenditure for fruit and vegetables, with the only exception of the case of fresh products and a reduction in expenditure at discount stores. The COVID-19 outbreak did not affect the results for processed fruit and vegetables and had a limited effect on fresh products. In general, increasing in online purchase is associated with decrease in the expenditure share of fresh fruit and vegetables, relative to processed ones (Table 5 using regression 3). COVID-19 did not alter this trend. Based on the regression results we concluded that Pandemic Shopping response affected the expenditure for fruit and vegetables.

### Measuring Existence and Magnitude of the Mood and Attitude Response

Table 7 reports the results of the comparison of the coefficients of seasonal binary variables. Each 2020 variable has been compared with the same quarter in 2019 in order to identify changes after the Covid-19 outbreak that were not captured by Lifestyle or Pandemic Shopping Responses. The regressions found a positive effect on the per-capita expenditure for Processed Fruit and Vegetables ranging from 2.2 pounds in summer (Q3) to almost 3 pounds in fall (Q4). The effect on fresh produce was moderate or insignificant in spring and fall but very large (£8) during fall. As a result of the two combined effect, the 2020 seasonal coefficients in regression 3 (expenditure share) were lower than the 2019 ones in spring and summer and higher in fall. Based on these results, it is possible to conclude that an effect of Mood and Attitude Response was detected.

**Table 7:** Differences in estimated coefficients of seasonal effects before and after Covid-19.

**Comparison**	**Regression 1 PCEX_F**	**Regression 2 PCEX_P**	**Regression 3 SHARE_F**
Q2_20_-Q2_19_	1.45^(**)^	2.44^(***)^	−1.65^(***)^
Q3_20_-Q3_19_	−0.26	2.23^(***)^	−2.26^(***)^
Q4_20_-Q4_19_	8.10^(***)^	2.95^(***)^	2.95^(***)^

The difference is statistically significant in a pairwise χ^2^ test on equality of coefficients at: ^(**)^ 95% confidence level, ^(***)^ 99% confidence level.

Finally, the regressions found that elder, female shoppers on average are expected to consume more fresh fruit and vegetables than younger male ones. Number of adults and children are negatively associated with expenditure for both types of fruit and vegetables, with larger households having on average lower expenditure share for fresh products.

## Discussion

The objective of this paper is to measure the impact of COVID-19 on the purchases of fruit and vegetables for at home consumption by UK households and to identify the driving factors the response.

Descriptive statistics from the UK sample data showed that the expenditure for at-home consumption of fruit and vegetables increased after the COVID-19 outbreak (Table 3). The 95% confidence interval of the percentage increase in expenditure ranged between 7.7 and 11.1% for fresh fruit and vegetables and between 12.0 and 15.6% for processed fruit and vegetables. The different rate of increase resulted in slight decrease of the sample expenditure share for fresh produce over the total expenditure for fruit and vegetables. The point estimate of the variation was−0.4% (from 55.4 before COVID-19 to 55.0% after COVID-19). However, this difference was statistically significant only at 90% confidence level and there is no strong statistical evidence supporting a change in the composition of the basket.

The relatively small average variations are the result of heterogeneous trends heading in conflicting directions. The analysis of household data showed that individual changes in fruit and vegetables purchase may be large, even if on the aggregate expenditure is relatively stable.

The At-Home Lifestyle response (measured as changes in total grocery expenditure) was the main driver of the changes in expenditure. Changes in per capita expenditure for all grocery products explain 46% of the variation in the per capita expenditure for at home consumption of fruit and vegetables alone.

On average, before COVID-19 for each additional £1 of grocery expenditure there was 8 pence increase in expenditure for fresh fruit and vegetables produce and 6 pence increase for processed fruit and vegetables. However, after COVID-19 there was a small but statistically significant reduction in both marginal effects. This result implies that stay-home habit led on average to a lower-than-expected change in the dependent variables. Based on the available data, the increases in per capita expenditure for fresh and processed fruit and vegetables were 3.75 and 3.3% lower than what they would be expected based on pre-COVID-19 trends, respectively.

Pandemic Shopping response to COVID-19, on average, helped consumers dealing with limitations to mobility, at least partially. By shopping online, UK consumers were able to keep their expenditure for fruit and vegetables. The overall effect is consistent with an acceleration of the pre-COVID-19 trend. More households buy online because of the pandemic, but once they log on the website their purchasing behavior is similar to the pre-COVID-19 online shoppers. A partial exception might concern the increasing online purchase of fresh fruit and vegetables, but statistical evidence is mixed in this regard. An important exception to the general trend is that substituting purchases at discount stores with online shopping is expected to reduce per capita expenditure of fresh fruit and vegetables. This result suggests that price-sensitive consumers might have a different approach to online shopping of fruit and vegetables than others.

A set of binary variables identifying each quarter after COVID-19 was used to measure the effects of Mood and Attitude response of UK consumers. The systematic effect that was not explained by the Lifestyle and Pandemic Shopping responses or by other variables in the model was attributed to the consequences of COVID-19 psychological pressure on consumers. Based on the results from Table 7, the estimates of the effect of this Response type range between 8 and 11% of average per capita expenditure before COVID-19.

The effect was almost constant over the entire study period as far as processed fruit and vegetables are concerned. The result is consistent with a hoarding effect, leading consumers to increase their expenditure for non-perishable products. In this way, consumers stockpile products to cover for possible future shortage.

Instead, in the case of fresh fruit and vegetables, effect was small or insignificant during the first two quarters after the pandemic outbreak and very large (£8) in fall 2020. The result might be driven by health concerns as the second wave of contagion was approaching, but more research is needed in this regard.

The increase in online shopping was associated with increases in per capita expenditure for fruit and vegetables. This result is of particular importance, given the concerns that movement limitations during the pandemic may have negative consequences on nutrition. However, the positive effect of online purchases was stronger in the case of expenditure for processed fruit and vegetables.

Finally, we acknowledge that the study has three main limitations. Firstly, it considers only at-home consumption. Therefore, it is not possible to compute the actual change in consumers' diet because away from home consumption before COVID-19 was not observed. Secondly, the dataset used quarterly data and was unable to break the time period according to the exact developments in the pandemic emergency. For example, the outbreak happened in the UK during February 2020 and most of the containment measures were adopted in March. This implies that the first quarter 2020 included data both before and after COVID-19, making interpretation of the results difficult. More comprehensive and detailed datasets may be used in future research to confirm the results. Finally, predictive power of the regressions of the share of fresh fruit and vegetable on total fruit and vegetables expenditure (Table 5) is very low. This result suggests special caution in the interpretation of the factors affecting the substitution between fresh and processed products.

## Data Availability Statement

The data analyzed in this study is subject to the following licenses/restrictions: The Kantar Worldpanel dataset is a proprietorial dataset and cannot be made available but we have included descriptive statistics in the Annex. Requests to access these datasets should be directed to cesar.revoredo@sruc.ac.uk.

## Ethics Statement

Ethical approval on human participants was not provided for this study because the data used in this research were secondary data (home scan panel data). The researchers had no influence on the manner in which the data were collected.

## Author Contributions

CR-G: conceptualization, data preparation, investigation, visualization, formal analysis, writing—original draft, and writing—review and editing. CR: conceptualization, investigation, writing—original draft, and writing—review and editing. ET: conceptualization, investigation, and formal analysis. All authors contributed to the article and approved the submitted version.

## Funding

This work was supported as part of the Strategic Research Programme of the Scottish Government Rural and Environment Science and Analytical Services (RESAS) division, Theme 2-Productive and Sustainable Land Management and Rural Economies (Work package 2.4 on Rural Industries) and Theme 3-Food, Health and Wellbeing (Work package 3.3 on Food Security).

## Conflict of Interest

The authors declare that the research was conducted in the absence of any commercial or financial relationships that could be construed as a potential conflict of interest.

## Publisher's Note

All claims expressed in this article are solely those of the authors and do not necessarily represent those of their affiliated organizations, or those of the publisher, the editors and the reviewers. Any product that may be evaluated in this article, or claim that may be made by its manufacturer, is not guaranteed or endorsed by the publisher.
