# Supplementary material for: Purchases of Fruit and Vegetables for at Home Consumption During COVID-19 in the UK: Trends and Determinants
Source: Front Nutr. 2022 Apr 1;9:847996. doi: 10.3389/fnut.2022.847996 (PMC9012448; doi:10.3389/fnut.2022.847996)
Supplement: Supplementary file 1 [file Data_Sheet_1.PDF]

## ANNEX

## Table A1. East region - Average per capita purchases of fruit and vegetables before and after Covid-19 lockdown

| Retailer type | Period | Statistic | Fruit and vegetable products (grams/per capita per week) | | | | | | | | | | | Total |
|---|---|---|---|---|---|---|---|---|---|---|---|---|---|---|
| | | | Potatoes | | | Vegetables (excl. potatoes) | | | | Fruit | | | | |
| | | | **Total** | Fresh | Processed | **Total** | Fresh green | Other fresh | Processed vegetables | **Total** | Fresh fruit | Processed fruit | Fruit juices | |
| **Club and bargain store** | All | Mean | 9.91 | 4.46 | 5.45 | 13.78 | 1.03 | 1.18 | 11.56 | 10.92 | 4.16 | 3.82 | 2.94 | 34.61 |
| | | St. dev. | 49.61 | 36.22 | 28.14 | 58.05 | 13.59 | 13.96 | 11.56 | 57.25 | 29.73 | 20.92 | 38.76 | 125.02 |
| | | Obs. | 10,792 | 10,792 | 10,792 | 10,792 | 10,792 | 10,792 | 10,792 | 10,792 | 10,792 | 10,792 | 10,792 | 10,792 |
| | Before Covid | Mean | 9.85 | 4.60 | 5.25 | 13.39 | 1.10 | 1.18 | 11.12 | 10.81 | 4.25 | 3.99 | 2.57 | 34.05 |
| | | St. dev. | 50.35 | 37.78 | 27.56 | 56.40 | 14.59 | 13.57 | 11.12 | 55.11 | 31.79 | 23.28 | 32.67 | 124.50 |
| | | Obs. | 6,745 | 6,745 | 6,745 | 6,745 | 6,745 | 6,745 | 6,745 | 6,745 | 6,745 | 6,745 | 6,745 | 6,745 |
| | After Covid | Mean | 10.02 | 4.24 | 5.78 | 14.41 | .92 | 1.19 | 12.30 | 11.11 | 4.02 | 3.55 | 3.55 | 35.54 |
| | | St. dev. | 48.38 | 33.45 | 29.07 | 60.70 | 11.73 | 14.59 | 12.30 | 60.65 | 25.94 | 16.24 | 47.19 | 125.89 |
| | | Obs. | 4,047 | 4,047 | 4,047 | 4,047 | 4,047 | 4,047 | 4,047 | 4,047 | 4,047 | 4,047 | 4,047 | 4,047 |
| **Convenience** | All | Mean | 25.23 | 17.87 | 7.35 | 56.77 | 19.86 | 22.77 | 14.15 | 58.30 | 45.20 | 4.17 | 8.93 | 140.30 |
| | | St. dev. | 92.59 | 76.05 | 35.84 | 198.34 | 88.52 | 86.89 | 14.15 | 224.76 | 200.14 | 26.77 | 63.67 | 442.41 |
| | | Obs. | 10,792 | 10,792 | 10,792 | 10,792 | 10,792 | 10,792 | 10,792 | 10,792 | 10,792 | 10,792 | 10,792 | 10,792 |
| | Before Covid | Mean | 23.48 | 16.36 | 7.12 | 48.53 | 16.94 | 18.60 | 13.00 | 48.47 | 36.70 | 3.82 | 7.95 | 120.48 |
| | | St. dev. | 88.66 | 71.77 | 36.85 | 172.16 | 80.60 | 69.96 | 13.00 | 181.80 | 154.04 | 18.04 | 56.97 | 376.85 |
| | | Obs. | 6,745 | 6,745 | 6,745 | 6,745 | 6,745 | 6,745 | 6,745 | 6,745 | 6,745 | 6,745 | 6,745 | 6,745 |
| | After Covid | Mean | 28.14 | 20.40 | 7.74 | 70.50 | 24.72 | 29.73 | 16.06 | 74.70 | 59.38 | 4.75 | 10.56 | 173.35 |
| | | St. dev. | 98.74 | 82.64 | 34.08 | 234.97 | 100.16 | 109.08 | 16.06 | 281.44 | 258.77 | 36.99 | 73.47 | 532.50 |
| | | Obs. | 4,047 | 4,047 | 4,047 | 4,047 | 4,047 | 4,047 | 4,047 | 4,047 | 4,047 | 4,047 | 4,047 | 4,047 |
| **Discounter** | All | Mean | 119.13 | 80.97 | 38.16 | 250.16 | 81.30 | 81.54 | 87.33 | 244.51 | 166.55 | 25.35 | 52.60 | 613.80 |
| | | St. dev. | 237.07 | 182.82 | 90.40 | 449.15 | 169.32 | 159.81 | 87.33 | 515.86 | 389.18 | 64.03 | 179.60 | 1,065.49 |
| | | Obs. | 10,792 | 10,792 | 10,792 | 10,792 | 10,792 | 10,792 | 10,792 | 10,792 | 10,792 | 10,792 | 10,792 | 10,792 |
| | Before Covid | Mean | 115.99 | 78.15 | 37.83 | 248.59 | 81.53 | 81.62 | 85.45 | 239.56 | 166.22 | 24.02 | 49.32 | 604.14 |
| | | St. dev. | 227.97 | 173.33 | 90.58 | 427.98 | 163.24 | 150.85 | 85.45 | 478.65 | 360.06 | 60.31 | 165.23 | 1,000.04 |
| | | Obs. | 6,745 | 6,745 | 6,745 | 6,745 | 6,745 | 6,745 | 6,745 | 6,745 | 6,745 | 6,745 | 6,745 | 6,745 |
| | After Covid | Mean | 124.37 | 85.66 | 38.71 | 252.77 | 80.91 | 81.40 | 90.46 | 252.76 | 167.10 | 27.58 | 58.08 | 629.90 |
| | | St. dev. | 251.46 | 197.56 | 90.12 | 482.42 | 179.01 | 173.73 | 90.46 | 572.49 | 433.44 | 69.73 | 201.18 | 1,166.39 |
| | | Obs. | 4,047 | 4,047 | 4,047 | 4,047 | 4,047 | 4,047 | 4,047 | 4,047 | 4,047 | 4,047 | 4,047 | 4,047 |
| Large store | All | Mean | 341.98 | 226.93 | 115.04 | 649.12 | 200.28 | 202.31 | 246.53 | 639.60 | 421.11 | 66.10 | 152.40 | 1,630.70 |
| | | St. dev. | 383.04 | 311.06 | 166.51 | 671.51 | 277.61 | 252.72 | 246.53 | 810.93 | 645.14 | 119.72 | 307.51 | 1,567.01 |
| | | Obs. | 10,792 | 10,792 | 10,792 | 10,792 | 10,792 | 10,792 | 10,792 | 10,792 | 10,792 | 10,792 | 10,792 | 10,792 |
| | Before Covid | Mean | 342.54 | 228.40 | 114.14 | 655.98 | 201.07 | 204.26 | 250.64 | 650.90 | 431.58 | 66.22 | 153.10 | 1,649.42 |
| | | St. dev. | 374.70 | 304.04 | 165.58 | 671.35 | 279.73 | 252.96 | 250.64 | 840.62 | 677.61 | 120.27 | 306.00 | 1,586.19 |
| | | Obs. | 6,745 | 6,745 | 6,745 | 6,745 | 6,745 | 6,745 | 6,745 | 6,745 | 6,745 | 6,745 | 6,745 | 6,745 |
| | After Covid | Mean | 341.04 | 224.49 | 116.55 | 637.68 | 198.95 | 199.06 | 239.67 | 620.77 | 403.67 | 65.88 | 151.22 | 1,599.49 |
| | | St. dev. | 396.59 | 322.43 | 168.05 | 671.71 | 274.07 | 252.32 | 239.67 | 758.60 | 586.72 | 118.81 | 310.04 | 1,534.20 |
| | | Obs. | 4,047 | 4,047 | 4,047 | 4,047 | 4,047 | 4,047 | 4,047 | 4,047 | 4,047 | 4,047 | 4,047 | 4,047 |

Continues

| Retailer type | Period | Statistic | Fruit and vegetable products (grams/per capita per week) | | | | | | | | | | | Total |
|---|---|---|---|---|---|---|---|---|---|---|---|---|---|---|
| | | | Potatoes | | | Vegetables (excl. potatoes) | | | | Fruit | | | | |
| | | | **Total** | Fresh | Processed | **Total** | Fresh green | Other fresh | Processed vegetables | **Total** | Fresh fruit | Processed fruit | Fruit juices | |
| **Online** | All | Mean | 80.96 | 51.59 | 29.37 | 169.55 | 51.95 | 50.53 | 67.07 | 154.46 | 95.57 | 16.67 | 42.21 | 404.97 |
| | | St. dev. | 228.02 | 164.36 | 96.64 | 456.74 | 187.66 | 146.28 | 67.07 | 446.89 | 308.69 | 68.65 | 172.20 | 1,012.40 |
| | | Obs. | 10,792 | 10,792 | 10,792 | 10,792 | 10,792 | 10,792 | 10,792 | 10,792 | 10,792 | 10,792 | 10,792 | 10,792 |
| | Before Covid | Mean | 63.65 | 40.12 | 23.53 | 135.31 | 40.46 | 39.26 | 55.59 | 120.13 | 74.09 | 12.91 | 33.12 | 319.09 |
| | | St. dev. | 195.44 | 140.31 | 83.54 | 383.28 | 145.51 | 122.48 | 55.59 | 373.24 | 258.37 | 57.80 | 143.36 | 854.35 |
| | | Obs. | 6,745 | 6,745 | 6,745 | 6,745 | 6,745 | 6,745 | 6,745 | 6,745 | 6,745 | 6,745 | 6,745 | 6,745 |
| | After Covid | Mean | 109.81 | 70.70 | 39.11 | 226.62 | 71.11 | 69.32 | 86.19 | 211.67 | 131.37 | 22.94 | 57.37 | 548.10 |
| | | St. dev. | 271.42 | 196.60 | 114.57 | 553.45 | 240.92 | 177.47 | 86.19 | 543.32 | 375.28 | 83.29 | 210.85 | 1,218.27 |
| | | Obs. | 4,047 | 4,047 | 4,047 | 4,047 | 4,047 | 4,047 | 4,047 | 4,047 | 4,047 | 4,047 | 4,047 | 4,047 |
| **Other retailer** | All | Mean | .79 | .63 | .16 | 2.60 | 1.12 | 1.24 | .24 | 2.40 | 1.73 | .11 | .56 | 5.80 |
| | | St. dev. | 7.80 | 7.22 | 2.60 | 29.45 | 13.54 | 16.37 | .24 | 38.62 | 34.00 | 2.08 | 13.94 | 67.90 |
| | | Obs. | 10,792 | 10,792 | 10,792 | 10,792 | 10,792 | 10,792 | 10,792 | 10,792 | 10,792 | 10,792 | 10,792 | 10,792 |
| | Before Covid | Mean | .66 | .52 | .15 | 1.93 | .85 | .81 | .27 | 1.62 | 1.02 | .13 | .47 | 4.22 |
| | | St. dev. | 6.80 | 6.23 | 2.38 | 17.29 | 8.49 | 9.02 | .27 | 20.59 | 13.40 | 2.33 | 13.61 | 35.59 |
| | | Obs. | 6,745 | 6,745 | 6,745 | 6,745 | 6,745 | 6,745 | 6,745 | 6,745 | 6,745 | 6,745 | 6,745 | 6,745 |
| | After Covid | Mean | 1.02 | .83 | .18 | 3.72 | 1.57 | 1.96 | .19 | 3.69 | 2.91 | .08 | .70 | 8.43 |
| | | St. dev. | 9.22 | 8.62 | 2.93 | 42.57 | 19.19 | 24.05 | .19 | 57.18 | 52.74 | 1.57 | 14.46 | 100.87 |
| | | Obs. | 4,047 | 4,047 | 4,047 | 4,047 | 4,047 | 4,047 | 4,047 | 4,047 | 4,047 | 4,047 | 4,047 | 4,047 |
| **All retailers** | All | Mean | 578.00 | 382.46 | 195.54 | 1,141.98 | 355.53 | 359.58 | 426.87 | 1,110.19 | 734.33 | 116.23 | 259.64 | 2,830.18 |
| | | St. dev. | 437.73 | 367.72 | 206.90 | 793.05 | 366.23 | 305.16 | 426.87 | 1,022.90 | 851.32 | 155.08 | 393.82 | 1,750.30 |
| | | Obs. | 10,792 | 10,792 | 10,792 | 10,792 | 10,792 | 10,792 | 10,792 | 10,792 | 10,792 | 10,792 | 10,792 | 10,792 |
| | Before Covid | Mean | 556.17 | 368.14 | 188.03 | 1,103.74 | 341.94 | 345.73 | 416.07 | 1,071.48 | 713.86 | 111.09 | 246.53 | 2,731.40 |
| | | St. dev. | 424.04 | 351.80 | 205.52 | 766.49 | 350.26 | 293.60 | 416.07 | 1,000.72 | 834.76 | 149.21 | 374.70 | 1,709.12 |
| | | Obs. | 6,745 | 6,745 | 6,745 | 6,745 | 6,745 | 6,745 | 6,745 | 6,745 | 6,745 | 6,745 | 6,745 | 6,745 |
| | After Covid | Mean | 614.39 | 406.32 | 208.07 | 1,205.71 | 378.18 | 382.65 | 444.88 | 1,174.71 | 768.44 | 124.79 | 281.47 | 2,994.81 |
| | | St. dev. | 457.38 | 391.71 | 208.59 | 831.65 | 390.40 | 322.23 | 444.88 | 1,055.80 | 877.28 | 164.07 | 422.93 | 1,805.11 |
| | | Obs. | 4,047 | 4,047 | 4,047 | 4,047 | 4,047 | 4,047 | 4,047 | 4,047 | 4,047 | 4,047 | 4,047 | 4,047 |

Source: Own elaboration based on Kantar Worldpanel data.

# Table A2. East region - Average per capita expenditure of fruit and vegetables before and after Covid-19 lockdown

| Retailer type | Period | Statistic | Fruit and vegetable products (pence/per capita per week) | | | | | | | | | | | Total |
|---|---|---|---|---|---|---|---|---|---|---|---|---|---|---|
| | | | Potatoes | | | Vegetables (excl. potatoes) | | | | Fruit | | | | |
| | | | **Total** | Fresh | Processed | **Total** | Fresh green | Other fresh | Processed vegetables | **Total** | Fresh fruit | Processed fruit | Fruit juices | |
| **Club and bargain store** | All | Mean | 2.06 | .28 | 1.78 | 2.46 | .30 | .35 | 1.81 | 2.96 | .93 | 1.79 | .24 | 7.48 |
| | | St. dev. | 7.94 | 2.28 | 7.21 | 11.08 | 3.85 | 4.38 | 1.81 | 12.89 | 6.92 | 9.00 | 2.73 | 24.45 |
| | | Obs. | 10,792 | 10,792 | 10,792 | 10,792 | 10,792 | 10,792 | 10,792 | 10,792 | 10,792 | 10,792 | 10,792 | 10,792 |
| | Before Covid | Mean | 2.02 | .29 | 1.74 | 2.36 | .32 | .33 | 1.71 | 3.01 | .92 | 1.89 | .21 | 7.39 |
| | | St. dev. | 7.77 | 2.37 | 7.09 | 10.82 | 4.29 | 4.00 | 1.71 | 13.57 | 6.93 | 9.90 | 2.31 | 24.41 |
| | | Obs. | 6,745 | 6,745 | 6,745 | 6,745 | 6,745 | 6,745 | 6,745 | 6,745 | 6,745 | 6,745 | 6,745 | 6,745 |
| | After Covid | Mean | 2.13 | .27 | 1.86 | 2.62 | .25 | .38 | 1.99 | 2.86 | .94 | 1.63 | .29 | 7.61 |
| | | St. dev. | 8.22 | 2.13 | 7.42 | 11.51 | 2.98 | 4.96 | 1.99 | 11.65 | 6.91 | 7.26 | 3.30 | 24.51 |
| | | Obs. | 4,047 | 4,047 | 4,047 | 4,047 | 4,047 | 4,047 | 4,047 | 4,047 | 4,047 | 4,047 | 4,047 | 4,047 |
| **Convenience** | All | Mean | 4.73 | 1.78 | 2.95 | 15.22 | 4.50 | 5.10 | 5.62 | 16.17 | 11.85 | 3.00 | 1.31 | 36.12 |
| | | St. dev. | 16.66 | 7.73 | 12.98 | 49.53 | 18.42 | 18.46 | 5.62 | 55.04 | 46.29 | 14.76 | 9.17 | 104.11 |
| | | Obs. | 10,792 | 10,792 | 10,792 | 10,792 | 10,792 | 10,792 | 10,792 | 10,792 | 10,792 | 10,792 | 10,792 | 10,792 |
| | Before Covid | Mean | 4.48 | 1.69 | 2.79 | 13.20 | 3.86 | 4.18 | 5.15 | 13.97 | 9.91 | 2.85 | 1.21 | 31.64 |
| | | St. dev. | 16.63 | 7.71 | 13.08 | 43.76 | 16.67 | 15.52 | 5.15 | 47.83 | 38.81 | 13.36 | 9.17 | 91.31 |
| | | Obs. | 6,745 | 6,745 | 6,745 | 6,745 | 6,745 | 6,745 | 6,745 | 6,745 | 6,745 | 6,745 | 6,745 | 6,745 |
| | After Covid | Mean | 5.15 | 1.94 | 3.21 | 18.59 | 5.57 | 6.63 | 6.39 | 19.84 | 15.10 | 3.26 | 1.49 | 43.58 |
| | | St. dev. | 16.71 | 7.75 | 12.81 | 57.73 | 20.97 | 22.46 | 6.39 | 65.14 | 56.45 | 16.85 | 9.17 | 122.16 |
| | | Obs. | 4,047 | 4,047 | 4,047 | 4,047 | 4,047 | 4,047 | 4,047 | 4,047 | 4,047 | 4,047 | 4,047 | 4,047 |
| **Discounter** | All | Mean | 11.83 | 4.98 | 6.86 | 40.67 | 13.77 | 13.92 | 12.98 | 48.08 | 33.12 | 10.94 | 4.02 | 100.59 |
| | | St. dev. | 22.52 | 11.31 | 15.11 | 74.55 | 30.39 | 28.17 | 12.98 | 95.34 | 73.90 | 26.51 | 13.50 | 173.80 |
| | | Obs. | 10,792 | 10,792 | 10,792 | 10,792 | 10,792 | 10,792 | 10,792 | 10,792 | 10,792 | 10,792 | 10,792 | 10,792 |
| | Before Covid | Mean | 11.64 | 4.94 | 6.70 | 40.74 | 13.98 | 14.05 | 12.71 | 47.00 | 32.78 | 10.54 | 3.68 | 99.38 |
| | | St. dev. | 21.83 | 11.06 | 14.60 | 71.04 | 29.60 | 26.67 | 12.71 | 89.61 | 69.35 | 25.28 | 12.11 | 164.18 |
| | | Obs. | 6,745 | 6,745 | 6,745 | 6,745 | 6,745 | 6,745 | 6,745 | 6,745 | 6,745 | 6,745 | 6,745 | 6,745 |
| | After Covid | Mean | 12.16 | 5.05 | 7.11 | 40.56 | 13.43 | 13.70 | 13.43 | 49.87 | 33.70 | 11.59 | 4.58 | 102.60 |
| | | St. dev. | 23.62 | 11.72 | 15.92 | 80.06 | 31.66 | 30.51 | 13.43 | 104.18 | 80.94 | 28.43 | 15.54 | 188.76 |
| | | Obs. | 4,047 | 4,047 | 4,047 | 4,047 | 4,047 | 4,047 | 4,047 | 4,047 | 4,047 | 4,047 | 4,047 | 4,047 |
| **Large store** | All | Mean | 48.21 | 17.28 | 30.93 | 137.48 | 40.08 | 39.76 | 57.64 | 148.17 | 98.08 | 32.95 | 17.14 | 333.86 |
| | | St. dev. | 51.14 | 24.12 | 39.50 | 153.51 | 60.06 | 53.30 | 57.64 | 202.39 | 159.32 | 58.18 | 36.15 | 351.72 |
| | | Obs. | 10,792 | 10,792 | 10,792 | 10,792 | 10,792 | 10,792 | 10,792 | 10,792 | 10,792 | 10,792 | 10,792 | 10,792 |
| | Before Covid | Mean | 48.14 | 18.02 | 30.12 | 139.66 | 40.75 | 40.77 | 58.14 | 149.59 | 99.50 | 32.88 | 17.22 | 337.39 |
| | | St. dev. | 49.78 | 24.65 | 37.72 | 153.49 | 61.05 | 53.81 | 58.14 | 207.47 | 163.75 | 58.76 | 36.11 | 355.67 |
| | | Obs. | 6,745 | 6,745 | 6,745 | 6,745 | 6,745 | 6,745 | 6,745 | 6,745 | 6,745 | 6,745 | 6,745 | 6,745 |
| | After Covid | Mean | 48.31 | 16.04 | 32.27 | 133.86 | 38.97 | 38.08 | 56.81 | 145.81 | 95.73 | 33.06 | 17.02 | 327.98 |
| | | St. dev. | 53.35 | 23.15 | 42.26 | 153.49 | 58.37 | 52.38 | 56.81 | 193.62 | 151.63 | 57.22 | 36.22 | 345.00 |
| | | Obs. | 4,047 | 4,047 | 4,047 | 4,047 | 4,047 | 4,047 | 4,047 | 4,047 | 4,047 | 4,047 | 4,047 | 4,047 |

Continues

| Retailer type | Period | Statistic | Fruit and vegetable products (pence/per capita per week) | | | | | | | | | | | Total |
|---|---|---|---|---|---|---|---|---|---|---|---|---|---|---|
| | | | Potatoes | | | Vegetables (excl. potatoes) | | | | Fruit | | | | |
| | | | **Total** | Fresh | Processed | **Total** | Fresh green | Other fresh | Processed vegetables | **Total** | Fresh fruit | Processed fruit | Fruit juices | |
| **Online** | All | Mean | 12.19 | 4.07 | 8.12 | 36.69 | 10.91 | 10.12 | 15.66 | 34.74 | 21.94 | 8.10 | 4.70 | 83.62 |
| | | St. dev. | 33.84 | 13.09 | 25.41 | 107.98 | 45.47 | 32.49 | 15.66 | 105.72 | 74.21 | 31.56 | 21.05 | 224.73 |
| | | Obs. | 10,792 | 10,792 | 10,792 | 10,792 | 10,792 | 10,792 | 10,792 | 10,792 | 10,792 | 10,792 | 10,792 | 10,792 |
| | Before Covid | Mean | 9.71 | 3.29 | 6.43 | 29.47 | 8.74 | 8.06 | 12.67 | 27.17 | 17.33 | 6.40 | 3.44 | 66.36 |
| | | St. dev. | 29.11 | 11.51 | 21.85 | 91.50 | 35.83 | 28.66 | 12.67 | 90.39 | 64.46 | 28.49 | 15.31 | 191.62 |
| | | Obs. | 6,745 | 6,745 | 6,745 | 6,745 | 6,745 | 6,745 | 6,745 | 6,745 | 6,745 | 6,745 | 6,745 | 6,745 |
| | After Covid | Mean | 16.32 | 5.38 | 10.94 | 48.71 | 14.51 | 13.57 | 20.63 | 47.36 | 29.62 | 10.94 | 6.80 | 112.39 |
| | | St. dev. | 40.18 | 15.27 | 30.22 | 130.03 | 57.92 | 37.77 | 20.63 | 126.24 | 87.58 | 35.92 | 28.00 | 268.64 |
| | | Obs. | 4,047 | 4,047 | 4,047 | 4,047 | 4,047 | 4,047 | 4,047 | 4,047 | 4,047 | 4,047 | 4,047 | 4,047 |
| **Other retailer** | All | Mean | .15 | .06 | .10 | .50 | .22 | .21 | .07 | .41 | .29 | .06 | .06 | 1.06 |
| | | St. dev. | 1.61 | .72 | 1.40 | 5.92 | 3.08 | 2.91 | .07 | 6.33 | 5.83 | .91 | 1.56 | 12.28 |
| | | Obs. | 10,792 | 10,792 | 10,792 | 10,792 | 10,792 | 10,792 | 10,792 | 10,792 | 10,792 | 10,792 | 10,792 | 10,792 |
| | Before Covid | Mean | .14 | .05 | .09 | .38 | .16 | .15 | .06 | .27 | .16 | .05 | .06 | .79 |
| | | St. dev. | 1.64 | .71 | 1.46 | 3.22 | 1.66 | 1.62 | .06 | 2.97 | 2.08 | .70 | 1.63 | 6.14 |
| | | Obs. | 6,745 | 6,745 | 6,745 | 6,745 | 6,745 | 6,745 | 6,745 | 6,745 | 6,745 | 6,745 | 6,745 | 6,745 |
| | After Covid | Mean | .17 | .07 | .10 | .70 | .31 | .32 | .07 | .63 | .49 | .06 | .07 | 1.51 |
| | | St. dev. | 1.55 | .75 | 1.30 | 8.73 | 4.54 | 4.26 | .07 | 9.59 | 9.13 | 1.19 | 1.45 | 18.41 |
| | | Obs. | 4,047 | 4,047 | 4,047 | 4,047 | 4,047 | 4,047 | 4,047 | 4,047 | 4,047 | 4,047 | 4,047 | 4,047 |
| **All retailers** | All | Mean | 79.18 | 28.45 | 50.73 | 233.02 | 69.78 | 69.47 | 93.77 | 250.52 | 166.22 | 56.83 | 27.48 | 562.72 |
| | | St. dev. | 57.98 | 28.22 | 48.35 | 185.41 | 80.93 | 65.45 | 93.77 | 244.89 | 198.76 | 73.56 | 44.56 | 399.40 |
| | | Obs. | 10,792 | 10,792 | 10,792 | 10,792 | 10,792 | 10,792 | 10,792 | 10,792 | 10,792 | 10,792 | 10,792 | 10,792 |
| | Before Covid | Mean | 76.14 | 28.27 | 47.87 | 225.80 | 67.82 | 67.54 | 90.44 | 241.02 | 160.60 | 54.60 | 25.81 | 542.95 |
| | | St. dev. | 56.16 | 28.16 | 46.43 | 178.39 | 77.27 | 63.83 | 90.44 | 243.94 | 197.45 | 73.20 | 41.91 | 393.47 |
| | | Obs. | 6,745 | 6,745 | 6,745 | 6,745 | 6,745 | 6,745 | 6,745 | 6,745 | 6,745 | 6,745 | 6,745 | 6,745 |
| | After Covid | Mean | 84.25 | 28.74 | 55.51 | 245.05 | 73.05 | 72.69 | 99.32 | 266.37 | 175.57 | 60.55 | 30.25 | 595.67 |
| | | St. dev. | 60.55 | 28.32 | 51.04 | 195.99 | 86.61 | 67.94 | 99.32 | 245.66 | 200.61 | 74.03 | 48.55 | 407.02 |
| | | Obs. | 4,047 | 4,047 | 4,047 | 4,047 | 4,047 | 4,047 | 4,047 | 4,047 | 4,047 | 4,047 | 4,047 | 4,047 |

Source: Own elaboration based on Kantar Worldpanel data.

## Table A3. London - Average per capita purchases of fruit and vegetables before and after Covid-19 lockdown

| **Retailer type** | **Period** | **Statistic** | **Fruit and vegetable products (grams/per capita per week)** | | | | | | | | | | | **Total** |
|---|---|---|---|---|---|---|---|---|---|---|---|---|---|---|
| | | | **Potatoes** | | | **Vegetables (excl. potatoes)** | | | | **Fruit** | | | | |
| | | | **Total** | Fresh | Processed | **Total** | Fresh green | Other fresh | Processed vegetables | **Total** | Fresh fruit | Processed fruit | Fruit juices | |
| **Club and bargain store** | All | Mean | 5.78 | 2.85 | 2.93 | 7.08 | .82 | 1.53 | 4.73 | 9.91 | 4.75 | 3.28 | 1.89 | 22.78 |
| | | St. dev. | 34.77 | 25.89 | 22.03 | 40.50 | 10.19 | 17.99 | 4.73 | 60.06 | 42.46 | 18.40 | 20.74 | 109.25 |
| | | Obs. | 7,304 | 7,304 | 7,304 | 7,304 | 7,304 | 7,304 | 7,304 | 7,304 | 7,304 | 7,304 | 7,304 | 7,304 |
| | Before Covid | Mean | 5.41 | 2.64 | 2.77 | 6.83 | .80 | 1.38 | 4.64 | 8.87 | 4.25 | 3.24 | 1.38 | 21.11 |
| | | St. dev. | 32.12 | 23.74 | 20.60 | 37.73 | 8.66 | 17.17 | 4.64 | 49.25 | 35.37 | 18.83 | 15.86 | 94.94 |
| | | Obs. | 4,565 | 4,565 | 4,565 | 4,565 | 4,565 | 4,565 | 4,565 | 4,565 | 4,565 | 4,565 | 4,565 | 4,565 |
| | After Covid | Mean | 6.41 | 3.20 | 3.21 | 7.51 | .86 | 1.77 | 4.88 | 11.66 | 5.59 | 3.34 | 2.72 | 25.57 |
| | | St. dev. | 38.78 | 29.12 | 24.23 | 44.74 | 12.34 | 19.29 | 4.88 | 74.65 | 52.17 | 17.67 | 26.96 | 129.61 |
| | | Obs. | 2,739 | 2,739 | 2,739 | 2,739 | 2,739 | 2,739 | 2,739 | 2,739 | 2,739 | 2,739 | 2,739 | 2,739 |
| **Convenience** | All | Mean | 28.34 | 21.49 | 6.86 | 76.79 | 26.01 | 29.55 | 21.22 | 106.40 | 82.76 | 6.55 | 17.10 | 211.54 |
| | | St. dev. | 90.58 | 77.72 | 30.10 | 208.54 | 88.28 | 94.92 | 21.22 | 291.38 | 245.92 | 28.44 | 99.53 | 506.58 |
| | | Obs. | 7,304 | 7,304 | 7,304 | 7,304 | 7,304 | 7,304 | 7,304 | 7,304 | 7,304 | 7,304 | 7,304 | 7,304 |
| | Before Covid | Mean | 25.35 | 19.12 | 6.22 | 70.90 | 24.29 | 26.70 | 19.91 | 99.65 | 78.01 | 6.05 | 15.59 | 195.90 |
| | | St. dev. | 83.65 | 72.42 | 28.50 | 196.09 | 83.44 | 88.65 | 19.91 | 275.41 | 236.13 | 25.89 | 91.35 | 471.22 |
| | | Obs. | 4,565 | 4,565 | 4,565 | 4,565 | 4,565 | 4,565 | 4,565 | 4,565 | 4,565 | 4,565 | 4,565 | 4,565 |
| | After Covid | Mean | 33.33 | 25.43 | 7.91 | 86.60 | 28.88 | 34.31 | 23.41 | 117.66 | 90.68 | 7.38 | 19.61 | 237.60 |
| | | St. dev. | 100.90 | 85.70 | 32.58 | 227.49 | 95.75 | 104.38 | 23.41 | 315.96 | 261.28 | 32.22 | 111.82 | 559.70 |
| | | Obs. | 2,739 | 2,739 | 2,739 | 2,739 | 2,739 | 2,739 | 2,739 | 2,739 | 2,739 | 2,739 | 2,739 | 2,739 |
| **Discounter** | All | Mean | 82.64 | 58.51 | 24.14 | 217.66 | 79.50 | 69.66 | 68.50 | 223.91 | 147.12 | 25.27 | 51.51 | 524.21 |
| | | St. dev. | 184.70 | 150.70 | 65.70 | 427.71 | 182.15 | 151.45 | 68.50 | 475.68 | 343.02 | 63.74 | 220.65 | 962.75 |
| | | Obs. | 7,304 | 7,304 | 7,304 | 7,304 | 7,304 | 7,304 | 7,304 | 7,304 | 7,304 | 7,304 | 7,304 | 7,304 |
| | Before Covid | Mean | 81.34 | 57.98 | 23.37 | 214.52 | 77.83 | 69.16 | 67.53 | 219.06 | 144.24 | 23.62 | 51.20 | 514.92 |
| | | St. dev. | 182.59 | 148.99 | 62.47 | 423.66 | 180.94 | 154.05 | 67.53 | 472.06 | 342.48 | 60.37 | 219.89 | 951.14 |
| | | Obs. | 4,565 | 4,565 | 4,565 | 4,565 | 4,565 | 4,565 | 4,565 | 4,565 | 4,565 | 4,565 | 4,565 | 4,565 |
| | After Covid | Mean | 84.81 | 59.39 | 25.42 | 222.90 | 82.30 | 70.50 | 70.11 | 231.98 | 151.93 | 28.02 | 52.03 | 539.70 |
| | | St. dev. | 188.20 | 153.53 | 70.76 | 434.40 | 184.17 | 147.04 | 70.11 | 481.63 | 343.93 | 68.91 | 221.94 | 981.76 |
| | | Obs. | 2,739 | 2,739 | 2,739 | 2,739 | 2,739 | 2,739 | 2,739 | 2,739 | 2,739 | 2,739 | 2,739 | 2,739 |
| **Large store** | All | Mean | 327.02 | 217.41 | 109.61 | 699.13 | 213.92 | 217.01 | 268.20 | 704.47 | 455.46 | 62.47 | 186.55 | 1,730.63 |
| | | St. dev. | 386.40 | 312.60 | 164.32 | 724.34 | 293.10 | 280.57 | 268.20 | 829.82 | 637.34 | 110.50 | 400.58 | 1,626.15 |
| | | Obs. | 7,304 | 7,304 | 7,304 | 7,304 | 7,304 | 7,304 | 7,304 | 7,304 | 7,304 | 7,304 | 7,304 | 7,304 |
| | Before Covid | Mean | 321.58 | 212.94 | 108.64 | 691.77 | 207.32 | 213.50 | 270.95 | 694.52 | 448.41 | 62.45 | 183.66 | 1,707.88 |
| | | St. dev. | 374.12 | 300.04 | 162.86 | 701.67 | 278.96 | 271.80 | 270.95 | 796.75 | 618.14 | 108.07 | 381.73 | 1,556.92 |
| | | Obs. | 4,565 | 4,565 | 4,565 | 4,565 | 4,565 | 4,565 | 4,565 | 4,565 | 4,565 | 4,565 | 4,565 | 4,565 |
| | After Covid | Mean | 336.09 | 224.86 | 111.23 | 711.39 | 224.91 | 222.85 | 263.62 | 721.07 | 467.21 | 62.50 | 191.36 | 1,768.54 |
| | | St. dev. | 405.95 | 332.39 | 166.74 | 760.60 | 315.01 | 294.57 | 263.62 | 882.08 | 668.08 | 114.45 | 430.20 | 1,735.04 |
| | | Obs. | 2,739 | 2,739 | 2,739 | 2,739 | 2,739 | 2,739 | 2,739 | 2,739 | 2,739 | 2,739 | 2,739 | 2,739 |

Continues

| Retailer type | Period | Statistic | Fruit and vegetable products (grams/per capita per week) | | | | | | | | | | | Total |
|---|---|---|---|---|---|---|---|---|---|---|---|---|---|---|
| | | | Potatoes | | | Vegetables (excl. potatoes) | | | | Fruit | | | | |
| | | | **Total** | Fresh | Processed | **Total** | Fresh green | Other fresh | Processed vegetables | **Total** | Fresh fruit | Processed fruit | Fruit juices | |
| **Online** | All | Mean | 58.37 | 41.26 | 17.10 | 147.41 | 41.82 | 42.78 | 62.81 | 128.63 | 75.02 | 12.97 | 40.64 | 334.40 |
| | | St. dev. | 191.06 | 156.99 | 58.75 | 485.93 | 151.52 | 153.04 | 62.81 | 400.76 | 264.84 | 62.34 | 175.97 | 958.05 |
| | | Obs. | 7,304 | 7,304 | 7,304 | 7,304 | 7,304 | 7,304 | 7,304 | 7,304 | 7,304 | 7,304 | 7,304 | 7,304 |
| | Before Covid | Mean | 43.68 | 30.16 | 13.53 | 112.65 | 29.06 | 31.01 | 52.58 | 95.38 | 54.65 | 9.07 | 31.66 | 251.71 |
| | | St. dev. | 149.93 | 120.57 | 50.68 | 408.20 | 109.10 | 121.60 | 52.58 | 319.45 | 216.12 | 47.52 | 145.64 | 773.97 |
| | | Obs. | 4,565 | 4,565 | 4,565 | 4,565 | 4,565 | 4,565 | 4,565 | 4,565 | 4,565 | 4,565 | 4,565 | 4,565 |
| | After Covid | Mean | 82.85 | 59.78 | 23.07 | 205.33 | 63.08 | 62.40 | 79.86 | 184.04 | 108.95 | 19.47 | 55.62 | 472.22 |
| | | St. dev. | 242.78 | 202.38 | 69.76 | 588.80 | 201.68 | 192.88 | 79.86 | 503.36 | 327.69 | 80.84 | 216.51 | 1,191.32 |
| | | Obs. | 2,739 | 2,739 | 2,739 | 2,739 | 2,739 | 2,739 | 2,739 | 2,739 | 2,739 | 2,739 | 2,739 | 2,739 |
| **Other retailer** | All | Mean | .81 | .45 | .36 | 2.77 | .87 | .97 | .93 | 5.28 | 2.21 | .27 | 2.79 | 8.86 |
| | | St. dev. | 9.88 | 6.13 | 6.81 | 25.79 | 11.19 | 10.41 | .93 | 58.34 | 32.08 | 4.38 | 46.50 | 78.79 |
| | | Obs. | 7,304 | 7,304 | 7,304 | 7,304 | 7,304 | 7,304 | 7,304 | 7,304 | 7,304 | 7,304 | 7,304 | 7,304 |
| | Before Covid | Mean | .71 | .35 | .36 | 2.38 | .70 | .82 | .86 | 5.05 | 1.66 | .25 | 3.14 | 8.14 |
| | | St. dev. | 8.91 | 4.49 | 7.22 | 21.98 | 9.57 | 8.51 | .86 | 53.82 | 21.74 | 3.61 | 48.18 | 69.61 |
| | | Obs. | 4,565 | 4,565 | 4,565 | 4,565 | 4,565 | 4,565 | 4,565 | 4,565 | 4,565 | 4,565 | 4,565 | 4,565 |
| | After Covid | Mean | .98 | .61 | .37 | 3.42 | 1.15 | 1.22 | 1.04 | 5.67 | 3.14 | .31 | 2.22 | 10.07 |
| | | St. dev. | 11.31 | 8.16 | 6.08 | 31.11 | 13.45 | 12.96 | 1.04 | 65.20 | 44.23 | 5.43 | 43.56 | 92.07 |
| | | Obs. | 2,739 | 2,739 | 2,739 | 2,739 | 2,739 | 2,739 | 2,739 | 2,739 | 2,739 | 2,739 | 2,739 | 2,739 |
| **All retailers** | All | Mean | 502.98 | 341.98 | 161.00 | 1,150.84 | 362.95 | 361.50 | 426.39 | 1,178.61 | 767.32 | 110.81 | 300.48 | 2,832.42 |
| | | St. dev. | 435.39 | 369.42 | 182.33 | 881.05 | 366.06 | 338.98 | 426.39 | 1,014.76 | 802.69 | 146.32 | 521.90 | 1,829.34 |
| | | Obs. | 7,304 | 7,304 | 7,304 | 7,304 | 7,304 | 7,304 | 7,304 | 7,304 | 7,304 | 7,304 | 7,304 | 7,304 |
| | Before Covid | Mean | 478.07 | 323.19 | 154.88 | 1,099.05 | 340.01 | 342.57 | 416.47 | 1,122.53 | 731.21 | 104.68 | 286.63 | 2,699.65 |
| | | St. dev. | 415.14 | 347.17 | 178.40 | 832.42 | 343.20 | 323.28 | 416.47 | 972.06 | 782.78 | 137.38 | 487.91 | 1,729.37 |
| | | Obs. | 4,565 | 4,565 | 4,565 | 4,565 | 4,565 | 4,565 | 4,565 | 4,565 | 4,565 | 4,565 | 4,565 | 4,565 |
| | After Covid | Mean | 544.48 | 373.28 | 171.20 | 1,237.15 | 401.18 | 393.05 | 442.93 | 1,272.07 | 827.50 | 121.01 | 323.56 | 3,053.70 |
| | | St. dev. | 464.32 | 401.89 | 188.28 | 950.53 | 398.43 | 361.51 | 442.93 | 1,075.90 | 831.47 | 159.64 | 573.44 | 1,965.28 |
| | | Obs. | 2,739 | 2,739 | 2,739 | 2,739 | 2,739 | 2,739 | 2,739 | 2,739 | 2,739 | 2,739 | 2,739 | 2,739 |

Source: Own elaboration based on Kantar Worldpanel data.

## Table A4. London - Average per capita expenditure of fruit and vegetables before and after Covid-19 lockdown

| Retailer type | Period | Statistic | Fruit and vegetable products (pence/per capita per week) | | | | | | | | | | | Total |
|---|---|---|---|---|---|---|---|---|---|---|---|---|---|---|
| | | | Potatoes | | | Vegetables (excl. potatoes) | | | | Fruit | | | | |
| | | | **Total** | Fresh | Processed | **Total** | Fresh green | Other fresh | Processed vegetables | **Total** | Fresh fruit | Processed fruit | Fruit juices | |
| **Club and bargain store** | All | Mean | 1.45 | .19 | 1.25 | 1.78 | .38 | .47 | .93 | 3.43 | 1.22 | 1.95 | .26 | 6.66 |
| | | St. dev. | 7.13 | 1.75 | 6.85 | 11.97 | 4.11 | 5.83 | .93 | 20.26 | 10.53 | 12.91 | 3.35 | 31.30 |
| | | Obs. | 7,304 | 7,304 | 7,304 | 7,304 | 7,304 | 7,304 | 7,304 | 7,304 | 7,304 | 7,304 | 7,304 | 7,304 |
| | Before Covid | Mean | 1.38 | .18 | 1.20 | 1.72 | .38 | .44 | .90 | 3.27 | 1.07 | 2.00 | .20 | 6.37 |
| | | St. dev. | 6.67 | 1.60 | 6.42 | 11.36 | 4.15 | 5.58 | .90 | 19.43 | 9.04 | 14.10 | 2.48 | 29.41 |
| | | Obs. | 4,565 | 4,565 | 4,565 | 4,565 | 4,565 | 4,565 | 4,565 | 4,565 | 4,565 | 4,565 | 4,565 | 4,565 |
| | After Covid | Mean | 1.56 | .22 | 1.34 | 1.87 | .37 | .53 | .98 | 3.69 | 1.47 | 1.85 | .37 | 7.13 |
| | | St. dev. | 7.83 | 1.97 | 7.50 | 12.91 | 4.05 | 6.24 | .98 | 21.58 | 12.64 | 10.64 | 4.43 | 34.23 |
| | | Obs. | 2,739 | 2,739 | 2,739 | 2,739 | 2,739 | 2,739 | 2,739 | 2,739 | 2,739 | 2,739 | 2,739 | 2,739 |
| **Convenience** | All | Mean | 5.57 | 2.21 | 3.36 | 24.71 | 7.07 | 7.40 | 10.24 | 30.91 | 22.47 | 5.70 | 2.74 | 61.19 |
| | | St. dev. | 17.18 | 8.28 | 13.52 | 70.10 | 23.47 | 23.86 | 10.24 | 94.09 | 73.54 | 26.61 | 16.88 | 161.49 |
| | | Obs. | 7,304 | 7,304 | 7,304 | 7,304 | 7,304 | 7,304 | 7,304 | 7,304 | 7,304 | 7,304 | 7,304 | 7,304 |
| | Before Covid | Mean | 5.02 | 2.02 | 3.00 | 23.01 | 6.64 | 6.61 | 9.76 | 28.00 | 20.28 | 5.30 | 2.42 | 56.04 |
| | | St. dev. | 15.32 | 7.71 | 11.85 | 65.73 | 22.17 | 21.89 | 9.76 | 85.80 | 68.45 | 24.22 | 14.32 | 146.34 |
| | | Obs. | 4,565 | 4,565 | 4,565 | 4,565 | 4,565 | 4,565 | 4,565 | 4,565 | 4,565 | 4,565 | 4,565 | 4,565 |
| | After Covid | Mean | 6.47 | 2.53 | 3.95 | 27.55 | 7.79 | 8.71 | 11.05 | 35.76 | 26.12 | 6.36 | 3.28 | 69.78 |
| | | St. dev. | 19.87 | 9.15 | 15.91 | 76.77 | 25.49 | 26.77 | 11.05 | 106.33 | 81.21 | 30.16 | 20.45 | 183.70 |
| | | Obs. | 2,739 | 2,739 | 2,739 | 2,739 | 2,739 | 2,739 | 2,739 | 2,739 | 2,739 | 2,739 | 2,739 | 2,739 |
| **Discounter** | All | Mean | 8.17 | 3.57 | 4.60 | 37.42 | 13.95 | 12.62 | 10.84 | 44.23 | 27.59 | 12.56 | 4.08 | 89.82 |
| | | St. dev. | 17.14 | 9.13 | 11.66 | 73.68 | 31.61 | 28.05 | 10.84 | 89.64 | 62.71 | 33.32 | 17.22 | 163.84 |
| | | Obs. | 7,304 | 7,304 | 7,304 | 7,304 | 7,304 | 7,304 | 7,304 | 7,304 | 7,304 | 7,304 | 7,304 | 7,304 |
| | Before Covid | Mean | 8.05 | 3.60 | 4.45 | 37.17 | 13.88 | 12.65 | 10.64 | 42.46 | 26.56 | 11.85 | 4.05 | 87.68 |
| | | St. dev. | 16.89 | 9.14 | 11.26 | 72.91 | 31.59 | 28.34 | 10.64 | 85.77 | 61.11 | 30.49 | 17.42 | 158.76 |
| | | Obs. | 4,565 | 4,565 | 4,565 | 4,565 | 4,565 | 4,565 | 4,565 | 4,565 | 4,565 | 4,565 | 4,565 | 4,565 |
| | After Covid | Mean | 8.37 | 3.53 | 4.85 | 37.82 | 14.07 | 12.57 | 11.18 | 47.19 | 29.32 | 13.75 | 4.13 | 93.39 |
| | | St. dev. | 17.57 | 9.11 | 12.30 | 74.95 | 31.67 | 27.57 | 11.18 | 95.69 | 65.27 | 37.55 | 16.90 | 171.95 |
| | | Obs. | 2,739 | 2,739 | 2,739 | 2,739 | 2,739 | 2,739 | 2,739 | 2,739 | 2,739 | 2,739 | 2,739 | 2,739 |
| Large store | All | Mean | 47.11 | 17.14 | 29.97 | 156.65 | 47.99 | 44.65 | 64.01 | 159.15 | 103.25 | 33.92 | 21.97 | 362.91 |
| | | St. dev. | 53.31 | 24.95 | 40.55 | 177.96 | 69.97 | 62.37 | 64.01 | 195.77 | 156.66 | 58.54 | 46.56 | 359.19 |
| | | Obs. | 7,304 | 7,304 | 7,304 | 7,304 | 7,304 | 7,304 | 7,304 | 7,304 | 7,304 | 7,304 | 7,304 | 7,304 |
| | Before Covid | Mean | 47.10 | 17.47 | 29.63 | 155.84 | 47.25 | 44.53 | 64.06 | 156.47 | 101.06 | 33.85 | 21.57 | 359.42 |
| | | St. dev. | 52.35 | 25.02 | 39.88 | 174.30 | 68.90 | 61.42 | 64.06 | 190.23 | 152.83 | 58.32 | 45.54 | 347.57 |
| | | Obs. | 4,565 | 4,565 | 4,565 | 4,565 | 4,565 | 4,565 | 4,565 | 4,565 | 4,565 | 4,565 | 4,565 | 4,565 |
| | After Covid | Mean | 47.13 | 16.60 | 30.53 | 158.00 | 49.23 | 44.85 | 63.92 | 163.60 | 106.91 | 34.04 | 22.64 | 368.72 |
| | | St. dev. | 54.89 | 24.82 | 41.63 | 183.92 | 71.71 | 63.93 | 63.92 | 204.64 | 162.80 | 58.93 | 48.23 | 377.76 |
| | | Obs. | 2,739 | 2,739 | 2,739 | 2,739 | 2,739 | 2,739 | 2,739 | 2,739 | 2,739 | 2,739 | 2,739 | 2,739 |

Continues

| Retailer type | Period | Statistic | Fruit and vegetable products (pence/per capita per week) | | | | | | | | | | | Total |
|---|---|---|---|---|---|---|---|---|---|---|---|---|---|---|
| | | | **Potatoes** | | | **Vegetables (excl. potatoes)** | | | | **Fruit** | | | | |
| | | | **Total** | Fresh | Processed | **Total** | Fresh green | Other fresh | Processed vegetables | **Total** | Fresh fruit | Processed fruit | Fruit juices | |
| **Online** | All | Mean | 8.71 | 3.23 | 5.48 | 31.81 | 9.19 | 9.02 | 13.60 | 29.52 | 17.49 | 7.32 | 4.71 | 70.04 |
| | | St. dev. | 26.61 | 12.38 | 18.41 | 106.96 | 34.88 | 35.28 | 13.60 | 97.82 | 64.48 | 37.76 | 20.09 | 212.01 |
| | | Obs. | 7,304 | 7,304 | 7,304 | 7,304 | 7,304 | 7,304 | 7,304 | 7,304 | 7,304 | 7,304 | 7,304 | 7,304 |
| | Before Covid | Mean | 6.80 | 2.52 | 4.28 | 23.76 | 6.56 | 6.67 | 10.53 | 21.17 | 12.48 | 5.07 | 3.62 | 51.73 |
| | | St. dev. | 22.26 | 10.21 | 15.76 | 83.25 | 24.86 | 27.92 | 10.53 | 72.50 | 49.49 | 26.22 | 16.46 | 161.86 |
| | | Obs. | 4,565 | 4,565 | 4,565 | 4,565 | 4,565 | 4,565 | 4,565 | 4,565 | 4,565 | 4,565 | 4,565 | 4,565 |
| | After Covid | Mean | 11.89 | 4.42 | 7.47 | 45.24 | 13.59 | 12.94 | 18.71 | 43.45 | 25.85 | 11.07 | 6.53 | 100.57 |
| | | St. dev. | 32.35 | 15.25 | 21.98 | 136.65 | 46.73 | 44.66 | 18.71 | 128.26 | 83.04 | 51.34 | 24.91 | 273.37 |
| | | Obs. | 2,739 | 2,739 | 2,739 | 2,739 | 2,739 | 2,739 | 2,739 | 2,739 | 2,739 | 2,739 | 2,739 | 2,739 |
| **Other retailer** | All | Mean | .24 | .04 | .20 | .62 | .21 | .18 | .23 | .97 | .38 | .17 | .43 | 1.83 |
| | | St. dev. | 3.50 | .53 | 3.38 | 5.82 | 2.72 | 2.34 | .23 | 9.79 | 4.91 | 2.72 | 6.84 | 15.44 |
| | | Obs. | 7,304 | 7,304 | 7,304 | 7,304 | 7,304 | 7,304 | 7,304 | 7,304 | 7,304 | 7,304 | 7,304 | 7,304 |
| | Before Covid | Mean | .24 | .03 | .21 | .54 | .18 | .13 | .22 | .92 | .27 | .17 | .49 | 1.70 |
| | | St. dev. | 3.84 | .43 | 3.76 | 5.08 | 2.49 | 1.39 | .22 | 8.91 | 3.34 | 2.74 | 7.34 | 14.40 |
| | | Obs. | 4,565 | 4,565 | 4,565 | 4,565 | 4,565 | 4,565 | 4,565 | 4,565 | 4,565 | 4,565 | 4,565 | 4,565 |
| | After Covid | Mean | .24 | .05 | .19 | .76 | .25 | .26 | .25 | 1.05 | .55 | .17 | .32 | 2.05 |
| | | St. dev. | 2.84 | .66 | 2.64 | 6.87 | 3.07 | 3.37 | .25 | 11.10 | 6.76 | 2.69 | 5.92 | 17.04 |
| | | Obs. | 2,739 | 2,739 | 2,739 | 2,739 | 2,739 | 2,739 | 2,739 | 2,739 | 2,739 | 2,739 | 2,739 | 2,739 |
| **All retailers** | All | Mean | 71.24 | 26.39 | 44.85 | 252.99 | 78.79 | 74.34 | 99.86 | 268.22 | 172.41 | 61.61 | 34.20 | 592.45 |
| | | St. dev. | 59.11 | 29.10 | 47.02 | 215.43 | 84.92 | 78.17 | 99.86 | 247.84 | 195.96 | 85.67 | 58.97 | 422.74 |
| | | Obs. | 7,304 | 7,304 | 7,304 | 7,304 | 7,304 | 7,304 | 7,304 | 7,304 | 7,304 | 7,304 | 7,304 | 7,304 |
| | Before Covid | Mean | 68.59 | 25.83 | 42.76 | 242.05 | 74.89 | 71.04 | 96.12 | 252.30 | 161.72 | 58.24 | 32.35 | 562.94 |
| | | St. dev. | 57.48 | 28.41 | 45.61 | 203.75 | 81.11 | 75.54 | 96.12 | 234.86 | 189.13 | 79.47 | 55.68 | 395.61 |
| | | Obs. | 4,565 | 4,565 | 4,565 | 4,565 | 4,565 | 4,565 | 4,565 | 4,565 | 4,565 | 4,565 | 4,565 | 4,565 |
| | After Covid | Mean | 75.66 | 27.34 | 48.32 | 271.24 | 85.30 | 79.85 | 106.09 | 294.75 | 190.22 | 67.25 | 37.28 | 641.64 |
| | | St. dev. | 61.49 | 30.21 | 49.09 | 232.49 | 90.56 | 82.07 | 106.09 | 266.02 | 205.65 | 94.86 | 63.97 | 460.34 |
| | | Obs. | 2,739 | 2,739 | 2,739 | 2,739 | 2,739 | 2,739 | 2,739 | 2,739 | 2,739 | 2,739 | 2,739 | 2,739 |

Source: Own elaboration based on Kantar Worldpanel data.

# Table A5. Midlands region - Average per capita purchases of fruit and vegetables before and after Covid-19 lockdown

| **Retailer type** | **Period** | **Statistic** | **Fruit and vegetable products (grams/per capita per week)** | | | | | | | | | | | **Total** |
|---|---|---|---|---|---|---|---|---|---|---|---|---|---|---|
| | | | **Potatoes** | | | **Vegetables (excl. potatoes)** | | | | **Fruit** | | | | |
| | | | **Total** | Fresh | Processed | **Total** | Fresh green | Other fresh | Processed vegetables | **Total** | Fresh fruit | Processed fruit | Fruit juices | |
| **Club and bargain store** | All | Mean | 13.22 | 4.93 | 8.29 | 15.98 | .58 | 1.05 | 14.35 | 12.74 | 5.50 | 4.60 | 2.64 | 41.94 |
| | | St. dev. | 57.76 | 41.99 | 34.91 | 57.20 | 5.37 | 11.79 | 14.35 | 63.99 | 42.01 | 24.01 | 31.98 | 130.01 |
| | | Obs. | 16,952 | 16,952 | 16,952 | 16,952 | 16,952 | 16,952 | 16,952 | 16,952 | 16,952 | 16,952 | 16,952 | 16,952 |
| | Before Covid | Mean | 13.06 | 5.13 | 7.93 | 15.96 | .57 | 1.04 | 14.35 | 12.77 | 5.80 | 4.61 | 2.36 | 41.79 |
| | | St. dev. | 58.51 | 43.60 | 33.74 | 58.00 | 5.17 | 11.71 | 14.35 | 65.27 | 44.62 | 24.27 | 30.04 | 130.33 |
| | | Obs. | 10,595 | 10,595 | 10,595 | 10,595 | 10,595 | 10,595 | 10,595 | 10,595 | 10,595 | 10,595 | 10,595 | 10,595 |
| | After Covid | Mean | 13.48 | 4.58 | 8.90 | 16.00 | .60 | 1.07 | 14.34 | 12.70 | 5.00 | 4.60 | 3.10 | 42.18 |
| | | St. dev. | 56.49 | 39.17 | 36.76 | 55.85 | 5.70 | 11.92 | 14.34 | 61.80 | 37.26 | 23.57 | 34.97 | 129.49 |
| | | Obs. | 6,357 | 6,357 | 6,357 | 6,357 | 6,357 | 6,357 | 6,357 | 6,357 | 6,357 | 6,357 | 6,357 | 6,357 |
| **Convenience** | All | Mean | 25.92 | 19.82 | 6.11 | 54.08 | 19.22 | 22.01 | 12.85 | 62.37 | 51.58 | 4.47 | 6.32 | 142.38 |
| | | St. dev. | 86.93 | 76.33 | 26.36 | 187.57 | 91.36 | 81.66 | 12.85 | 340.04 | 327.18 | 29.15 | 39.90 | 550.01 |
| | | Obs. | 16,952 | 16,952 | 16,952 | 16,952 | 16,952 | 16,952 | 16,952 | 16,952 | 16,952 | 16,952 | 16,952 | 16,952 |
| | Before Covid | Mean | 24.13 | 18.51 | 5.62 | 50.10 | 18.16 | 20.06 | 11.88 | 60.55 | 50.99 | 4.27 | 5.29 | 134.78 |
| | | St. dev. | 84.45 | 74.84 | 25.08 | 191.19 | 97.84 | 81.99 | 11.88 | 390.89 | 382.54 | 31.17 | 32.48 | 602.27 |
| | | Obs. | 10,595 | 10,595 | 10,595 | 10,595 | 10,595 | 10,595 | 10,595 | 10,595 | 10,595 | 10,595 | 10,595 | 10,595 |
| | After Covid | Mean | 28.91 | 21.99 | 6.92 | 60.71 | 20.99 | 25.25 | 14.47 | 65.42 | 52.57 | 4.82 | 8.03 | 155.04 |
| | | St. dev. | 90.84 | 78.72 | 28.35 | 181.19 | 79.37 | 81.00 | 14.47 | 231.66 | 203.88 | 25.42 | 49.82 | 449.35 |
| | | Obs. | 6,357 | 6,357 | 6,357 | 6,357 | 6,357 | 6,357 | 6,357 | 6,357 | 6,357 | 6,357 | 6,357 | 6,357 |
| **Discounter** | All | Mean | 165.22 | 115.89 | 49.33 | 330.81 | 102.03 | 108.86 | 119.93 | 298.72 | 198.42 | 32.52 | 67.78 | 794.75 |
| | | St. dev. | 298.02 | 234.42 | 108.33 | 539.42 | 191.73 | 199.48 | 119.93 | 526.04 | 361.82 | 87.25 | 233.51 | 1,209.47 |
| | | Obs. | 16,952 | 16,952 | 16,952 | 16,952 | 16,952 | 16,952 | 16,952 | 16,952 | 16,952 | 16,952 | 16,952 | 16,952 |
| | Before Covid | Mean | 162.62 | 114.07 | 48.55 | 327.76 | 100.77 | 107.50 | 119.49 | 297.77 | 200.37 | 31.34 | 66.06 | 788.15 |
| | | St. dev. | 290.42 | 230.59 | 105.56 | 523.09 | 185.31 | 194.01 | 119.49 | 517.95 | 357.34 | 83.54 | 233.14 | 1,178.57 |
| | | Obs. | 10,595 | 10,595 | 10,595 | 10,595 | 10,595 | 10,595 | 10,595 | 10,595 | 10,595 | 10,595 | 10,595 | 10,595 |
| | After Covid | Mean | 169.55 | 118.93 | 50.62 | 335.90 | 104.12 | 111.12 | 120.66 | 300.30 | 195.17 | 34.49 | 70.64 | 805.75 |
| | | St. dev. | 310.26 | 240.65 | 112.78 | 565.60 | 201.96 | 208.28 | 120.66 | 539.30 | 369.15 | 93.09 | 234.12 | 1,259.30 |
| | | Obs. | 6,357 | 6,357 | 6,357 | 6,357 | 6,357 | 6,357 | 6,357 | 6,357 | 6,357 | 6,357 | 6,357 | 6,357 |
| **Large store** | All | Mean | 333.25 | 219.19 | 114.06 | 598.43 | 170.19 | 185.10 | 243.15 | 547.00 | 366.65 | 52.70 | 127.65 | 1,478.69 |
| | | St. dev. | 381.67 | 305.86 | 161.83 | 636.79 | 240.17 | 238.86 | 243.15 | 678.71 | 515.81 | 103.95 | 265.23 | 1,429.04 |
| | | Obs. | 16,952 | 16,952 | 16,952 | 16,952 | 16,952 | 16,952 | 16,952 | 16,952 | 16,952 | 16,952 | 16,952 | 16,952 |
| | Before Covid | Mean | 327.71 | 216.71 | 110.99 | 597.95 | 166.86 | 182.99 | 248.10 | 544.78 | 369.01 | 51.66 | 124.11 | 1,470.43 |
| | | St. dev. | 369.34 | 297.48 | 155.13 | 626.66 | 233.38 | 233.29 | 248.10 | 664.77 | 510.93 | 101.97 | 258.72 | 1,387.67 |
| | | Obs. | 10,595 | 10,595 | 10,595 | 10,595 | 10,595 | 10,595 | 10,595 | 10,595 | 10,595 | 10,595 | 10,595 | 10,595 |
| | After Covid | Mean | 342.49 | 223.33 | 119.16 | 599.25 | 175.73 | 188.61 | 234.91 | 550.71 | 362.73 | 54.43 | 133.56 | 1,492.45 |
| | | St. dev. | 401.24 | 319.32 | 172.31 | 653.38 | 251.01 | 247.85 | 234.91 | 701.37 | 523.86 | 107.16 | 275.65 | 1,495.46 |
| | | Obs. | 6,357 | 6,357 | 6,357 | 6,357 | 6,357 | 6,357 | 6,357 | 6,357 | 6,357 | 6,357 | 6,357 | 6,357 |

Continues

| Retailer type | Period | Statistic | Fruit and vegetable products (grams/per capita per week) | | | | | | | | | | | Total |
|---|---|---|---|---|---|---|---|---|---|---|---|---|---|---|
| | | | Potatoes | | | Vegetables (excl. potatoes) | | | | Fruit | | | | |
| | | | **Total** | Fresh | Processed | **Total** | Fresh green | Other fresh | Processed vegetables | **Total** | Fresh fruit | Processed fruit | Fruit juices | |
| **Online** | All | Mean | 65.56 | 40.61 | 24.95 | 132.69 | 35.65 | 40.18 | 56.86 | 111.98 | 71.32 | 11.39 | 29.26 | 310.23 |
| | | St. dev. | 210.96 | 150.63 | 90.69 | 406.04 | 127.76 | 137.50 | 56.86 | 359.53 | 253.32 | 54.44 | 129.43 | 891.41 |
| | | Obs. | 16,952 | 16,952 | 16,952 | 16,952 | 16,952 | 16,952 | 16,952 | 16,952 | 16,952 | 16,952 | 16,952 | 16,952 |
| | Before Covid | Mean | 52.18 | 32.00 | 20.18 | 104.05 | 27.31 | 30.36 | 46.37 | 85.29 | 53.24 | 8.84 | 23.21 | 241.52 |
| | | St. dev. | 187.43 | 135.99 | 79.22 | 347.83 | 107.36 | 111.90 | 46.37 | 298.34 | 204.09 | 47.33 | 112.89 | 755.61 |
| | | Obs. | 10,595 | 10,595 | 10,595 | 10,595 | 10,595 | 10,595 | 10,595 | 10,595 | 10,595 | 10,595 | 10,595 | 10,595 |
| | After Covid | Mean | 87.85 | 54.96 | 32.90 | 180.43 | 49.54 | 56.54 | 74.35 | 156.45 | 101.44 | 15.66 | 39.35 | 424.74 |
| | | St. dev. | 243.60 | 171.33 | 106.63 | 484.13 | 154.95 | 170.65 | 74.35 | 439.56 | 316.66 | 64.35 | 152.55 | 1,070.76 |
| | | Obs. | 6,357 | 6,357 | 6,357 | 6,357 | 6,357 | 6,357 | 6,357 | 6,357 | 6,357 | 6,357 | 6,357 | 6,357 |
| **Other retailer** | All | Mean | 1.70 | 1.38 | .33 | 5.00 | 1.96 | 2.47 | .57 | 5.30 | 3.47 | .25 | 1.57 | 12.00 |
| | | St. dev. | 16.88 | 15.21 | 5.09 | 58.92 | 24.45 | 34.45 | .57 | 73.25 | 44.78 | 12.43 | 55.86 | 122.81 |
| | | Obs. | 16,952 | 16,952 | 16,952 | 16,952 | 16,952 | 16,952 | 16,952 | 16,952 | 16,952 | 16,952 | 16,952 | 16,952 |
| | Before Covid | Mean | 1.33 | .98 | .34 | 3.49 | 1.31 | 1.53 | .65 | 3.88 | 2.17 | .25 | 1.45 | 8.70 |
| | | St. dev. | 13.51 | 11.38 | 5.47 | 45.02 | 18.19 | 23.19 | .65 | 63.57 | 32.39 | 14.55 | 52.16 | 95.43 |
| | | Obs. | 10,595 | 10,595 | 10,595 | 10,595 | 10,595 | 10,595 | 10,595 | 10,595 | 10,595 | 10,595 | 10,595 | 10,595 |
| | After Covid | Mean | 2.33 | 2.03 | .30 | 7.52 | 3.03 | 4.04 | .45 | 7.66 | 5.63 | .26 | 1.77 | 17.50 |
| | | St. dev. | 21.34 | 20.01 | 4.38 | 76.62 | 32.27 | 47.59 | .45 | 86.97 | 59.93 | 7.69 | 61.54 | 158.10 |
| | | Obs. | 6,357 | 6,357 | 6,357 | 6,357 | 6,357 | 6,357 | 6,357 | 6,357 | 6,357 | 6,357 | 6,357 | 6,357 |
| **All retailers** | All | Mean | 604.87 | 401.81 | 203.06 | 1,136.99 | 329.62 | 359.66 | 447.71 | 1,038.11 | 696.94 | 105.95 | 235.22 | 2,779.97 |
| | | St. dev. | 457.30 | 385.94 | 201.99 | 777.81 | 318.97 | 309.42 | 447.71 | 885.35 | 707.51 | 160.82 | 380.66 | 1,642.82 |
| | | Obs. | 16,952 | 16,952 | 16,952 | 16,952 | 16,952 | 16,952 | 16,952 | 16,952 | 16,952 | 16,952 | 16,952 | 16,952 |
| | Before Covid | Mean | 581.02 | 387.41 | 193.61 | 1,099.30 | 314.99 | 343.48 | 440.84 | 1,005.03 | 681.58 | 100.97 | 222.48 | 2,685.36 |
| | | St. dev. | 444.28 | 378.12 | 192.48 | 755.58 | 307.07 | 298.62 | 440.84 | 889.88 | 725.30 | 158.06 | 369.20 | 1,615.01 |
| | | Obs. | 10,595 | 10,595 | 10,595 | 10,595 | 10,595 | 10,595 | 10,595 | 10,595 | 10,595 | 10,595 | 10,595 | 10,595 |
| | After Covid | Mean | 644.62 | 425.82 | 218.80 | 1,199.80 | 354.01 | 386.63 | 459.17 | 1,093.24 | 722.54 | 114.25 | 256.46 | 2,937.66 |
| | | St. dev. | 475.61 | 397.52 | 216.01 | 809.68 | 336.49 | 324.87 | 459.17 | 875.05 | 676.09 | 164.98 | 398.15 | 1,676.47 |
| | | Obs. | 6,357 | 6,357 | 6,357 | 6,357 | 6,357 | 6,357 | 6,357 | 6,357 | 6,357 | 6,357 | 6,357 | 6,357 |

Source: Own elaboration based on Kantar Worldpanel data.

## Table A6. Midlands region - Average per capita expenditure of fruit and vegetables before and after Covid-19 lockdown

| Retailer type | Period | Statistic | Fruit and vegetable products (pence/per capita per week) | | | | | | | | | | | Total |
|---|---|---|---|---|---|---|---|---|---|---|---|---|---|---|
| | | | Potatoes | | | Vegetables (excl. potatoes) | | | | Fruit | | | | |
| | | | **Total** | Fresh | Processed | **Total** | Fresh green | Other fresh | Processed vegetables | **Total** | Fresh fruit | Processed fruit | Fruit juices | |
| **Club and bargain store** | All | Mean | 2.78 | .28 | 2.50 | 2.55 | .15 | .23 | 2.17 | 3.35 | 1.02 | 2.11 | .22 | 8.67 |
| | | St. dev. | 9.58 | 2.38 | 9.00 | 8.84 | 1.72 | 2.74 | 2.17 | 16.85 | 9.42 | 11.23 | 2.29 | 25.85 |
| | | Obs. | 16,952 | 16,952 | 16,952 | 16,952 | 16,952 | 16,952 | 16,952 | 16,952 | 16,952 | 16,952 | 16,952 | 16,952 |
| | Before Covid | Mean | 2.76 | .29 | 2.46 | 2.47 | .14 | .21 | 2.12 | 3.35 | 1.04 | 2.10 | .21 | 8.58 |
| | | St. dev. | 9.75 | 2.54 | 9.11 | 8.32 | 1.38 | 2.38 | 2.12 | 16.78 | 9.61 | 10.68 | 2.30 | 25.18 |
| | | Obs. | 10,595 | 10,595 | 10,595 | 10,595 | 10,595 | 10,595 | 10,595 | 10,595 | 10,595 | 10,595 | 10,595 | 10,595 |
| | After Covid | Mean | 2.81 | .25 | 2.56 | 2.67 | .17 | .25 | 2.25 | 3.34 | .98 | 2.13 | .23 | 8.83 |
| | | St. dev. | 9.31 | 2.10 | 8.82 | 9.63 | 2.18 | 3.25 | 2.25 | 16.97 | 9.10 | 12.08 | 2.26 | 26.94 |
| | | Obs. | 6,357 | 6,357 | 6,357 | 6,357 | 6,357 | 6,357 | 6,357 | 6,357 | 6,357 | 6,357 | 6,357 | 6,357 |
| **Convenience** | All | Mean | 4.63 | 1.99 | 2.63 | 14.20 | 4.26 | 4.73 | 5.22 | 16.97 | 12.75 | 3.27 | .95 | 35.80 |
| | | St. dev. | 14.72 | 7.99 | 10.35 | 48.81 | 20.68 | 17.42 | 5.22 | 72.00 | 62.10 | 22.67 | 6.14 | 122.53 |
| | | Obs. | 16,952 | 16,952 | 16,952 | 16,952 | 16,952 | 16,952 | 16,952 | 16,952 | 16,952 | 16,952 | 16,952 | 16,952 |
| | Before Covid | Mean | 4.28 | 1.93 | 2.35 | 13.06 | 4.03 | 4.21 | 4.82 | 15.93 | 12.01 | 3.12 | .79 | 33.27 |
| | | St. dev. | 13.38 | 8.06 | 8.85 | 48.97 | 22.41 | 17.01 | 4.82 | 75.49 | 66.95 | 24.47 | 5.15 | 125.21 |
| | | Obs. | 10,595 | 10,595 | 10,595 | 10,595 | 10,595 | 10,595 | 10,595 | 10,595 | 10,595 | 10,595 | 10,595 | 10,595 |
| | After Covid | Mean | 5.19 | 2.09 | 3.10 | 16.11 | 4.63 | 5.60 | 5.88 | 18.72 | 13.98 | 3.52 | 1.21 | 40.02 |
| | | St. dev. | 16.69 | 7.88 | 12.43 | 48.49 | 17.39 | 18.06 | 5.88 | 65.74 | 53.03 | 19.28 | 7.50 | 117.80 |
| | | Obs. | 6,357 | 6,357 | 6,357 | 6,357 | 6,357 | 6,357 | 6,357 | 6,357 | 6,357 | 6,357 | 6,357 | 6,357 |
| **Discounter** | All | Mean | 15.56 | 6.88 | 8.68 | 50.64 | 16.37 | 17.53 | 16.73 | 55.50 | 37.47 | 12.89 | 5.15 | 121.69 |
| | | St. dev. | 27.03 | 13.80 | 18.20 | 85.89 | 32.41 | 33.15 | 16.73 | 94.35 | 69.03 | 33.60 | 18.21 | 186.51 |
| | | Obs. | 16,952 | 16,952 | 16,952 | 16,952 | 16,952 | 16,952 | 16,952 | 16,952 | 16,952 | 16,952 | 16,952 | 16,952 |
| | Before Covid | Mean | 15.40 | 6.92 | 8.48 | 50.62 | 16.41 | 17.58 | 16.63 | 54.47 | 36.96 | 12.54 | 4.96 | 120.49 |
| | | St. dev. | 26.13 | 13.84 | 17.28 | 84.64 | 31.98 | 32.64 | 16.63 | 90.60 | 66.73 | 32.39 | 18.22 | 180.92 |
| | | Obs. | 10,595 | 10,595 | 10,595 | 10,595 | 10,595 | 10,595 | 10,595 | 10,595 | 10,595 | 10,595 | 10,595 | 10,595 |
| | After Covid | Mean | 15.83 | 6.80 | 9.02 | 50.66 | 16.31 | 17.45 | 16.90 | 57.21 | 38.30 | 13.46 | 5.45 | 123.69 |
| | | St. dev. | 28.47 | 13.75 | 19.63 | 87.96 | 33.12 | 33.99 | 16.90 | 100.25 | 72.71 | 35.53 | 18.18 | 195.46 |
| | | Obs. | 6,357 | 6,357 | 6,357 | 6,357 | 6,357 | 6,357 | 6,357 | 6,357 | 6,357 | 6,357 | 6,357 | 6,357 |
| **Large store** | All | Mean | 47.38 | 16.34 | 31.04 | 120.42 | 32.87 | 35.39 | 52.17 | 120.76 | 81.78 | 25.16 | 13.82 | 288.56 |
| | | St. dev. | 53.10 | 23.37 | 40.99 | 137.65 | 48.81 | 50.92 | 52.17 | 167.06 | 128.57 | 51.40 | 30.79 | 305.51 |
| | | Obs. | 16,952 | 16,952 | 16,952 | 16,952 | 16,952 | 16,952 | 16,952 | 16,952 | 16,952 | 16,952 | 16,952 | 16,952 |
| | Before Covid | Mean | 46.97 | 16.80 | 30.17 | 120.71 | 32.82 | 35.24 | 52.65 | 119.93 | 81.92 | 24.57 | 13.44 | 287.62 |
| | | St. dev. | 51.71 | 23.67 | 39.37 | 135.02 | 48.31 | 49.57 | 52.65 | 163.83 | 126.63 | 50.39 | 30.15 | 297.49 |
| | | Obs. | 10,595 | 10,595 | 10,595 | 10,595 | 10,595 | 10,595 | 10,595 | 10,595 | 10,595 | 10,595 | 10,595 | 10,595 |
| | After Covid | Mean | 48.05 | 15.57 | 32.48 | 119.94 | 32.94 | 35.64 | 51.36 | 122.14 | 81.53 | 26.15 | 14.46 | 290.13 |
| | | St. dev. | 55.34 | 22.85 | 43.52 | 141.92 | 49.64 | 53.09 | 51.36 | 172.32 | 131.74 | 53.04 | 31.82 | 318.43 |
| | | Obs. | 6,357 | 6,357 | 6,357 | 6,357 | 6,357 | 6,357 | 6,357 | 6,357 | 6,357 | 6,357 | 6,357 | 6,357 |

Continues

| Retailer type | Period | Statistic | Fruit and vegetable products (pence/per capita per week) | | | | | | | | | | | Total |
|---|---|---|---|---|---|---|---|---|---|---|---|---|---|---|
| | | | **Potatoes** | | | **Vegetables (excl. potatoes)** | | | | **Fruit** | | | | |
| | | | **Total** | Fresh | Processed | **Total** | Fresh green | Other fresh | Processed vegetables | **Total** | Fresh fruit | Processed fruit | Fruit juices | |
| **Online** | All | Mean | 10.10 | 3.26 | 6.84 | 27.59 | 7.24 | 7.92 | 12.43 | 25.37 | 16.56 | 5.67 | 3.13 | 63.06 |
| | | St. dev. | 31.65 | 12.41 | 23.33 | 90.86 | 28.00 | 29.66 | 12.43 | 89.27 | 63.55 | 26.93 | 14.98 | 193.39 |
| | | Obs. | 16,952 | 16,952 | 16,952 | 16,952 | 16,952 | 16,952 | 16,952 | 16,952 | 16,952 | 16,952 | 16,952 | 16,952 |
| | Before Covid | Mean | 8.17 | 2.67 | 5.49 | 21.64 | 5.66 | 6.08 | 9.90 | 19.21 | 12.58 | 4.27 | 2.36 | 49.01 |
| | | St. dev. | 28.09 | 11.42 | 20.54 | 77.96 | 24.47 | 24.68 | 9.90 | 73.33 | 52.25 | 21.74 | 11.55 | 163.06 |
| | | Obs. | 10,595 | 10,595 | 10,595 | 10,595 | 10,595 | 10,595 | 10,595 | 10,595 | 10,595 | 10,595 | 10,595 | 10,595 |
| | After Covid | Mean | 13.32 | 4.24 | 9.08 | 37.51 | 9.88 | 11.00 | 16.63 | 35.64 | 23.21 | 8.01 | 4.41 | 86.47 |
| | | St. dev. | 36.60 | 13.84 | 27.22 | 108.29 | 32.89 | 36.28 | 16.63 | 110.10 | 78.43 | 33.73 | 19.32 | 233.56 |
| | | Obs. | 6,357 | 6,357 | 6,357 | 6,357 | 6,357 | 6,357 | 6,357 | 6,357 | 6,357 | 6,357 | 6,357 | 6,357 |
| **Other retailer** | All | Mean | .34 | .12 | .22 | .93 | .40 | .39 | .14 | .83 | .58 | .09 | .15 | 2.10 |
| | | St. dev. | 3.84 | 1.29 | 3.42 | 11.02 | 5.36 | 5.67 | .14 | 9.35 | 7.09 | 3.29 | 4.85 | 20.05 |
| | | Obs. | 16,952 | 16,952 | 16,952 | 16,952 | 16,952 | 16,952 | 16,952 | 16,952 | 16,952 | 16,952 | 16,952 | 16,952 |
| | Before Covid | Mean | .34 | .10 | .24 | .67 | .27 | .24 | .16 | .60 | .38 | .09 | .13 | 1.61 |
| | | St. dev. | 4.03 | 1.14 | 3.70 | 8.86 | 4.07 | 3.85 | .16 | 7.84 | 5.35 | 3.74 | 4.24 | 16.25 |
| | | Obs. | 10,595 | 10,595 | 10,595 | 10,595 | 10,595 | 10,595 | 10,595 | 10,595 | 10,595 | 10,595 | 10,595 | 10,595 |
| | After Covid | Mean | .35 | .15 | .20 | 1.36 | .62 | .63 | .11 | 1.19 | .91 | .10 | .18 | 2.91 |
| | | St. dev. | 3.51 | 1.49 | 2.88 | 13.87 | 6.99 | 7.81 | .11 | 11.42 | 9.28 | 2.36 | 5.72 | 25.13 |
| | | Obs. | 6,357 | 6,357 | 6,357 | 6,357 | 6,357 | 6,357 | 6,357 | 6,357 | 6,357 | 6,357 | 6,357 | 6,357 |
| **All retailers** | All | Mean | 80.79 | 28.87 | 51.92 | 216.33 | 61.29 | 66.19 | 88.85 | 222.77 | 150.16 | 49.20 | 23.41 | 519.88 |
| | | St. dev. | 60.88 | 28.60 | 49.76 | 169.07 | 64.81 | 64.67 | 88.85 | 211.71 | 165.01 | 75.85 | 39.89 | 358.12 |
| | | Obs. | 16,952 | 16,952 | 16,952 | 16,952 | 16,952 | 16,952 | 16,952 | 16,952 | 16,952 | 16,952 | 16,952 | 16,952 |
| | Before Covid | Mean | 77.92 | 28.72 | 49.20 | 209.17 | 59.33 | 63.56 | 86.28 | 213.48 | 144.90 | 46.69 | 21.89 | 500.58 |
| | | St. dev. | 58.58 | 28.75 | 47.05 | 163.20 | 63.68 | 61.85 | 86.28 | 207.40 | 162.51 | 74.98 | 37.81 | 347.14 |
| | | Obs. | 10,595 | 10,595 | 10,595 | 10,595 | 10,595 | 10,595 | 10,595 | 10,595 | 10,595 | 10,595 | 10,595 | 10,595 |
| | After Covid | Mean | 85.56 | 29.11 | 56.45 | 228.25 | 64.55 | 70.57 | 93.14 | 238.25 | 158.92 | 53.38 | 25.94 | 552.05 |
| | | St. dev. | 64.25 | 28.35 | 53.67 | 177.81 | 66.54 | 68.90 | 93.14 | 217.84 | 168.75 | 77.12 | 43.01 | 373.52 |
| | | Obs. | 6,357 | 6,357 | 6,357 | 6,357 | 6,357 | 6,357 | 6,357 | 6,357 | 6,357 | 6,357 | 6,357 | 6,357 |

Source: Own elaboration based on Kantar Worldpanel data.

## Table A7. North region - Average per capita purchases of fruit and vegetables before and after Covid-19 lockdown

| Retailer type | Period | Statistic | Fruit and vegetable products (grams/per capita per week): Potatoes: Total | Potatoes: Fresh | Potatoes: Processed | Vegetables (excl. potatoes): Total | Vegetables: Fresh green | Vegetables: Other fresh | Vegetables: Processed vegetables | Fruit: Total | Fruit: Fresh fruit | Fruit: Processed fruit | Fruit: Fruit juices | Total |
|---|---|---|---|---|---|---|---|---|---|---|---|---|---|---|
| **Club and bargain store** | All | Mean | 19.76 | 9.23 | 10.53 | 23.48 | 1.36 | 2.31 | 19.80 | 17.48 | 8.08 | 6.14 | 3.26 | 60.72 |
| | | St. dev. | 79.74 | 61.81 | 45.29 | 72.25 | 10.54 | 18.30 | 19.80 | 74.09 | 50.47 | 31.67 | 31.33 | 168.09 |
| | | Obs. | 27,240 | 27,240 | 27,240 | 27,240 | 27,240 | 27,240 | 27,240 | 27,240 | 27,240 | 27,240 | 27,240 | 27,240 |
| | Before Covid | Mean | 19.69 | 9.49 | 10.19 | 23.71 | 1.33 | 2.35 | 20.03 | 17.22 | 8.12 | 6.04 | 3.05 | 60.61 |
| | | St. dev. | 79.41 | 61.88 | 44.87 | 70.89 | 10.79 | 17.94 | 20.03 | 70.65 | 48.36 | 30.79 | 30.75 | 163.13 |
| | | Obs. | 17,025 | 17,025 | 17,025 | 17,025 | 17,025 | 17,025 | 17,025 | 17,025 | 17,025 | 17,025 | 17,025 | 17,025 |
| | After Covid | Mean | 19.88 | 8.79 | 11.10 | 23.10 | 1.42 | 2.25 | 19.43 | 17.91 | 8.01 | 6.29 | 3.61 | 60.90 |
| | | St. dev. | 80.30 | 61.69 | 45.98 | 74.46 | 10.09 | 18.89 | 19.43 | 79.51 | 53.82 | 33.09 | 32.27 | 176.05 |
| | | Obs. | 10,215 | 10,215 | 10,215 | 10,215 | 10,215 | 10,215 | 10,215 | 10,215 | 10,215 | 10,215 | 10,215 | 10,215 |
| **Convenience** | All | Mean | 27.40 | 21.46 | 5.95 | 50.80 | 17.40 | 20.57 | 12.83 | 55.16 | 44.54 | 3.85 | 6.78 | 133.37 |
| | | St. dev. | 97.58 | 87.51 | 27.15 | 159.88 | 68.52 | 75.65 | 12.83 | 191.68 | 169.04 | 23.54 | 48.96 | 390.25 |
| | | Obs. | 27,240 | 27,240 | 27,240 | 27,240 | 27,240 | 27,240 | 27,240 | 27,240 | 27,240 | 27,240 | 27,240 | 27,240 |
| | Before Covid | Mean | 26.21 | 20.77 | 5.44 | 46.94 | 16.27 | 18.53 | 12.14 | 51.73 | 42.07 | 3.65 | 6.00 | 124.88 |
| | | St. dev. | 96.87 | 88.51 | 24.68 | 149.67 | 64.70 | 69.86 | 12.14 | 180.97 | 160.74 | 23.21 | 44.07 | 368.67 |
| | | Obs. | 17,025 | 17,025 | 17,025 | 17,025 | 17,025 | 17,025 | 17,025 | 17,025 | 17,025 | 17,025 | 17,025 | 17,025 |
| | After Covid | Mean | 29.38 | 22.59 | 6.79 | 57.25 | 19.29 | 23.98 | 13.98 | 60.88 | 48.65 | 4.16 | 8.07 | 147.51 |
| | | St. dev. | 98.74 | 85.80 | 30.81 | 175.41 | 74.43 | 84.31 | 13.98 | 208.18 | 181.98 | 24.08 | 56.15 | 423.42 |
| | | Obs. | 10,215 | 10,215 | 10,215 | 10,215 | 10,215 | 10,215 | 10,215 | 10,215 | 10,215 | 10,215 | 10,215 | 10,215 |
| **Discounter** | All | Mean | 161.26 | 115.23 | 46.03 | 319.94 | 101.89 | 107.50 | 110.55 | 292.31 | 199.06 | 30.40 | 62.86 | 773.52 |
| | | St. dev. | 298.63 | 243.26 | 112.22 | 529.06 | 194.92 | 198.15 | 110.55 | 528.00 | 394.32 | 83.45 | 208.13 | 1,198.23 |
| | | Obs. | 27,240 | 27,240 | 27,240 | 27,240 | 27,240 | 27,240 | 27,240 | 27,240 | 27,240 | 27,240 | 27,240 | 27,240 |
| | Before Covid | Mean | 158.05 | 113.04 | 45.02 | 321.55 | 102.73 | 107.50 | 111.32 | 290.10 | 201.35 | 28.48 | 60.27 | 769.71 |
| | | St. dev. | 288.49 | 236.14 | 108.80 | 526.44 | 195.28 | 198.05 | 111.32 | 510.60 | 390.62 | 71.33 | 195.05 | 1,170.81 |
| | | Obs. | 17,025 | 17,025 | 17,025 | 17,025 | 17,025 | 17,025 | 17,025 | 17,025 | 17,025 | 17,025 | 17,025 | 17,025 |
| | After Covid | Mean | 166.61 | 118.89 | 47.73 | 317.25 | 100.49 | 107.49 | 109.27 | 296.00 | 195.24 | 33.58 | 67.18 | 779.86 |
| | | St. dev. | 314.75 | 254.66 | 117.68 | 533.42 | 194.33 | 198.32 | 109.27 | 555.80 | 400.40 | 100.38 | 228.23 | 1,242.61 |
| | | Obs. | 10,215 | 10,215 | 10,215 | 10,215 | 10,215 | 10,215 | 10,215 | 10,215 | 10,215 | 10,215 | 10,215 | 10,215 |
| **Large store** | All | Mean | 342.40 | 223.17 | 119.23 | 586.67 | 158.56 | 179.29 | 248.82 | 537.22 | 350.99 | 50.60 | 135.63 | 1,466.29 |
| | | St. dev. | 391.86 | 315.84 | 173.38 | 612.76 | 222.87 | 247.36 | 248.82 | 677.67 | 491.48 | 95.24 | 302.73 | 1,386.22 |
| | | Obs. | 27,240 | 27,240 | 27,240 | 27,240 | 27,240 | 27,240 | 27,240 | 27,240 | 27,240 | 27,240 | 27,240 | 27,240 |
| | Before Covid | Mean | 338.66 | 219.57 | 119.09 | 583.81 | 154.98 | 176.04 | 252.78 | 533.97 | 351.02 | 49.39 | 133.56 | 1,456.43 |
| | | St. dev. | 387.84 | 313.22 | 172.41 | 593.62 | 213.31 | 242.06 | 252.78 | 661.82 | 484.85 | 91.77 | 295.49 | 1,344.11 |
| | | Obs. | 17,025 | 17,025 | 17,025 | 17,025 | 17,025 | 17,025 | 17,025 | 17,025 | 17,025 | 17,025 | 17,025 | 17,025 |
| | After Covid | Mean | 348.63 | 229.18 | 119.45 | 591.44 | 164.52 | 184.69 | 242.23 | 542.65 | 350.95 | 52.61 | 139.09 | 1,482.72 |
| | | St. dev. | 398.41 | 320.09 | 175.00 | 643.39 | 237.85 | 255.86 | 242.23 | 703.30 | 502.37 | 100.73 | 314.40 | 1,453.62 |
| | | Obs. | 10,215 | 10,215 | 10,215 | 10,215 | 10,215 | 10,215 | 10,215 | 10,215 | 10,215 | 10,215 | 10,215 | 10,215 |

Continues

| Retailer type | Period | Statistic | Fruit and vegetable products (grams/per capita per week) | | | | | | | | | | | Total |
|---|---|---|---|---|---|---|---|---|---|---|---|---|---|---|
| | | | Potatoes | | | Vegetables (excl. potatoes) | | | | Fruit | | | | |
| | | | **Total** | Fresh | Processed | **Total** | Fresh green | Other fresh | Processed vegetables | **Total** | Fresh fruit | Processed fruit | Fruit juices | |
| **Online** | All | Mean | 56.43 | 36.32 | 20.11 | 104.21 | 28.58 | 30.31 | 45.32 | 84.69 | 54.46 | 9.15 | 21.08 | 245.33 |
| | | St. dev. | 211.98 | 156.51 | 88.86 | 375.76 | 129.50 | 124.05 | 45.32 | 329.15 | 231.35 | 49.11 | 124.44 | 836.33 |
| | | Obs. | 27,240 | 27,240 | 27,240 | 27,240 | 27,240 | 27,240 | 27,240 | 27,240 | 27,240 | 27,240 | 27,240 | 27,240 |
| | Before Covid | Mean | 40.85 | 24.92 | 15.94 | 75.87 | 19.67 | 21.03 | 35.18 | 58.67 | 37.65 | 6.02 | 15.00 | 175.39 |
| | | St. dev. | 172.67 | 121.69 | 79.00 | 301.85 | 100.39 | 96.64 | 35.18 | 253.02 | 180.18 | 36.38 | 99.09 | 658.25 |
| | | Obs. | 17,025 | 17,025 | 17,025 | 17,025 | 17,025 | 17,025 | 17,025 | 17,025 | 17,025 | 17,025 | 17,025 | 17,025 |
| | After Covid | Mean | 82.39 | 55.33 | 27.06 | 151.45 | 43.44 | 45.79 | 62.22 | 128.05 | 82.48 | 14.36 | 31.22 | 361.89 |
| | | St. dev. | 262.80 | 200.18 | 102.84 | 470.22 | 166.05 | 158.40 | 62.22 | 423.33 | 295.58 | 64.68 | 157.38 | 1,058.94 |
| | | Obs. | 10,215 | 10,215 | 10,215 | 10,215 | 10,215 | 10,215 | 10,215 | 10,215 | 10,215 | 10,215 | 10,215 | 10,215 |
| **Other retailer** | All | Mean | 1.56 | 1.29 | .27 | 3.77 | 1.60 | 1.49 | .68 | 2.87 | 2.21 | .17 | .49 | 8.19 |
| | | St. dev. | 18.45 | 17.90 | 3.53 | 52.31 | 30.43 | 22.07 | .68 | 40.35 | 38.55 | 3.47 | 8.65 | 102.58 |
| | | Obs. | 27,240 | 27,240 | 27,240 | 27,240 | 27,240 | 27,240 | 27,240 | 27,240 | 27,240 | 27,240 | 27,240 | 27,240 |
| | Before Covid | Mean | 1.24 | 1.00 | .24 | 2.71 | 1.13 | 1.06 | .52 | 2.03 | 1.54 | .15 | .34 | 5.98 |
| | | St. dev. | 15.14 | 14.73 | 3.24 | 37.30 | 20.38 | 17.11 | .52 | 30.30 | 29.08 | 3.53 | 6.55 | 75.25 |
| | | Obs. | 17,025 | 17,025 | 17,025 | 17,025 | 17,025 | 17,025 | 17,025 | 17,025 | 17,025 | 17,025 | 17,025 | 17,025 |
| | After Covid | Mean | 2.09 | 1.76 | .32 | 5.53 | 2.38 | 2.20 | .95 | 4.26 | 3.34 | .19 | .73 | 11.88 |
| | | St. dev. | 22.91 | 22.18 | 3.98 | 70.52 | 42.14 | 28.47 | .95 | 52.99 | 50.52 | 3.38 | 11.32 | 136.40 |
| | | Obs. | 10,215 | 10,215 | 10,215 | 10,215 | 10,215 | 10,215 | 10,215 | 10,215 | 10,215 | 10,215 | 10,215 | 10,215 |
| **All retailers** | All | Mean | 608.81 | 406.70 | 202.11 | 1,088.87 | 309.39 | 341.47 | 438.01 | 989.74 | 659.34 | 100.29 | 230.10 | 2,687.42 |
| | | St. dev. | 476.09 | 406.32 | 220.20 | 779.45 | 317.94 | 320.76 | 438.01 | 861.65 | 668.10 | 145.24 | 391.16 | 1,632.76 |
| | | Obs. | 27,240 | 27,240 | 27,240 | 27,240 | 27,240 | 27,240 | 27,240 | 27,240 | 27,240 | 27,240 | 27,240 | 27,240 |
| | Before Covid | Mean | 584.71 | 388.80 | 195.91 | 1,054.58 | 296.11 | 326.51 | 431.97 | 953.72 | 641.75 | 93.75 | 218.22 | 2,593.01 |
| | | St. dev. | 465.04 | 396.74 | 216.15 | 757.01 | 305.28 | 314.44 | 431.97 | 831.17 | 653.17 | 132.25 | 372.71 | 1,577.92 |
| | | Obs. | 17,025 | 17,025 | 17,025 | 17,025 | 17,025 | 17,025 | 17,025 | 17,025 | 17,025 | 17,025 | 17,025 | 17,025 |
| | After Covid | Mean | 648.99 | 436.54 | 212.45 | 1,146.01 | 331.53 | 366.41 | 448.08 | 1,049.76 | 688.66 | 111.19 | 249.91 | 2,844.76 |
| | | St. dev. | 491.36 | 420.15 | 226.43 | 812.30 | 336.84 | 329.54 | 448.08 | 907.05 | 691.31 | 164.06 | 419.39 | 1,708.80 |
| | | Obs. | 10,215 | 10,215 | 10,215 | 10,215 | 10,215 | 10,215 | 10,215 | 10,215 | 10,215 | 10,215 | 10,215 | 10,215 |

Source: Own elaboration based on Kantar Worldpanel data.

## Table A8. North region - Average per capita expenditure of fruit and vegetables before and after Covid-19 lockdown

| Retailer type | Period | Statistic | Fruit and vegetable products (pence/per capita per week) | | | | | | | | | | | Total |
|---|---|---|---|---|---|---|---|---|---|---|---|---|---|---|
| | | | Potatoes | | | Vegetables (excl. potatoes) | | | | Fruit | | | | |
| | | | **Total** | Fresh | Processed | **Total** | Fresh green | Other fresh | Processed vegetables | **Total** | Fresh fruit | Processed fruit | Fruit juices | |
| **Club and bargain store** | All | Mean | 3.79 | .51 | 3.28 | 4.12 | .30 | .52 | 3.31 | 4.35 | 1.46 | 2.60 | .28 | 12.26 |
| | | St. dev. | 11.51 | 3.51 | 10.63 | 14.84 | 2.64 | 5.69 | 3.31 | 17.87 | 10.76 | 11.86 | 2.73 | 32.91 |
| | | Obs. | 27,240 | 27,240 | 27,240 | 27,240 | 27,240 | 27,240 | 27,240 | 27,240 | 27,240 | 27,240 | 27,240 | 27,240 |
| | Before Covid | Mean | 3.73 | .51 | 3.22 | 4.05 | .29 | .51 | 3.25 | 4.24 | 1.42 | 2.54 | .28 | 12.01 |
| | | St. dev. | 11.30 | 3.38 | 10.46 | 14.19 | 2.57 | 5.97 | 3.25 | 16.62 | 9.77 | 11.41 | 2.90 | 31.27 |
| | | Obs. | 17,025 | 17,025 | 17,025 | 17,025 | 17,025 | 17,025 | 17,025 | 17,025 | 17,025 | 17,025 | 17,025 | 17,025 |
| | After Covid | Mean | 3.89 | .50 | 3.40 | 4.25 | .31 | .53 | 3.41 | 4.53 | 1.54 | 2.71 | .28 | 12.67 |
| | | St. dev. | 11.87 | 3.73 | 10.91 | 15.88 | 2.76 | 5.19 | 3.41 | 19.80 | 12.25 | 12.58 | 2.43 | 35.48 |
| | | Obs. | 10,215 | 10,215 | 10,215 | 10,215 | 10,215 | 10,215 | 10,215 | 10,215 | 10,215 | 10,215 | 10,215 | 10,215 |
| **Convenience** | All | Mean | 4.77 | 2.19 | 2.58 | 13.56 | 3.92 | 4.30 | 5.34 | 14.83 | 11.02 | 2.79 | 1.02 | 33.16 |
| | | St. dev. | 16.19 | 9.08 | 11.34 | 43.16 | 15.80 | 15.67 | 5.34 | 54.05 | 44.11 | 17.21 | 7.48 | 99.09 |
| | | Obs. | 27,240 | 27,240 | 27,240 | 27,240 | 27,240 | 27,240 | 27,240 | 27,240 | 27,240 | 27,240 | 27,240 | 27,240 |
| | Before Covid | Mean | 4.49 | 2.20 | 2.29 | 12.49 | 3.63 | 3.85 | 5.01 | 13.66 | 10.19 | 2.59 | .88 | 30.63 |
| | | St. dev. | 14.92 | 9.45 | 9.72 | 40.08 | 14.82 | 14.29 | 5.01 | 50.85 | 41.89 | 16.56 | 6.44 | 91.64 |
| | | Obs. | 17,025 | 17,025 | 17,025 | 17,025 | 17,025 | 17,025 | 17,025 | 17,025 | 17,025 | 17,025 | 17,025 | 17,025 |
| | After Covid | Mean | 5.24 | 2.17 | 3.07 | 15.36 | 4.42 | 5.07 | 5.88 | 16.77 | 12.40 | 3.12 | 1.25 | 37.37 |
| | | St. dev. | 18.11 | 8.42 | 13.61 | 47.80 | 17.30 | 17.71 | 5.88 | 58.96 | 47.54 | 18.23 | 8.95 | 110.27 |
| | | Obs. | 10,215 | 10,215 | 10,215 | 10,215 | 10,215 | 10,215 | 10,215 | 10,215 | 10,215 | 10,215 | 10,215 | 10,215 |
| **Discounter** | All | Mean | 15.10 | 6.98 | 8.12 | 50.09 | 16.12 | 17.46 | 16.51 | 55.24 | 38.36 | 11.98 | 4.90 | 120.43 |
| | | St. dev. | 26.56 | 14.26 | 17.58 | 83.36 | 31.35 | 32.52 | 16.51 | 101.82 | 79.68 | 30.13 | 16.78 | 190.21 |
| | | Obs. | 27,240 | 27,240 | 27,240 | 27,240 | 27,240 | 27,240 | 27,240 | 27,240 | 27,240 | 27,240 | 27,240 | 27,240 |
| | Before Covid | Mean | 14.80 | 6.98 | 7.82 | 50.82 | 16.50 | 17.72 | 16.61 | 54.27 | 38.03 | 11.57 | 4.67 | 119.90 |
| | | St. dev. | 25.51 | 13.90 | 16.83 | 83.78 | 32.05 | 32.75 | 16.61 | 97.65 | 76.79 | 29.01 | 15.68 | 185.86 |
| | | Obs. | 17,025 | 17,025 | 17,025 | 17,025 | 17,025 | 17,025 | 17,025 | 17,025 | 17,025 | 17,025 | 17,025 | 17,025 |
| | After Covid | Mean | 15.60 | 6.97 | 8.63 | 48.88 | 15.50 | 17.04 | 16.34 | 56.84 | 38.90 | 12.66 | 5.29 | 121.33 |
| | | St. dev. | 28.22 | 14.84 | 18.74 | 82.65 | 30.13 | 32.12 | 16.34 | 108.40 | 84.27 | 31.91 | 18.47 | 197.26 |
| | | Obs. | 10,215 | 10,215 | 10,215 | 10,215 | 10,215 | 10,215 | 10,215 | 10,215 | 10,215 | 10,215 | 10,215 | 10,215 |
| **Large store** | All | Mean | 47.86 | 16.51 | 31.35 | 118.27 | 30.61 | 33.11 | 54.55 | 113.01 | 75.04 | 23.45 | 14.52 | 279.14 |
| | | St. dev. | 52.87 | 24.30 | 40.75 | 132.12 | 46.03 | 44.78 | 54.55 | 144.12 | 109.45 | 43.47 | 32.46 | 275.15 |
| | | Obs. | 27,240 | 27,240 | 27,240 | 27,240 | 27,240 | 27,240 | 27,240 | 27,240 | 27,240 | 27,240 | 27,240 | 27,240 |
| | Before Covid | Mean | 47.80 | 16.82 | 30.98 | 118.22 | 30.57 | 32.81 | 54.84 | 111.28 | 74.26 | 22.80 | 14.21 | 277.30 |
| | | St. dev. | 52.20 | 24.79 | 39.72 | 128.46 | 45.33 | 43.36 | 54.84 | 138.84 | 106.35 | 41.38 | 30.92 | 265.58 |
| | | Obs. | 17,025 | 17,025 | 17,025 | 17,025 | 17,025 | 17,025 | 17,025 | 17,025 | 17,025 | 17,025 | 17,025 | 17,025 |
| | After Covid | Mean | 47.97 | 16.00 | 31.97 | 118.35 | 30.67 | 33.61 | 54.07 | 115.88 | 76.32 | 24.52 | 15.05 | 282.20 |
| | | St. dev. | 53.97 | 23.44 | 42.41 | 138.00 | 47.17 | 47.05 | 54.07 | 152.47 | 114.43 | 46.72 | 34.87 | 290.39 |
| | | Obs. | 10,215 | 10,215 | 10,215 | 10,215 | 10,215 | 10,215 | 10,215 | 10,215 | 10,215 | 10,215 | 10,215 | 10,215 |

Continues

| Retailer type | Period | Statistic | Fruit and vegetable products (pence/per capita per week) | | | | | | | | | | | Total |
|---|---|---|---|---|---|---|---|---|---|---|---|---|---|---|
| | | | Potatoes | | | Vegetables (excl. potatoes) | | | | Fruit | | | | |
| | | | **Total** | Fresh | Processed | **Total** | Fresh green | Other fresh | Processed vegetables | **Total** | Fresh fruit | Processed fruit | Fruit juices | |
| **Online** | All | Mean | 8.39 | 2.84 | 5.55 | 21.59 | 5.72 | 6.10 | 9.78 | 18.82 | 12.21 | 4.42 | 2.19 | 48.80 |
| | | St. dev. | 31.68 | 12.40 | 23.86 | 84.55 | 29.57 | 27.66 | 9.78 | 77.06 | 56.50 | 22.83 | 12.81 | 174.80 |
| | | Obs. | 27,240 | 27,240 | 27,240 | 27,240 | 27,240 | 27,240 | 27,240 | 27,240 | 27,240 | 27,240 | 27,240 | 27,240 |
| | Before Covid | Mean | 6.39 | 2.04 | 4.35 | 16.05 | 4.10 | 4.34 | 7.60 | 12.86 | 8.44 | 2.89 | 1.53 | 35.29 |
| | | St. dev. | 27.57 | 10.11 | 21.27 | 70.73 | 24.52 | 22.54 | 7.60 | 59.26 | 44.57 | 16.97 | 10.10 | 140.65 |
| | | Obs. | 17,025 | 17,025 | 17,025 | 17,025 | 17,025 | 17,025 | 17,025 | 17,025 | 17,025 | 17,025 | 17,025 | 17,025 |
| | After Covid | Mean | 11.72 | 4.17 | 7.55 | 30.84 | 8.42 | 9.02 | 13.40 | 28.74 | 18.50 | 6.96 | 3.28 | 71.31 |
| | | St. dev. | 37.31 | 15.39 | 27.53 | 102.90 | 36.31 | 34.33 | 13.40 | 99.13 | 71.67 | 30.00 | 16.29 | 218.41 |
| | | Obs. | 10,215 | 10,215 | 10,215 | 10,215 | 10,215 | 10,215 | 10,215 | 10,215 | 10,215 | 10,215 | 10,215 | 10,215 |
| **Other retailer** | All | Mean | .26 | .11 | .15 | .71 | .30 | .24 | .17 | .53 | .37 | .10 | .07 | 1.51 |
| | | St. dev. | 2.51 | 1.50 | 1.93 | 9.78 | 5.97 | 3.52 | .17 | 7.13 | 6.53 | 2.01 | 1.23 | 17.39 |
| | | Obs. | 27,240 | 27,240 | 27,240 | 27,240 | 27,240 | 27,240 | 27,240 | 27,240 | 27,240 | 27,240 | 27,240 | 27,240 |
| | Before Covid | Mean | .23 | .09 | .14 | .53 | .22 | .17 | .14 | .38 | .24 | .09 | .05 | 1.14 |
| | | St. dev. | 2.15 | 1.31 | 1.69 | 7.12 | 4.10 | 2.63 | .14 | 5.19 | 4.49 | 2.16 | .98 | 12.39 |
| | | Obs. | 17,025 | 17,025 | 17,025 | 17,025 | 17,025 | 17,025 | 17,025 | 17,025 | 17,025 | 17,025 | 17,025 | 17,025 |
| | After Covid | Mean | .32 | .14 | .18 | 1.02 | .45 | .36 | .21 | .79 | .59 | .10 | .10 | 2.13 |
| | | St. dev. | 3.02 | 1.77 | 2.28 | 13.06 | 8.17 | 4.63 | .21 | 9.52 | 8.95 | 1.75 | 1.57 | 23.44 |
| | | Obs. | 10,215 | 10,215 | 10,215 | 10,215 | 10,215 | 10,215 | 10,215 | 10,215 | 10,215 | 10,215 | 10,215 | 10,215 |
| **All retailers** | All | Mean | 80.17 | 29.13 | 51.04 | 208.36 | 56.98 | 61.73 | 89.65 | 206.77 | 138.46 | 45.33 | 22.98 | 495.30 |
| | | St. dev. | 62.17 | 30.05 | 50.57 | 166.54 | 64.55 | 60.01 | 89.65 | 189.81 | 150.29 | 62.90 | 39.70 | 332.98 |
| | | Obs. | 27,240 | 27,240 | 27,240 | 27,240 | 27,240 | 27,240 | 27,240 | 27,240 | 27,240 | 27,240 | 27,240 | 27,240 |
| | Before Covid | Mean | 77.43 | 28.64 | 48.79 | 202.15 | 55.30 | 59.39 | 87.46 | 196.70 | 132.59 | 42.48 | 21.62 | 476.27 |
| | | St. dev. | 60.48 | 30.02 | 48.71 | 161.09 | 62.63 | 58.10 | 87.46 | 180.01 | 143.47 | 58.79 | 37.06 | 317.74 |
| | | Obs. | 17,025 | 17,025 | 17,025 | 17,025 | 17,025 | 17,025 | 17,025 | 17,025 | 17,025 | 17,025 | 17,025 | 17,025 |
| | After Covid | Mean | 84.74 | 29.94 | 54.80 | 218.71 | 59.76 | 65.63 | 93.31 | 223.56 | 148.25 | 50.07 | 25.24 | 527.00 |
| | | St. dev. | 64.63 | 30.09 | 53.32 | 174.75 | 67.55 | 62.88 | 93.31 | 204.01 | 160.54 | 68.95 | 43.65 | 354.69 |
| | | Obs. | 10,215 | 10,215 | 10,215 | 10,215 | 10,215 | 10,215 | 10,215 | 10,215 | 10,215 | 10,215 | 10,215 | 10,215 |

Source: Own elaboration based on Kantar Worldpanel data.

## Table A9. South region - Average per capita purchases of fruit and vegetables before and after Covid-19 lockdown

| Retailer type | Period | Statistic | Fruit and vegetable products (grams/per capita per week) | | | | | | | | | | | Total |
|---|---|---|---|---|---|---|---|---|---|---|---|---|---|---|
| | | | Potatoes | | | Vegetables (excl. potatoes) | | | | Fruit | | | | |
| | | | **Total** | Fresh | Processed | **Total** | Fresh green | Other fresh | Processed vegetables | **Total** | Fresh fruit | Processed fruit | Fruit juices | |
| **Club and bargain store** | All | Mean | 6.82 | 2.95 | 3.87 | 8.83 | .48 | .92 | 7.43 | 7.87 | 3.28 | 3.12 | 1.47 | 23.53 |
| | | St. dev. | 40.07 | 29.27 | 24.20 | 40.65 | 6.46 | 10.39 | 7.43 | 43.89 | 27.34 | 19.64 | 22.76 | 91.44 |
| | | Obs. | 23,760 | 23,760 | 23,760 | 23,760 | 23,760 | 23,760 | 23,760 | 23,760 | 23,760 | 23,760 | 23,760 | 23,760 |
| | Before Covid | Mean | 6.89 | 2.92 | 3.97 | 8.87 | .44 | .89 | 7.55 | 7.78 | 3.28 | 3.28 | 1.22 | 23.54 |
| | | St. dev. | 39.92 | 28.08 | 26.06 | 40.25 | 6.31 | 9.18 | 7.55 | 43.51 | 26.43 | 21.82 | 22.19 | 88.23 |
| | | Obs. | 14,850 | 14,850 | 14,850 | 14,850 | 14,850 | 14,850 | 14,850 | 14,850 | 14,850 | 14,850 | 14,850 | 14,850 |
| | After Covid | Mean | 6.71 | 3.01 | 3.70 | 8.77 | .55 | .97 | 7.25 | 8.03 | 3.28 | 2.86 | 1.88 | 23.51 |
| | | St. dev. | 40.32 | 31.14 | 20.72 | 41.29 | 6.69 | 12.13 | 7.25 | 44.52 | 28.80 | 15.34 | 23.67 | 96.55 |
| | | Obs. | 8,910 | 8,910 | 8,910 | 8,910 | 8,910 | 8,910 | 8,910 | 8,910 | 8,910 | 8,910 | 8,910 | 8,910 |
| **Convenience** | All | Mean | 26.66 | 20.34 | 6.33 | 58.85 | 20.41 | 24.09 | 14.34 | 64.66 | 50.42 | 4.98 | 9.26 | 150.17 |
| | | St. dev. | 88.66 | 77.59 | 26.21 | 179.79 | 78.77 | 82.43 | 14.34 | 215.15 | 180.31 | 25.71 | 78.33 | 418.75 |
| | | Obs. | 23,760 | 23,760 | 23,760 | 23,760 | 23,760 | 23,760 | 23,760 | 23,760 | 23,760 | 23,760 | 23,760 | 23,760 |
| | Before Covid | Mean | 24.05 | 18.26 | 5.79 | 52.18 | 17.96 | 21.26 | 12.96 | 59.13 | 45.81 | 4.59 | 8.73 | 135.36 |
| | | St. dev. | 81.10 | 71.53 | 23.87 | 164.53 | 71.89 | 77.02 | 12.96 | 203.80 | 169.85 | 23.77 | 78.92 | 387.09 |
| | | Obs. | 14,850 | 14,850 | 14,850 | 14,850 | 14,850 | 14,850 | 14,850 | 14,850 | 14,850 | 14,850 | 14,850 | 14,850 |
| | After Covid | Mean | 31.01 | 23.80 | 7.21 | 69.97 | 24.51 | 28.81 | 16.65 | 73.87 | 58.09 | 5.63 | 10.15 | 174.85 |
| | | St. dev. | 99.85 | 86.65 | 29.68 | 202.21 | 88.93 | 90.53 | 16.65 | 232.57 | 196.27 | 28.64 | 77.33 | 465.75 |
| | | Obs. | 8,910 | 8,910 | 8,910 | 8,910 | 8,910 | 8,910 | 8,910 | 8,910 | 8,910 | 8,910 | 8,910 | 8,910 |
| **Discounter** | All | Mean | 117.61 | 81.27 | 36.34 | 269.07 | 88.18 | 91.35 | 89.54 | 240.06 | 161.15 | 27.36 | 51.56 | 626.75 |
| | | St. dev. | 229.91 | 177.35 | 89.44 | 503.44 | 187.14 | 195.72 | 89.54 | 468.48 | 345.01 | 68.30 | 172.71 | 1,072.51 |
| | | Obs. | 23,760 | 23,760 | 23,760 | 23,760 | 23,760 | 23,760 | 23,760 | 23,760 | 23,760 | 23,760 | 23,760 | 23,760 |
| | Before Covid | Mean | 117.17 | 81.13 | 36.04 | 270.94 | 89.10 | 91.94 | 89.90 | 239.94 | 164.59 | 25.99 | 49.36 | 628.04 |
| | | St. dev. | 223.61 | 173.84 | 86.76 | 491.74 | 183.69 | 187.43 | 89.90 | 456.51 | 342.06 | 64.38 | 167.68 | 1,041.20 |
| | | Obs. | 14,850 | 14,850 | 14,850 | 14,850 | 14,850 | 14,850 | 14,850 | 14,850 | 14,850 | 14,850 | 14,850 | 14,850 |
| | After Covid | Mean | 118.35 | 81.51 | 36.84 | 265.97 | 86.64 | 90.38 | 88.96 | 240.27 | 155.43 | 29.64 | 55.21 | 624.60 |
| | | St. dev. | 240.04 | 183.06 | 93.74 | 522.37 | 192.74 | 208.81 | 88.96 | 487.81 | 349.81 | 74.33 | 180.72 | 1,122.83 |
| | | Obs. | 8,910 | 8,910 | 8,910 | 8,910 | 8,910 | 8,910 | 8,910 | 8,910 | 8,910 | 8,910 | 8,910 | 8,910 |
| **Large store** | All | Mean | 343.94 | 230.28 | 113.66 | 679.90 | 202.70 | 211.33 | 265.87 | 634.35 | 419.72 | 63.03 | 151.60 | 1,658.19 |
| | | St. dev. | 392.72 | 318.34 | 156.14 | 734.95 | 287.95 | 260.00 | 265.87 | 738.93 | 543.51 | 110.89 | 332.46 | 1,534.97 |
| | | Obs. | 23,760 | 23,760 | 23,760 | 23,760 | 23,760 | 23,760 | 23,760 | 23,760 | 23,760 | 23,760 | 23,760 | 23,760 |
| | Before Covid | Mean | 343.89 | 231.23 | 112.65 | 686.70 | 203.95 | 212.03 | 270.73 | 643.13 | 431.05 | 61.77 | 150.30 | 1,673.71 |
| | | St. dev. | 385.96 | 317.55 | 151.76 | 720.15 | 284.32 | 255.54 | 270.73 | 735.62 | 550.40 | 106.23 | 325.41 | 1,505.15 |
| | | Obs. | 14,850 | 14,850 | 14,850 | 14,850 | 14,850 | 14,850 | 14,850 | 14,850 | 14,850 | 14,850 | 14,850 | 14,850 |
| | After Covid | Mean | 344.04 | 228.69 | 115.35 | 668.56 | 200.62 | 210.18 | 257.76 | 619.71 | 400.83 | 65.14 | 153.75 | 1,632.32 |
| | | St. dev. | 403.74 | 319.67 | 163.17 | 758.88 | 293.90 | 267.28 | 257.76 | 744.23 | 531.32 | 118.22 | 343.90 | 1,583.19 |
| | | Obs. | 8,910 | 8,910 | 8,910 | 8,910 | 8,910 | 8,910 | 8,910 | 8,910 | 8,910 | 8,910 | 8,910 | 8,910 |

Continues

| Retailer type | Period | Statistic | Fruit and vegetable products (grams/per capita per week) | | | | | | | | | | | Total |
|---|---|---|---|---|---|---|---|---|---|---|---|---|---|---|
| | | | Potatoes | | | Vegetables (excl. potatoes) | | | | Fruit | | | | |
| | | | **Total** | Fresh | Processed | **Total** | Fresh green | Other fresh | Processed vegetables | **Total** | Fresh fruit | Processed fruit | Fruit juices | |
| **Online** | All | Mean | 81.16 | 52.56 | 28.60 | 167.74 | 47.70 | 50.42 | 69.62 | 143.51 | 90.89 | 16.46 | 36.16 | 392.41 |
| | | St. dev. | 230.97 | 173.50 | 92.34 | 443.87 | 150.53 | 150.48 | 69.62 | 423.06 | 290.24 | 65.74 | 161.63 | 1,002.64 |
| | | Obs. | 23,760 | 23,760 | 23,760 | 23,760 | 23,760 | 23,760 | 23,760 | 23,760 | 23,760 | 23,760 | 23,760 | 23,760 |
| | Before Covid | Mean | 60.06 | 37.76 | 22.31 | 127.76 | 35.03 | 36.55 | 56.18 | 106.48 | 66.03 | 11.76 | 28.69 | 294.30 |
| | | St. dev. | 183.88 | 131.87 | 81.94 | 367.94 | 120.60 | 118.35 | 56.18 | 348.67 | 233.64 | 51.07 | 142.87 | 822.16 |
| | | Obs. | 14,850 | 14,850 | 14,850 | 14,850 | 14,850 | 14,850 | 14,850 | 14,850 | 14,850 | 14,850 | 14,850 | 14,850 |
| | After Covid | Mean | 116.32 | 77.23 | 39.09 | 234.38 | 68.82 | 73.54 | 92.02 | 205.23 | 132.33 | 24.30 | 48.60 | 555.93 |
| | | St. dev. | 289.71 | 224.32 | 106.65 | 540.99 | 188.34 | 190.24 | 92.02 | 518.26 | 361.83 | 84.16 | 188.14 | 1,229.45 |
| | | Obs. | 8,910 | 8,910 | 8,910 | 8,910 | 8,910 | 8,910 | 8,910 | 8,910 | 8,910 | 8,910 | 8,910 | 8,910 |
| **Other retailer** | All | Mean | 1.08 | .83 | .25 | 2.75 | 1.05 | 1.24 | .45 | 2.43 | 1.76 | .18 | .48 | 6.25 |
| | | St. dev. | 11.78 | 10.64 | 4.11 | 28.89 | 14.01 | 14.48 | .45 | 27.62 | 22.70 | 3.87 | 13.21 | 56.52 |
| | | Obs. | 23,760 | 23,760 | 23,760 | 23,760 | 23,760 | 23,760 | 23,760 | 23,760 | 23,760 | 23,760 | 23,760 | 23,760 |
| | Before Covid | Mean | .86 | .62 | .24 | 1.96 | .72 | .83 | .41 | 1.74 | 1.08 | .17 | .49 | 4.56 |
| | | St. dev. | 10.18 | 8.91 | 4.38 | 21.33 | 9.82 | 10.48 | .41 | 20.41 | 12.66 | 3.58 | 14.08 | 39.18 |
| | | Obs. | 14,850 | 14,850 | 14,850 | 14,850 | 14,850 | 14,850 | 14,850 | 14,850 | 14,850 | 14,850 | 14,850 | 14,850 |
| | After Covid | Mean | 1.44 | 1.18 | .26 | 4.06 | 1.61 | 1.93 | .52 | 3.57 | 2.90 | .21 | .46 | 9.07 |
| | | St. dev. | 14.04 | 13.03 | 3.61 | 38.27 | 19.04 | 19.38 | .52 | 36.58 | 33.24 | 4.30 | 11.63 | 77.13 |
| | | Obs. | 8,910 | 8,910 | 8,910 | 8,910 | 8,910 | 8,910 | 8,910 | 8,910 | 8,910 | 8,910 | 8,910 | 8,910 |
| **All retailers** | All | Mean | 577.28 | 388.23 | 189.05 | 1,187.14 | 360.52 | 379.37 | 447.26 | 1,092.88 | 727.22 | 115.14 | 250.52 | 2,857.30 |
| | | St. dev. | 439.39 | 371.92 | 190.25 | 841.30 | 354.43 | 323.79 | 447.26 | 880.86 | 679.45 | 147.59 | 412.94 | 1,638.21 |
| | | Obs. | 23,760 | 23,760 | 23,760 | 23,760 | 23,760 | 23,760 | 23,760 | 23,760 | 23,760 | 23,760 | 23,760 | 23,760 |
| | Before Covid | Mean | 552.92 | 371.92 | 181.00 | 1,148.40 | 347.18 | 363.51 | 437.72 | 1,058.19 | 711.84 | 107.55 | 238.80 | 2,759.51 |
| | | St. dev. | 422.69 | 360.00 | 183.30 | 808.47 | 342.15 | 307.90 | 437.72 | 860.66 | 672.02 | 136.61 | 398.71 | 1,575.10 |
| | | Obs. | 14,850 | 14,850 | 14,850 | 14,850 | 14,850 | 14,850 | 14,850 | 14,850 | 14,850 | 14,850 | 14,850 | 14,850 |
| | After Covid | Mean | 617.88 | 415.43 | 202.45 | 1,251.71 | 382.75 | 405.80 | 463.16 | 1,150.69 | 752.85 | 127.79 | 270.05 | 3,020.28 |
| | | St. dev. | 463.08 | 389.48 | 200.61 | 889.65 | 372.98 | 347.07 | 463.16 | 910.66 | 690.93 | 163.49 | 434.95 | 1,726.14 |
| | | Obs. | 8,910 | 8,910 | 8,910 | 8,910 | 8,910 | 8,910 | 8,910 | 8,910 | 8,910 | 8,910 | 8,910 | 8,910 |

Source: Own elaboration based on Kantar Worldpanel data.

# Table A10. South region - Average per capita expenditure of fruit and vegetables before and after Covid-19 lockdown

| Retailer type | Period | Statistic | Fruit and vegetable products (pence/per capita per week): Potatoes: Total | Potatoes: Fresh | Potatoes: Processed | Vegetables (excl. potatoes): Total | Vegetables: Fresh green | Vegetables: Other fresh | Vegetables: Processed vegetables | Fruit: Total | Fruit: Fresh fruit | Fruit: Processed fruit | Fruit: Fruit juices | Total |
|---|---|---|---|---|---|---|---|---|---|---|---|---|---|---|
| **Club and bargain store** | All | Mean | 1.48 | .17 | 1.31 | 1.61 | .13 | .23 | 1.25 | 2.27 | .66 | 1.47 | .14 | 5.36 |
| | | St. dev. | 6.73 | 1.63 | 6.40 | 7.44 | 1.75 | 2.51 | 1.25 | 11.87 | 6.47 | 8.46 | 2.04 | 19.17 |
| | | Obs. | 23,760 | 23,760 | 23,760 | 23,760 | 23,760 | 23,760 | 23,760 | 23,760 | 23,760 | 23,760 | 23,760 | 23,760 |
| | Before Covid | Mean | 1.48 | .17 | 1.32 | 1.57 | .12 | .23 | 1.22 | 2.24 | .64 | 1.48 | .11 | 5.29 |
| | | St. dev. | 6.84 | 1.59 | 6.56 | 6.81 | 1.72 | 2.32 | 1.22 | 11.55 | 6.50 | 8.45 | 1.78 | 18.03 |
| | | Obs. | 14,850 | 14,850 | 14,850 | 14,850 | 14,850 | 14,850 | 14,850 | 14,850 | 14,850 | 14,850 | 14,850 | 14,850 |
| | After Covid | Mean | 1.48 | .18 | 1.31 | 1.69 | .15 | .23 | 1.30 | 2.32 | .68 | 1.47 | .17 | 5.48 |
| | | St. dev. | 6.52 | 1.70 | 6.12 | 8.39 | 1.79 | 2.81 | 1.30 | 12.39 | 6.41 | 8.47 | 2.42 | 20.92 |
| | | Obs. | 8,910 | 8,910 | 8,910 | 8,910 | 8,910 | 8,910 | 8,910 | 8,910 | 8,910 | 8,910 | 8,910 | 8,910 |
| **Convenience** | All | Mean | 4.82 | 2.02 | 2.81 | 16.39 | 4.98 | 5.40 | 6.02 | 18.53 | 13.39 | 3.74 | 1.40 | 39.75 |
| | | St. dev. | 15.86 | 7.78 | 11.98 | 49.65 | 19.15 | 18.25 | 6.02 | 62.95 | 48.75 | 19.25 | 11.32 | 111.24 |
| | | Obs. | 23,760 | 23,760 | 23,760 | 23,760 | 23,760 | 23,760 | 23,760 | 23,760 | 23,760 | 23,760 | 23,760 | 23,760 |
| | Before Covid | Mean | 4.33 | 1.85 | 2.48 | 14.49 | 4.39 | 4.71 | 5.39 | 16.45 | 11.74 | 3.43 | 1.28 | 35.27 |
| | | St. dev. | 13.90 | 7.35 | 10.10 | 44.92 | 17.74 | 16.64 | 5.39 | 56.51 | 43.54 | 17.51 | 10.70 | 99.78 |
| | | Obs. | 14,850 | 14,850 | 14,850 | 14,850 | 14,850 | 14,850 | 14,850 | 14,850 | 14,850 | 14,850 | 14,850 | 14,850 |
| | After Covid | Mean | 5.64 | 2.29 | 3.34 | 19.57 | 5.97 | 6.54 | 7.05 | 22.00 | 16.16 | 4.24 | 1.60 | 47.21 |
| | | St. dev. | 18.65 | 8.43 | 14.58 | 56.53 | 21.25 | 20.60 | 7.05 | 72.31 | 56.28 | 21.82 | 12.29 | 127.75 |
| | | Obs. | 8,910 | 8,910 | 8,910 | 8,910 | 8,910 | 8,910 | 8,910 | 8,910 | 8,910 | 8,910 | 8,910 | 8,910 |
| **Discounter** | All | Mean | 11.71 | 5.01 | 6.69 | 42.92 | 14.66 | 15.18 | 13.09 | 47.16 | 31.13 | 12.05 | 3.98 | 101.79 |
| | | St. dev. | 22.51 | 10.78 | 15.64 | 80.66 | 31.52 | 31.74 | 13.09 | 92.38 | 67.96 | 31.30 | 13.49 | 174.72 |
| | | Obs. | 23,760 | 23,760 | 23,760 | 23,760 | 23,760 | 23,760 | 23,760 | 23,760 | 23,760 | 23,760 | 23,760 | 23,760 |
| | Before Covid | Mean | 11.67 | 5.10 | 6.57 | 43.52 | 14.92 | 15.57 | 13.03 | 46.58 | 31.08 | 11.74 | 3.75 | 101.76 |
| | | St. dev. | 22.09 | 10.76 | 15.37 | 79.75 | 31.07 | 31.84 | 13.03 | 88.46 | 65.88 | 30.22 | 12.83 | 169.32 |
| | | Obs. | 14,850 | 14,850 | 14,850 | 14,850 | 14,850 | 14,850 | 14,850 | 14,850 | 14,850 | 14,850 | 14,850 | 14,850 |
| | After Covid | Mean | 11.77 | 4.87 | 6.90 | 41.92 | 14.21 | 14.53 | 13.18 | 48.13 | 31.22 | 12.56 | 4.36 | 101.83 |
| | | St. dev. | 23.19 | 10.81 | 16.06 | 82.14 | 32.24 | 31.56 | 13.18 | 98.56 | 71.30 | 33.02 | 14.53 | 183.37 |
| | | Obs. | 8,910 | 8,910 | 8,910 | 8,910 | 8,910 | 8,910 | 8,910 | 8,910 | 8,910 | 8,910 | 8,910 | 8,910 |
| **Large store** | All | Mean | 48.51 | 17.67 | 30.84 | 144.25 | 42.17 | 42.04 | 60.04 | 144.76 | 95.22 | 32.14 | 17.41 | 337.53 |
| | | St. dev. | 52.73 | 24.54 | 39.76 | 164.75 | 64.32 | 57.73 | 60.04 | 179.29 | 131.17 | 59.57 | 41.40 | 334.39 |
| | | Obs. | 23,760 | 23,760 | 23,760 | 23,760 | 23,760 | 23,760 | 23,760 | 23,760 | 23,760 | 23,760 | 23,760 | 23,760 |
| | Before Covid | Mean | 48.79 | 18.37 | 30.43 | 147.17 | 43.21 | 42.69 | 61.28 | 145.11 | 96.70 | 31.29 | 17.12 | 341.07 |
| | | St. dev. | 52.01 | 25.18 | 38.75 | 164.23 | 64.91 | 57.52 | 61.28 | 175.87 | 131.20 | 56.03 | 39.42 | 329.29 |
| | | Obs. | 14,850 | 14,850 | 14,850 | 14,850 | 14,850 | 14,850 | 14,850 | 14,850 | 14,850 | 14,850 | 14,850 | 14,850 |
| | After Covid | Mean | 48.04 | 16.52 | 31.52 | 139.38 | 40.45 | 40.95 | 57.98 | 144.19 | 92.75 | 33.55 | 17.89 | 331.62 |
| | | St. dev. | 53.90 | 23.38 | 41.40 | 165.51 | 63.28 | 58.06 | 57.98 | 184.85 | 131.09 | 65.03 | 44.50 | 342.66 |
| | | Obs. | 8,910 | 8,910 | 8,910 | 8,910 | 8,910 | 8,910 | 8,910 | 8,910 | 8,910 | 8,910 | 8,910 | 8,910 |

Continues

| Retailer type | Period | Statistic | Fruit and vegetable products (pence/per capita per week) | | | | | | | | | | | Total |
|---|---|---|---|---|---|---|---|---|---|---|---|---|---|---|
| | | | Potatoes | | | Vegetables (excl. potatoes) | | | | Fruit | | | | |
| | | | **Total** | Fresh | Processed | **Total** | Fresh green | Other fresh | Processed vegetables | **Total** | Fresh fruit | Processed fruit | Fruit juices | |
| **Online** | All | Mean | 12.14 | 4.08 | 8.06 | 36.09 | 9.99 | 10.26 | 15.84 | 33.66 | 21.25 | 8.40 | 4.01 | 81.89 |
| | | St. dev. | 33.97 | 13.87 | 24.96 | 103.39 | 33.15 | 33.72 | 15.84 | 106.29 | 73.33 | 32.83 | 20.78 | 222.83 |
| | | Obs. | 23,760 | 23,760 | 23,760 | 23,760 | 23,760 | 23,760 | 23,760 | 23,760 | 23,760 | 23,760 | 23,760 | 23,760 |
| | Before Covid | Mean | 9.13 | 3.01 | 6.12 | 27.33 | 7.44 | 7.39 | 12.50 | 24.56 | 15.31 | 6.26 | 2.98 | 61.01 |
| | | St. dev. | 28.14 | 10.73 | 21.37 | 85.33 | 26.99 | 25.79 | 12.50 | 83.80 | 56.53 | 28.17 | 16.30 | 179.78 |
| | | Obs. | 14,850 | 14,850 | 14,850 | 14,850 | 14,850 | 14,850 | 14,850 | 14,850 | 14,850 | 14,850 | 14,850 | 14,850 |
| | After Covid | Mean | 17.18 | 5.87 | 11.30 | 50.69 | 14.23 | 15.04 | 21.42 | 48.83 | 31.15 | 11.96 | 5.72 | 116.69 |
| | | St. dev. | 41.45 | 17.78 | 29.73 | 126.62 | 41.08 | 43.44 | 21.42 | 134.37 | 94.11 | 39.15 | 26.52 | 276.78 |
| | | Obs. | 8,910 | 8,910 | 8,910 | 8,910 | 8,910 | 8,910 | 8,910 | 8,910 | 8,910 | 8,910 | 8,910 | 8,910 |
| **Other retailer** | All | Mean | .22 | .07 | .15 | .53 | .21 | .20 | .12 | .51 | .31 | .11 | .08 | 1.26 |
| | | St. dev. | 2.96 | .84 | 2.77 | 5.40 | 2.72 | 2.31 | .12 | 6.23 | 4.10 | 2.94 | 3.41 | 11.10 |
| | | Obs. | 23,760 | 23,760 | 23,760 | 23,760 | 23,760 | 23,760 | 23,760 | 23,760 | 23,760 | 23,760 | 23,760 | 23,760 |
| | Before Covid | Mean | .20 | .05 | .15 | .39 | .14 | .13 | .12 | .38 | .18 | .11 | .09 | .97 |
| | | St. dev. | 3.17 | .71 | 3.06 | 4.17 | 1.85 | 1.67 | .12 | 5.15 | 2.17 | 3.09 | 3.32 | 8.72 |
| | | Obs. | 14,850 | 14,850 | 14,850 | 14,850 | 14,850 | 14,850 | 14,850 | 14,850 | 14,850 | 14,850 | 14,850 | 14,850 |
| | After Covid | Mean | .24 | .09 | .15 | .77 | .32 | .32 | .13 | .72 | .52 | .12 | .08 | 1.73 |
| | | St. dev. | 2.56 | 1.01 | 2.19 | 6.99 | 3.75 | 3.09 | .13 | 7.70 | 6.08 | 2.68 | 3.56 | 14.19 |
| | | Obs. | 8,910 | 8,910 | 8,910 | 8,910 | 8,910 | 8,910 | 8,910 | 8,910 | 8,910 | 8,910 | 8,910 | 8,910 |
| **All retailers** | All | Mean | 78.88 | 29.02 | 49.86 | 241.80 | 72.14 | 73.30 | 96.36 | 246.88 | 161.96 | 57.90 | 27.02 | 567.57 |
| | | St. dev. | 58.99 | 28.61 | 47.70 | 191.18 | 77.59 | 70.43 | 96.36 | 220.95 | 165.97 | 78.07 | 50.03 | 373.81 |
| | | Obs. | 23,760 | 23,760 | 23,760 | 23,760 | 23,760 | 23,760 | 23,760 | 23,760 | 23,760 | 23,760 | 23,760 | 23,760 |
| | Before Covid | Mean | 75.60 | 28.54 | 47.07 | 234.47 | 70.23 | 70.72 | 93.53 | 235.31 | 155.65 | 54.31 | 25.34 | 545.38 |
| | | St. dev. | 56.63 | 28.32 | 45.32 | 185.11 | 76.16 | 67.70 | 93.53 | 209.27 | 159.10 | 73.12 | 46.21 | 356.59 |
| | | Obs. | 14,850 | 14,850 | 14,850 | 14,850 | 14,850 | 14,850 | 14,850 | 14,850 | 14,850 | 14,850 | 14,850 | 14,850 |
| | After Covid | Mean | 84.35 | 29.83 | 54.52 | 254.02 | 75.33 | 77.61 | 101.08 | 266.18 | 172.47 | 63.89 | 29.82 | 604.55 |
| | | St. dev. | 62.34 | 29.07 | 51.10 | 200.30 | 79.82 | 74.58 | 101.08 | 237.91 | 176.33 | 85.37 | 55.70 | 398.16 |
| | | Obs. | 8,910 | 8,910 | 8,910 | 8,910 | 8,910 | 8,910 | 8,910 | 8,910 | 8,910 | 8,910 | 8,910 | 8,910 |

Source: Own elaboration based on Kantar Worldpanel data.

# Table A11. Scotland - Average per capita purchases of fruit and vegetables before and after Covid-19 lockdown

| Retailer type | Period | Statistic | Fruit and vegetable products (grams/per capita per week) | | | | | | | | | | | Total |
|---|---|---|---|---|---|---|---|---|---|---|---|---|---|---|
| | | | Potatoes | | | Vegetables (excl. potatoes) | | | | Fruit | | | | |
| | | | **Total** | Fresh | Processed | **Total** | Fresh green | Other fresh | Processed vegetables | **Total** | Fresh fruit | Processed fruit | Fruit juices | |
| **Club and bargain store** | All | Mean | 24.49 | 7.62 | 16.87 | 23.46 | 1.05 | 1.63 | 20.78 | 18.51 | 8.16 | 6.26 | 4.09 | 66.46 |
| | | St. dev. | 86.96 | 58.35 | 60.75 | 67.32 | 7.10 | 11.09 | 20.78 | 97.56 | 82.21 | 28.93 | 34.11 | 174.75 |
| | | Obs. | 8,832 | 8,832 | 8,832 | 8,832 | 8,832 | 8,832 | 8,832 | 8,832 | 8,832 | 8,832 | 8,832 | 8,832 |
| | Before Covid | Mean | 23.31 | 7.77 | 15.54 | 23.42 | .99 | 1.73 | 20.70 | 16.16 | 7.51 | 5.64 | 3.01 | 62.89 |
| | | St. dev. | 85.01 | 61.34 | 56.84 | 67.79 | 6.84 | 11.62 | 20.70 | 77.26 | 65.91 | 26.10 | 24.34 | 160.91 |
| | | Obs. | 5,520 | 5,520 | 5,520 | 5,520 | 5,520 | 5,520 | 5,520 | 5,520 | 5,520 | 5,520 | 5,520 | 5,520 |
| | After Covid | Mean | 26.47 | 7.38 | 19.08 | 23.51 | 1.14 | 1.47 | 20.91 | 22.43 | 9.25 | 7.31 | 5.87 | 72.41 |
| | | St. dev. | 90.09 | 52.98 | 66.71 | 66.55 | 7.51 | 10.15 | 20.91 | 124.14 | 103.84 | 33.08 | 45.95 | 195.53 |
| | | Obs. | 3,312 | 3,312 | 3,312 | 3,312 | 3,312 | 3,312 | 3,312 | 3,312 | 3,312 | 3,312 | 3,312 | 3,312 |
| **Convenience** | All | Mean | 31.52 | 23.16 | 8.36 | 48.47 | 14.76 | 19.99 | 13.72 | 58.17 | 44.52 | 4.03 | 9.62 | 138.15 |
| | | St. dev. | 113.18 | 101.46 | 31.81 | 147.13 | 60.32 | 68.89 | 13.72 | 188.16 | 155.93 | 20.93 | 59.08 | 381.18 |
| | | Obs. | 8,832 | 8,832 | 8,832 | 8,832 | 8,832 | 8,832 | 8,832 | 8,832 | 8,832 | 8,832 | 8,832 | 8,832 |
| | Before Covid | Mean | 28.81 | 21.30 | 7.51 | 45.19 | 13.21 | 18.39 | 13.59 | 53.65 | 40.68 | 3.63 | 9.34 | 127.64 |
| | | St. dev. | 106.62 | 96.48 | 28.72 | 138.56 | 54.75 | 64.26 | 13.59 | 178.40 | 148.49 | 18.59 | 56.32 | 354.88 |
| | | Obs. | 5,520 | 5,520 | 5,520 | 5,520 | 5,520 | 5,520 | 5,520 | 5,520 | 5,520 | 5,520 | 5,520 | 5,520 |
| | After Covid | Mean | 36.04 | 26.25 | 9.79 | 53.92 | 17.33 | 22.64 | 13.94 | 65.70 | 50.92 | 4.69 | 10.10 | 155.66 |
| | | St. dev. | 123.23 | 109.20 | 36.33 | 160.28 | 68.54 | 75.93 | 13.94 | 203.18 | 167.41 | 24.32 | 63.42 | 420.86 |
| | | Obs. | 3,312 | 3,312 | 3,312 | 3,312 | 3,312 | 3,312 | 3,312 | 3,312 | 3,312 | 3,312 | 3,312 | 3,312 |
| **Discounter** | All | Mean | 140.34 | 95.41 | 44.94 | 289.56 | 92.90 | 99.08 | 97.58 | 296.64 | 202.51 | 27.30 | 66.83 | 726.54 |
| | | St. dev. | 252.36 | 193.58 | 106.25 | 479.77 | 178.44 | 182.98 | 97.58 | 522.98 | 383.02 | 64.26 | 223.23 | 1,099.88 |
| | | Obs. | 8,832 | 8,832 | 8,832 | 8,832 | 8,832 | 8,832 | 8,832 | 8,832 | 8,832 | 8,832 | 8,832 | 8,832 |
| | Before Covid | Mean | 136.66 | 94.04 | 42.62 | 286.33 | 90.47 | 98.77 | 97.09 | 296.67 | 206.71 | 25.21 | 64.74 | 719.66 |
| | | St. dev. | 246.09 | 190.45 | 102.35 | 468.72 | 172.22 | 182.23 | 97.09 | 521.59 | 392.86 | 61.36 | 214.00 | 1,083.07 |
| | | Obs. | 5,520 | 5,520 | 5,520 | 5,520 | 5,520 | 5,520 | 5,520 | 5,520 | 5,520 | 5,520 | 5,520 | 5,520 |
| | After Covid | Mean | 146.48 | 97.69 | 48.79 | 294.94 | 96.95 | 99.59 | 98.40 | 296.60 | 195.50 | 30.79 | 70.31 | 738.02 |
| | | St. dev. | 262.40 | 198.69 | 112.35 | 497.67 | 188.31 | 184.25 | 98.40 | 525.37 | 365.96 | 68.68 | 237.82 | 1,127.41 |
| | | Obs. | 3,312 | 3,312 | 3,312 | 3,312 | 3,312 | 3,312 | 3,312 | 3,312 | 3,312 | 3,312 | 3,312 | 3,312 |
| **Large store** | All | Mean | 311.42 | 189.86 | 121.56 | 542.47 | 148.06 | 171.98 | 222.43 | 582.04 | 379.30 | 57.26 | 145.48 | 1,435.93 |
| | | St. dev. | 332.89 | 257.06 | 162.09 | 582.18 | 223.16 | 215.96 | 222.43 | 723.88 | 567.73 | 105.51 | 308.68 | 1,345.47 |
| | | Obs. | 8,832 | 8,832 | 8,832 | 8,832 | 8,832 | 8,832 | 8,832 | 8,832 | 8,832 | 8,832 | 8,832 | 8,832 |
| | Before Covid | Mean | 312.56 | 192.54 | 120.02 | 540.89 | 145.66 | 170.02 | 225.21 | 584.88 | 381.88 | 56.79 | 146.20 | 1,438.33 |
| | | St. dev. | 329.68 | 259.09 | 157.65 | 572.18 | 218.96 | 213.54 | 225.21 | 718.06 | 564.62 | 106.93 | 307.30 | 1,316.09 |
| | | Obs. | 5,520 | 5,520 | 5,520 | 5,520 | 5,520 | 5,520 | 5,520 | 5,520 | 5,520 | 5,520 | 5,520 | 5,520 |
| | After Covid | Mean | 309.53 | 185.39 | 124.14 | 545.10 | 152.07 | 175.24 | 217.79 | 577.32 | 374.98 | 58.04 | 144.29 | 1,431.95 |
| | | St. dev. | 338.21 | 253.61 | 169.23 | 598.55 | 229.97 | 219.94 | 217.79 | 733.56 | 572.92 | 103.10 | 311.02 | 1,393.25 |
| | | Obs. | 3,312 | 3,312 | 3,312 | 3,312 | 3,312 | 3,312 | 3,312 | 3,312 | 3,312 | 3,312 | 3,312 | 3,312 |

Continues

| Retailer type | Period | Statistic | Fruit and vegetable products (grams/per capita per week) | | | | | | | | | | | Total |
|---|---|---|---|---|---|---|---|---|---|---|---|---|---|---|
| | | | Potatoes | | | Vegetables (excl. potatoes) | | | | Fruit | | | | |
| | | | **Total** | Fresh | Processed | **Total** | Fresh green | Other fresh | Processed vegetables | **Total** | Fresh fruit | Processed fruit | Fruit juices | |
| **Online** | All | Mean | 41.75 | 24.10 | 17.65 | 81.96 | 21.08 | 25.06 | 35.83 | 86.92 | 48.28 | 8.93 | 29.72 | 210.64 |
| | | St. dev. | 167.01 | 116.07 | 77.95 | 303.68 | 96.00 | 117.06 | 35.83 | 363.87 | 226.87 | 53.85 | 174.17 | 733.66 |
| | | Obs. | 8,832 | 8,832 | 8,832 | 8,832 | 8,832 | 8,832 | 8,832 | 8,832 | 8,832 | 8,832 | 8,832 | 8,832 |
| | Before Covid | Mean | 29.19 | 15.20 | 13.98 | 58.21 | 14.41 | 16.76 | 27.04 | 60.35 | 31.13 | 6.13 | 23.08 | 147.75 |
| | | St. dev. | 132.79 | 86.46 | 71.94 | 246.98 | 82.76 | 91.52 | 27.04 | 291.30 | 170.13 | 44.21 | 156.75 | 585.95 |
| | | Obs. | 5,520 | 5,520 | 5,520 | 5,520 | 5,520 | 5,520 | 5,520 | 5,520 | 5,520 | 5,520 | 5,520 | 5,520 |
| | After Covid | Mean | 62.70 | 38.93 | 23.77 | 121.54 | 32.19 | 38.89 | 50.47 | 131.21 | 76.85 | 13.59 | 40.77 | 315.45 |
| | | St. dev. | 210.48 | 152.06 | 86.72 | 376.54 | 113.87 | 149.27 | 50.47 | 456.68 | 296.18 | 66.64 | 199.39 | 919.64 |
| | | Obs. | 3,312 | 3,312 | 3,312 | 3,312 | 3,312 | 3,312 | 3,312 | 3,312 | 3,312 | 3,312 | 3,312 | 3,312 |
| **Other retailer** | All | Mean | 1.22 | .96 | .26 | 2.21 | .58 | 1.10 | .53 | 2.16 | 1.35 | .09 | .73 | 5.59 |
| | | St. dev. | 13.05 | 11.12 | 4.93 | 22.88 | 7.76 | 12.55 | .53 | 26.53 | 18.13 | 1.44 | 18.40 | 51.94 |
| | | Obs. | 8,832 | 8,832 | 8,832 | 8,832 | 8,832 | 8,832 | 8,832 | 8,832 | 8,832 | 8,832 | 8,832 | 8,832 |
| | Before Covid | Mean | .84 | .71 | .14 | 1.47 | .36 | .81 | .30 | 1.38 | .70 | .06 | .62 | 3.69 |
| | | St. dev. | 9.54 | 9.27 | 2.32 | 15.65 | 5.48 | 10.43 | .30 | 18.43 | 8.84 | 1.19 | 15.97 | 32.13 |
| | | Obs. | 5,520 | 5,520 | 5,520 | 5,520 | 5,520 | 5,520 | 5,520 | 5,520 | 5,520 | 5,520 | 5,520 | 5,520 |
| | After Covid | Mean | 1.84 | 1.38 | .46 | 3.45 | .95 | 1.59 | .92 | 3.47 | 2.42 | .13 | .91 | 8.76 |
| | | St. dev. | 17.37 | 13.64 | 7.47 | 31.38 | 10.52 | 15.43 | .92 | 36.16 | 27.29 | 1.77 | 21.86 | 73.88 |
| | | Obs. | 3,312 | 3,312 | 3,312 | 3,312 | 3,312 | 3,312 | 3,312 | 3,312 | 3,312 | 3,312 | 3,312 | 3,312 |
| **All retailers** | All | Mean | 550.75 | 341.11 | 209.64 | 988.13 | 278.43 | 318.84 | 390.86 | 1,044.45 | 684.11 | 103.87 | 256.47 | 2,583.32 |
| | | St. dev. | 411.43 | 336.36 | 207.08 | 736.81 | 297.55 | 294.70 | 390.86 | 911.65 | 723.46 | 143.52 | 427.64 | 1,560.35 |
| | | Obs. | 8,832 | 8,832 | 8,832 | 8,832 | 8,832 | 8,832 | 8,832 | 8,832 | 8,832 | 8,832 | 8,832 | 8,832 |
| | Before Covid | Mean | 531.37 | 331.56 | 199.80 | 955.52 | 265.11 | 306.49 | 383.92 | 1,013.08 | 668.62 | 97.46 | 246.99 | 2,499.96 |
| | | St. dev. | 401.45 | 329.49 | 200.61 | 722.32 | 290.63 | 288.49 | 383.92 | 888.59 | 714.02 | 138.97 | 414.15 | 1,523.01 |
| | | Obs. | 5,520 | 5,520 | 5,520 | 5,520 | 5,520 | 5,520 | 5,520 | 5,520 | 5,520 | 5,520 | 5,520 | 5,520 |
| | After Covid | Mean | 583.05 | 357.02 | 226.03 | 1,042.47 | 300.63 | 339.42 | 402.42 | 1,096.73 | 709.92 | 114.55 | 272.26 | 2,722.25 |
| | | St. dev. | 425.65 | 346.97 | 216.47 | 757.33 | 307.51 | 303.70 | 402.42 | 946.67 | 738.30 | 150.20 | 448.86 | 1,611.36 |
| | | Obs. | 3,312 | 3,312 | 3,312 | 3,312 | 3,312 | 3,312 | 3,312 | 3,312 | 3,312 | 3,312 | 3,312 | 3,312 |

Source: Own elaboration based on Kantar Worldpanel data.

## Table A12. Scotland - Average per capita expenditure of fruit and vegetables before and after Covid-19 lockdown

| Retailer type | Period | Statistic | Fruit and vegetable products (pence/per capita per week): Potatoes: **Total** | Potatoes: Fresh | Potatoes: Processed | Vegetables (excl. potatoes): **Total** | Vegetables: Fresh green | Vegetables: Other fresh | Vegetables: Processed vegetables | Fruit: **Total** | Fruit: Fresh fruit | Fruit: Processed fruit | Fruit: Fruit juices | **Total** |
|---|---|---|---|---|---|---|---|---|---|---|---|---|---|---|
| **Club and bargain store** | All | Mean | 4.84 | .42 | 4.42 | 3.96 | .21 | .28 | 3.47 | 4.60 | 1.68 | 2.58 | .34 | 13.40 |
| | | St. dev. | 13.30 | 3.30 | 12.66 | 11.64 | 1.48 | 2.07 | 3.47 | 24.72 | 20.95 | 11.24 | 2.81 | 34.51 |
| | | Obs. | 8,832 | 8,832 | 8,832 | 8,832 | 8,832 | 8,832 | 8,832 | 8,832 | 8,832 | 8,832 | 8,832 | 8,832 |
| | Before Covid | Mean | 4.51 | .42 | 4.09 | 3.79 | .20 | .27 | 3.32 | 3.96 | 1.37 | 2.32 | .28 | 12.27 |
| | | St. dev. | 12.64 | 3.51 | 11.99 | 10.98 | 1.37 | 1.97 | 3.32 | 19.22 | 15.27 | 10.43 | 2.34 | 29.52 |
| | | Obs. | 5,520 | 5,520 | 5,520 | 5,520 | 5,520 | 5,520 | 5,520 | 5,520 | 5,520 | 5,520 | 5,520 | 5,520 |
| | After Covid | Mean | 5.39 | .41 | 4.98 | 4.24 | .23 | .30 | 3.71 | 5.66 | 2.19 | 3.01 | .46 | 15.29 |
| | | St. dev. | 14.32 | 2.92 | 13.68 | 12.66 | 1.65 | 2.22 | 3.71 | 31.82 | 27.96 | 12.47 | 3.45 | 41.45 |
| | | Obs. | 3,312 | 3,312 | 3,312 | 3,312 | 3,312 | 3,312 | 3,312 | 3,312 | 3,312 | 3,312 | 3,312 | 3,312 |
| **Convenience** | All | Mean | 6.20 | 2.32 | 3.88 | 14.23 | 3.68 | 4.59 | 5.96 | 17.31 | 13.05 | 2.83 | 1.42 | 37.74 |
| | | St. dev. | 19.70 | 10.39 | 14.21 | 42.56 | 14.88 | 16.75 | 5.96 | 58.41 | 47.63 | 14.55 | 8.42 | 104.86 |
| | | Obs. | 8,832 | 8,832 | 8,832 | 8,832 | 8,832 | 8,832 | 8,832 | 8,832 | 8,832 | 8,832 | 8,832 | 8,832 |
| | Before Covid | Mean | 5.59 | 2.21 | 3.38 | 13.38 | 3.28 | 4.24 | 5.86 | 15.83 | 11.96 | 2.47 | 1.40 | 34.80 |
| | | St. dev. | 17.63 | 10.09 | 12.02 | 41.23 | 13.51 | 16.44 | 5.86 | 55.28 | 46.05 | 12.86 | 8.16 | 100.20 |
| | | Obs. | 5,520 | 5,520 | 5,520 | 5,520 | 5,520 | 5,520 | 5,520 | 5,520 | 5,520 | 5,520 | 5,520 | 5,520 |
| | After Covid | Mean | 7.22 | 2.50 | 4.73 | 15.64 | 4.34 | 5.16 | 6.14 | 19.77 | 14.87 | 3.44 | 1.46 | 42.63 |
| | | St. dev. | 22.70 | 10.88 | 17.22 | 44.65 | 16.90 | 17.24 | 6.14 | 63.23 | 50.11 | 16.97 | 8.83 | 112.04 |
| | | Obs. | 3,312 | 3,312 | 3,312 | 3,312 | 3,312 | 3,312 | 3,312 | 3,312 | 3,312 | 3,312 | 3,312 | 3,312 |
| **Discounter** | All | Mean | 14.65 | 6.08 | 8.57 | 46.35 | 15.02 | 16.12 | 15.20 | 54.86 | 38.37 | 11.26 | 5.24 | 115.86 |
| | | St. dev. | 26.44 | 12.57 | 18.90 | 76.73 | 29.47 | 29.67 | 15.20 | 95.72 | 74.45 | 26.23 | 16.58 | 177.50 |
| | | Obs. | 8,832 | 8,832 | 8,832 | 8,832 | 8,832 | 8,832 | 8,832 | 8,832 | 8,832 | 8,832 | 8,832 | 8,832 |
| | Before Covid | Mean | 14.04 | 6.01 | 8.03 | 46.13 | 14.82 | 16.24 | 15.07 | 54.46 | 38.85 | 10.65 | 4.96 | 114.63 |
| | | St. dev. | 24.87 | 12.42 | 17.33 | 75.41 | 28.67 | 29.97 | 15.07 | 95.89 | 76.22 | 25.87 | 15.62 | 175.52 |
| | | Obs. | 5,520 | 5,520 | 5,520 | 5,520 | 5,520 | 5,520 | 5,520 | 5,520 | 5,520 | 5,520 | 5,520 | 5,520 |
| | After Covid | Mean | 15.67 | 6.19 | 9.48 | 46.70 | 15.35 | 15.92 | 15.43 | 55.53 | 37.56 | 12.28 | 5.70 | 117.90 |
| | | St. dev. | 28.83 | 12.82 | 21.24 | 78.90 | 30.74 | 29.19 | 15.43 | 95.45 | 71.40 | 26.78 | 18.05 | 180.77 |
| | | Obs. | 3,312 | 3,312 | 3,312 | 3,312 | 3,312 | 3,312 | 3,312 | 3,312 | 3,312 | 3,312 | 3,312 | 3,312 |
| **Large store** | All | Mean | 49.76 | 14.68 | 35.08 | 113.60 | 29.59 | 32.81 | 51.21 | 125.72 | 82.07 | 27.22 | 16.43 | 289.08 |
| | | St. dev. | 51.19 | 20.25 | 41.83 | 131.91 | 47.91 | 46.12 | 51.21 | 163.65 | 126.89 | 51.57 | 37.36 | 291.77 |
| | | Obs. | 8,832 | 8,832 | 8,832 | 8,832 | 8,832 | 8,832 | 8,832 | 8,832 | 8,832 | 8,832 | 8,832 | 8,832 |
| | Before Covid | Mean | 49.54 | 15.26 | 34.28 | 113.87 | 29.59 | 32.75 | 51.53 | 125.19 | 81.94 | 26.79 | 16.46 | 288.60 |
| | | St. dev. | 49.43 | 20.59 | 39.73 | 129.53 | 47.56 | 46.02 | 51.53 | 160.60 | 123.95 | 51.32 | 37.00 | 284.72 |
| | | Obs. | 5,520 | 5,520 | 5,520 | 5,520 | 5,520 | 5,520 | 5,520 | 5,520 | 5,520 | 5,520 | 5,520 | 5,520 |
| | After Covid | Mean | 50.12 | 13.70 | 36.42 | 113.16 | 29.59 | 32.90 | 50.67 | 126.60 | 82.28 | 27.95 | 16.37 | 289.87 |
| | | St. dev. | 54.00 | 19.63 | 45.10 | 135.80 | 48.50 | 46.30 | 50.67 | 168.63 | 131.67 | 51.98 | 37.94 | 303.20 |
| | | Obs. | 3,312 | 3,312 | 3,312 | 3,312 | 3,312 | 3,312 | 3,312 | 3,312 | 3,312 | 3,312 | 3,312 | 3,312 |

Continues

| Retailer type | Period | Statistic | Fruit and vegetable products (pence/per capita per week) | | | | | | | | | | | Total |
|---|---|---|---|---|---|---|---|---|---|---|---|---|---|---|
| | | | Potatoes | | | Vegetables (excl. potatoes) | | | | Fruit | | | | |
| | | | **Total** | Fresh | Processed | **Total** | Fresh green | Other fresh | Processed vegetables | **Total** | Fresh fruit | Processed fruit | Fruit juices | |
| **Online** | All | Mean | 6.70 | 1.86 | 4.83 | 17.26 | 4.21 | 4.62 | 8.43 | 18.80 | 11.10 | 4.31 | 3.39 | 42.76 |
| | | St. dev. | 25.53 | 8.95 | 19.75 | 68.96 | 20.80 | 20.88 | 8.43 | 85.80 | 57.62 | 25.19 | 20.52 | 158.71 |
| | | Obs. | 8,832 | 8,832 | 8,832 | 8,832 | 8,832 | 8,832 | 8,832 | 8,832 | 8,832 | 8,832 | 8,832 | 8,832 |
| | Before Covid | Mean | 4.83 | 1.27 | 3.56 | 12.04 | 2.98 | 3.05 | 6.00 | 12.74 | 7.29 | 2.90 | 2.54 | 29.60 |
| | | St. dev. | 21.45 | 7.50 | 16.96 | 54.47 | 18.68 | 15.45 | 6.00 | 65.18 | 44.67 | 18.73 | 17.75 | 124.30 |
| | | Obs. | 5,520 | 5,520 | 5,520 | 5,520 | 5,520 | 5,520 | 5,520 | 5,520 | 5,520 | 5,520 | 5,520 | 5,520 |
| | After Covid | Mean | 9.81 | 2.85 | 6.96 | 25.97 | 6.26 | 7.23 | 12.48 | 28.89 | 17.43 | 6.65 | 4.81 | 64.68 |
| | | St. dev. | 30.93 | 10.87 | 23.53 | 87.28 | 23.79 | 27.47 | 12.48 | 111.32 | 73.92 | 33.14 | 24.39 | 201.64 |
| | | Obs. | 3,312 | 3,312 | 3,312 | 3,312 | 3,312 | 3,312 | 3,312 | 3,312 | 3,312 | 3,312 | 3,312 | 3,312 |
| **Other retailer** | All | Mean | .21 | .08 | .13 | .40 | .11 | .15 | .14 | .40 | .25 | .06 | .10 | 1.01 |
| | | St. dev. | 2.32 | .94 | 1.96 | 4.31 | 1.51 | 1.80 | .14 | 4.46 | 3.40 | 1.07 | 2.33 | 9.14 |
| | | Obs. | 8,832 | 8,832 | 8,832 | 8,832 | 8,832 | 8,832 | 8,832 | 8,832 | 8,832 | 8,832 | 8,832 | 8,832 |
| | Before Covid | Mean | .15 | .07 | .08 | .26 | .06 | .10 | .10 | .27 | .14 | .05 | .09 | .68 |
| | | St. dev. | 1.44 | .86 | 1.17 | 3.08 | .95 | 1.30 | .10 | 3.27 | 1.98 | 1.03 | 2.35 | 5.50 |
| | | Obs. | 5,520 | 5,520 | 5,520 | 5,520 | 5,520 | 5,520 | 5,520 | 5,520 | 5,520 | 5,520 | 5,520 | 5,520 |
| | After Covid | Mean | .32 | .11 | .22 | .63 | .18 | .24 | .21 | .62 | .43 | .08 | .11 | 1.56 |
| | | St. dev. | 3.30 | 1.06 | 2.82 | 5.81 | 2.13 | 2.42 | .21 | 5.93 | 4.93 | 1.13 | 2.29 | 13.11 |
| | | Obs. | 3,312 | 3,312 | 3,312 | 3,312 | 3,312 | 3,312 | 3,312 | 3,312 | 3,312 | 3,312 | 3,312 | 3,312 |
| **All retailers** | All | Mean | 82.36 | 25.44 | 56.92 | 195.80 | 52.82 | 58.57 | 84.41 | 221.68 | 146.50 | 48.27 | 26.92 | 499.84 |
| | | St. dev. | 60.28 | 25.91 | 51.20 | 162.86 | 62.09 | 59.32 | 84.41 | 212.63 | 168.28 | 68.99 | 47.88 | 345.16 |
| | | Obs. | 8,832 | 8,832 | 8,832 | 8,832 | 8,832 | 8,832 | 8,832 | 8,832 | 8,832 | 8,832 | 8,832 | 8,832 |
| | Before Covid | Mean | 78.65 | 25.24 | 53.41 | 189.47 | 50.93 | 56.67 | 81.87 | 212.46 | 141.55 | 45.18 | 25.73 | 480.58 |
| | | St. dev. | 56.73 | 25.73 | 47.29 | 158.10 | 61.33 | 58.07 | 81.87 | 203.84 | 162.96 | 65.90 | 45.94 | 334.02 |
| | | Obs. | 5,520 | 5,520 | 5,520 | 5,520 | 5,520 | 5,520 | 5,520 | 5,520 | 5,520 | 5,520 | 5,520 | 5,520 |
| | After Covid | Mean | 88.54 | 25.76 | 62.77 | 206.34 | 55.95 | 61.76 | 88.63 | 237.07 | 154.75 | 53.41 | 28.90 | 531.94 |
| | | St. dev. | 65.32 | 26.22 | 56.64 | 170.01 | 63.22 | 61.22 | 88.63 | 225.72 | 176.49 | 73.57 | 50.89 | 360.73 |
| | | Obs. | 3,312 | 3,312 | 3,312 | 3,312 | 3,312 | 3,312 | 3,312 | 3,312 | 3,312 | 3,312 | 3,312 | 3,312 |

Source: Own elaboration based on Kantar Worldpanel data.

## Table A13. Wales - Average per capita purchases of fruit and vegetables before and after Covid-19 lockdown

| Retailer type | Period | Statistic | Fruit and vegetable products (grams/per capita per week) | | | | | | | | | | | Total |
|---|---|---|---|---|---|---|---|---|---|---|---|---|---|---|
| | | | Potatoes | | | Vegetables (excl. potatoes) | | | | Fruit | | | | |
| | | | **Total** | Fresh | Processed | **Total** | Fresh green | Other fresh | Processed vegetables | **Total** | Fresh fruit | Processed fruit | Fruit juices | |
| **Club and bargain store** | All | Mean | 24.53 | 11.93 | 12.60 | 24.40 | .88 | 2.63 | 20.88 | 14.12 | 5.22 | 4.88 | 4.03 | 63.05 |
| | | St. dev. | 112.49 | 94.23 | 46.60 | 76.81 | 6.17 | 22.75 | 20.88 | 59.64 | 30.66 | 22.94 | 38.96 | 185.62 |
| | | Obs. | 5,056 | 5,056 | 5,056 | 5,056 | 5,056 | 5,056 | 5,056 | 5,056 | 5,056 | 5,056 | 5,056 | 5,056 |
| | Before Covid | Mean | 22.94 | 11.07 | 11.87 | 22.36 | .83 | 2.51 | 19.02 | 13.82 | 5.82 | 4.63 | 3.37 | 59.13 |
| | | St. dev. | 89.65 | 70.68 | 43.71 | 69.24 | 6.00 | 22.64 | 19.02 | 60.21 | 34.68 | 21.84 | 36.60 | 161.75 |
| | | Obs. | 3,160 | 3,160 | 3,160 | 3,160 | 3,160 | 3,160 | 3,160 | 3,160 | 3,160 | 3,160 | 3,160 | 3,160 |
| | After Covid | Mean | 27.18 | 13.35 | 13.83 | 27.79 | .97 | 2.84 | 23.97 | 14.62 | 4.22 | 5.28 | 5.12 | 69.59 |
| | | St. dev. | 142.64 | 123.92 | 51.04 | 87.91 | 6.45 | 22.93 | 23.97 | 58.69 | 22.38 | 24.68 | 42.59 | 219.60 |
| | | Obs. | 1,896 | 1,896 | 1,896 | 1,896 | 1,896 | 1,896 | 1,896 | 1,896 | 1,896 | 1,896 | 1,896 | 1,896 |
| **Convenience** | All | Mean | 27.08 | 21.99 | 5.09 | 43.79 | 15.06 | 18.06 | 10.66 | 40.99 | 31.56 | 2.81 | 6.62 | 111.86 |
| | | St. dev. | 98.70 | 92.43 | 20.86 | 135.96 | 60.60 | 66.89 | 10.66 | 147.92 | 122.12 | 15.08 | 52.50 | 319.05 |
| | | Obs. | 5,056 | 5,056 | 5,056 | 5,056 | 5,056 | 5,056 | 5,056 | 5,056 | 5,056 | 5,056 | 5,056 | 5,056 |
| | Before Covid | Mean | 24.69 | 20.06 | 4.63 | 39.61 | 13.33 | 16.20 | 10.09 | 37.09 | 28.52 | 2.57 | 6.00 | 101.39 |
| | | St. dev. | 93.28 | 88.14 | 18.52 | 124.77 | 54.39 | 62.78 | 10.09 | 146.10 | 116.62 | 14.94 | 56.49 | 302.34 |
| | | Obs. | 3,160 | 3,160 | 3,160 | 3,160 | 3,160 | 3,160 | 3,160 | 3,160 | 3,160 | 3,160 | 3,160 | 3,160 |
| | After Covid | Mean | 31.08 | 25.21 | 5.87 | 50.74 | 17.96 | 21.15 | 11.62 | 47.48 | 36.62 | 3.21 | 7.66 | 129.30 |
| | | St. dev. | 107.04 | 99.09 | 24.24 | 152.58 | 69.66 | 73.15 | 11.62 | 150.71 | 130.65 | 15.32 | 45.06 | 344.49 |
| | | Obs. | 1,896 | 1,896 | 1,896 | 1,896 | 1,896 | 1,896 | 1,896 | 1,896 | 1,896 | 1,896 | 1,896 | 1,896 |
| **Discounter** | All | Mean | 167.61 | 116.18 | 51.42 | 327.30 | 102.48 | 113.88 | 110.94 | 279.12 | 185.43 | 32.66 | 61.03 | 774.02 |
| | | St. dev. | 298.93 | 238.19 | 114.66 | 608.41 | 216.20 | 256.07 | 110.94 | 508.38 | 388.72 | 92.61 | 179.23 | 1,256.10 |
| | | Obs. | 5,056 | 5,056 | 5,056 | 5,056 | 5,056 | 5,056 | 5,056 | 5,056 | 5,056 | 5,056 | 5,056 | 5,056 |
| | Before Covid | Mean | 163.95 | 111.76 | 52.18 | 329.21 | 103.50 | 111.98 | 113.73 | 282.51 | 190.28 | 32.64 | 59.59 | 775.67 |
| | | St. dev. | 289.68 | 226.12 | 116.28 | 595.23 | 213.57 | 239.51 | 113.73 | 510.39 | 396.69 | 94.36 | 177.74 | 1,239.50 |
| | | Obs. | 3,160 | 3,160 | 3,160 | 3,160 | 3,160 | 3,160 | 3,160 | 3,160 | 3,160 | 3,160 | 3,160 | 3,160 |
| | After Covid | Mean | 173.70 | 123.55 | 50.16 | 324.10 | 100.78 | 117.04 | 106.28 | 273.47 | 177.33 | 32.71 | 63.43 | 771.28 |
| | | St. dev. | 313.72 | 256.94 | 111.93 | 629.92 | 220.55 | 281.54 | 106.28 | 505.11 | 375.04 | 89.64 | 181.72 | 1,283.61 |
| | | Obs. | 1,896 | 1,896 | 1,896 | 1,896 | 1,896 | 1,896 | 1,896 | 1,896 | 1,896 | 1,896 | 1,896 | 1,896 |
| **Large store** | All | Mean | 404.62 | 267.80 | 136.82 | 608.78 | 161.41 | 188.48 | 258.89 | 540.78 | 346.78 | 52.36 | 141.64 | 1,554.19 |
| | | St. dev. | 438.41 | 366.89 | 184.88 | 615.34 | 218.74 | 237.41 | 258.89 | 669.07 | 481.48 | 108.66 | 318.34 | 1,422.78 |
| | | Obs. | 5,056 | 5,056 | 5,056 | 5,056 | 5,056 | 5,056 | 5,056 | 5,056 | 5,056 | 5,056 | 5,056 | 5,056 |
| | Before Covid | Mean | 397.86 | 263.22 | 134.64 | 608.84 | 157.42 | 188.13 | 263.29 | 536.67 | 348.33 | 51.68 | 136.67 | 1,543.38 |
| | | St. dev. | 431.25 | 361.83 | 177.91 | 620.90 | 214.06 | 242.31 | 263.29 | 666.90 | 485.01 | 104.23 | 310.89 | 1,425.96 |
| | | Obs. | 3,160 | 3,160 | 3,160 | 3,160 | 3,160 | 3,160 | 3,160 | 3,160 | 3,160 | 3,160 | 3,160 | 3,160 |
| | After Covid | Mean | 415.89 | 275.43 | 140.46 | 608.68 | 168.07 | 189.05 | 251.56 | 547.63 | 344.21 | 53.50 | 149.92 | 1,572.20 |
| | | St. dev. | 449.98 | 375.14 | 195.95 | 606.11 | 226.23 | 229.08 | 251.56 | 672.79 | 475.67 | 115.68 | 330.31 | 1,417.65 |
| | | Obs. | 1,896 | 1,896 | 1,896 | 1,896 | 1,896 | 1,896 | 1,896 | 1,896 | 1,896 | 1,896 | 1,896 | 1,896 |

Continues

| Retailer type | Period | Statistic | Fruit and vegetable products (grams/per capita per week) | | | | | | | | | | | Total |
|---|---|---|---|---|---|---|---|---|---|---|---|---|---|---|
| | | | Potatoes | | | Vegetables (excl. potatoes) | | | | Fruit | | | | |
| | | | **Total** | Fresh | Processed | **Total** | Fresh green | Other fresh | Processed vegetables | **Total** | Fresh fruit | Processed fruit | Fruit juices | |
| **Online** | All | Mean | 66.04 | 41.89 | 24.15 | 126.46 | 32.24 | 36.28 | 57.93 | 103.94 | 66.02 | 10.46 | 27.46 | 296.44 |
| | | St. dev. | 223.88 | 171.07 | 84.79 | 389.07 | 117.32 | 130.25 | 57.93 | 374.63 | 254.91 | 50.26 | 143.99 | 881.05 |
| | | Obs. | 5,056 | 5,056 | 5,056 | 5,056 | 5,056 | 5,056 | 5,056 | 5,056 | 5,056 | 5,056 | 5,056 | 5,056 |
| | Before Covid | Mean | 49.87 | 30.61 | 19.25 | 95.22 | 23.85 | 26.23 | 45.15 | 69.35 | 43.72 | 6.47 | 19.15 | 214.44 |
| | | St. dev. | 195.03 | 146.24 | 76.50 | 336.40 | 101.15 | 107.59 | 45.15 | 288.22 | 183.86 | 34.43 | 112.99 | 730.04 |
| | | Obs. | 3,160 | 3,160 | 3,160 | 3,160 | 3,160 | 3,160 | 3,160 | 3,160 | 3,160 | 3,160 | 3,160 | 3,160 |
| | After Covid | Mean | 93.00 | 60.68 | 32.32 | 178.51 | 46.22 | 53.05 | 79.24 | 161.59 | 103.19 | 17.10 | 41.30 | 433.10 |
| | | St. dev. | 262.94 | 204.58 | 96.52 | 459.15 | 139.09 | 159.73 | 79.24 | 480.20 | 338.77 | 68.48 | 183.63 | 1,073.47 |
| | | Obs. | 1,896 | 1,896 | 1,896 | 1,896 | 1,896 | 1,896 | 1,896 | 1,896 | 1,896 | 1,896 | 1,896 | 1,896 |
| **Other retailer** | All | Mean | 1.53 | 1.26 | .27 | 2.22 | .94 | .96 | .33 | 3.35 | 1.97 | .20 | 1.17 | 7.10 |
| | | St. dev. | 18.21 | 16.17 | 3.93 | 31.98 | 17.11 | 15.33 | .33 | 39.18 | 28.39 | 5.15 | 22.86 | 74.98 |
| | | Obs. | 5,056 | 5,056 | 5,056 | 5,056 | 5,056 | 5,056 | 5,056 | 5,056 | 5,056 | 5,056 | 5,056 | 5,056 |
| | Before Covid | Mean | 1.13 | .82 | .30 | 1.01 | .37 | .33 | .30 | 1.89 | 1.14 | .20 | .55 | 4.03 |
| | | St. dev. | 16.29 | 13.82 | 4.39 | 11.58 | 6.12 | 5.11 | .30 | 21.56 | 16.81 | 5.37 | 11.97 | 37.26 |
| | | Obs. | 3,160 | 3,160 | 3,160 | 3,160 | 3,160 | 3,160 | 3,160 | 3,160 | 3,160 | 3,160 | 3,160 | 3,160 |
| | After Covid | Mean | 2.21 | 2.00 | .21 | 4.25 | 1.89 | 1.99 | .37 | 5.77 | 3.36 | .21 | 2.21 | 12.23 |
| | | St. dev. | 21.01 | 19.45 | 2.99 | 49.99 | 26.78 | 24.12 | .37 | 57.53 | 40.94 | 4.77 | 33.96 | 112.43 |
| | | Obs. | 1,896 | 1,896 | 1,896 | 1,896 | 1,896 | 1,896 | 1,896 | 1,896 | 1,896 | 1,896 | 1,896 | 1,896 |
| **All retailers** | All | Mean | 691.42 | 461.05 | 230.37 | 1,132.94 | 313.02 | 360.29 | 459.63 | 982.30 | 636.98 | 103.37 | 241.95 | 2,806.66 |
| | | St. dev. | 528.99 | 463.75 | 223.31 | 859.75 | 323.40 | 357.36 | 459.63 | 879.33 | 658.27 | 157.59 | 402.08 | 1,762.24 |
| | | Obs. | 5,056 | 5,056 | 5,056 | 5,056 | 5,056 | 5,056 | 5,056 | 5,056 | 5,056 | 5,056 | 5,056 | 5,056 |
| | Before Covid | Mean | 660.43 | 437.55 | 222.88 | 1,096.26 | 299.29 | 345.38 | 451.59 | 941.34 | 617.81 | 98.19 | 225.34 | 2,698.03 |
| | | St. dev. | 512.47 | 445.05 | 216.07 | 844.36 | 312.57 | 341.10 | 451.59 | 862.74 | 649.04 | 152.79 | 379.83 | 1,739.13 |
| | | Obs. | 3,160 | 3,160 | 3,160 | 3,160 | 3,160 | 3,160 | 3,160 | 3,160 | 3,160 | 3,160 | 3,160 | 3,160 |
| | After Covid | Mean | 743.07 | 500.22 | 242.85 | 1,194.08 | 335.91 | 385.13 | 473.04 | 1,050.57 | 668.92 | 112.01 | 269.63 | 2,987.71 |
| | | St. dev. | 551.71 | 490.96 | 234.42 | 881.64 | 339.54 | 381.73 | 473.04 | 902.40 | 672.33 | 164.95 | 435.35 | 1,785.92 |
| | | Obs. | 1,896 | 1,896 | 1,896 | 1,896 | 1,896 | 1,896 | 1,896 | 1,896 | 1,896 | 1,896 | 1,896 | 1,896 |

Source: Own elaboration based on Kantar Worldpanel data.

## Table A14. Wales - Average per capita expenditure of fruit and vegetables before and after Covid-19 lockdown

| Retailer type | Period | Statistic | Fruit and vegetable products (pence/per capita per week) | | | | | | | | | | | Total |
|---|---|---|---|---|---|---|---|---|---|---|---|---|---|---|
| | | | Potatoes | | | Vegetables (excl. potatoes) | | | | Fruit | | | | |
| | | | **Total** | Fresh | Processed | **Total** | Fresh green | Other fresh | Processed vegetables | **Total** | Fresh fruit | Processed fruit | Fruit juices | |
| **Club and bargain store** | All | Mean | 4.30 | .60 | 3.70 | 3.92 | .17 | .42 | 3.33 | 3.48 | .98 | 2.20 | .31 | 11.71 |
| | | St. dev. | 13.23 | 4.04 | 11.98 | 12.59 | 1.12 | 3.74 | 3.33 | 13.64 | 8.15 | 9.32 | 2.97 | 29.25 |
| | | Obs. | 5,056 | 5,056 | 5,056 | 5,056 | 5,056 | 5,056 | 5,056 | 5,056 | 5,056 | 5,056 | 5,056 | 5,056 |
| | Before Covid | Mean | 4.02 | .58 | 3.44 | 3.52 | .16 | .41 | 2.95 | 3.43 | 1.09 | 2.06 | .28 | 10.97 |
| | | St. dev. | 11.60 | 3.64 | 10.46 | 10.75 | 1.12 | 3.72 | 2.95 | 14.43 | 9.59 | 9.03 | 2.88 | 26.43 |
| | | Obs. | 3,160 | 3,160 | 3,160 | 3,160 | 3,160 | 3,160 | 3,160 | 3,160 | 3,160 | 3,160 | 3,160 | 3,160 |
| | After Covid | Mean | 4.78 | .62 | 4.15 | 4.58 | .18 | .45 | 3.95 | 3.57 | .78 | 2.41 | .37 | 12.93 |
| | | St. dev. | 15.55 | 4.64 | 14.15 | 15.16 | 1.11 | 3.79 | 3.95 | 12.20 | 4.91 | 9.79 | 3.12 | 33.41 |
| | | Obs. | 1,896 | 1,896 | 1,896 | 1,896 | 1,896 | 1,896 | 1,896 | 1,896 | 1,896 | 1,896 | 1,896 | 1,896 |
| **Convenience** | All | Mean | 4.16 | 2.09 | 2.07 | 11.34 | 3.26 | 3.88 | 4.21 | 11.59 | 8.52 | 2.11 | .97 | 27.10 |
| | | St. dev. | 12.53 | 8.51 | 7.54 | 36.30 | 12.76 | 14.20 | 4.21 | 43.25 | 35.09 | 13.08 | 7.44 | 81.16 |
| | | Obs. | 5,056 | 5,056 | 5,056 | 5,056 | 5,056 | 5,056 | 5,056 | 5,056 | 5,056 | 5,056 | 5,056 | 5,056 |
| | Before Covid | Mean | 3.85 | 2.03 | 1.82 | 10.65 | 2.97 | 3.59 | 4.09 | 10.70 | 7.90 | 1.96 | .84 | 25.19 |
| | | St. dev. | 11.45 | 8.68 | 6.21 | 36.69 | 12.20 | 14.03 | 4.09 | 43.15 | 35.15 | 13.95 | 7.10 | 81.14 |
| | | Obs. | 3,160 | 3,160 | 3,160 | 3,160 | 3,160 | 3,160 | 3,160 | 3,160 | 3,160 | 3,160 | 3,160 | 3,160 |
| | After Covid | Mean | 4.69 | 2.18 | 2.50 | 12.50 | 3.75 | 4.36 | 4.40 | 13.09 | 9.55 | 2.35 | 1.19 | 30.28 |
| | | St. dev. | 14.14 | 8.21 | 9.34 | 35.62 | 13.63 | 14.48 | 4.40 | 43.38 | 34.98 | 11.49 | 7.97 | 81.12 |
| | | Obs. | 1,896 | 1,896 | 1,896 | 1,896 | 1,896 | 1,896 | 1,896 | 1,896 | 1,896 | 1,896 | 1,896 | 1,896 |
| **Discounter** | All | Mean | 16.19 | 7.21 | 8.99 | 49.28 | 15.90 | 17.59 | 15.79 | 52.78 | 35.21 | 13.04 | 4.52 | 118.25 |
| | | St. dev. | 26.96 | 14.59 | 17.30 | 96.01 | 33.95 | 38.32 | 15.79 | 104.22 | 75.13 | 40.94 | 13.09 | 202.15 |
| | | Obs. | 5,056 | 5,056 | 5,056 | 5,056 | 5,056 | 5,056 | 5,056 | 5,056 | 5,056 | 5,056 | 5,056 | 5,056 |
| | Before Covid | Mean | 15.97 | 7.13 | 8.84 | 50.16 | 16.19 | 17.91 | 16.05 | 53.13 | 35.60 | 13.16 | 4.36 | 119.26 |
| | | St. dev. | 26.28 | 14.15 | 17.07 | 95.11 | 33.78 | 38.15 | 16.05 | 103.81 | 75.35 | 42.06 | 12.63 | 200.64 |
| | | Obs. | 3,160 | 3,160 | 3,160 | 3,160 | 3,160 | 3,160 | 3,160 | 3,160 | 3,160 | 3,160 | 3,160 | 3,160 |
| | After Covid | Mean | 16.56 | 7.33 | 9.23 | 47.81 | 15.40 | 17.06 | 15.35 | 52.19 | 34.57 | 12.83 | 4.79 | 116.56 |
| | | St. dev. | 28.05 | 15.31 | 17.69 | 97.52 | 34.24 | 38.60 | 15.35 | 104.91 | 74.78 | 39.01 | 13.82 | 204.68 |
| | | Obs. | 1,896 | 1,896 | 1,896 | 1,896 | 1,896 | 1,896 | 1,896 | 1,896 | 1,896 | 1,896 | 1,896 | 1,896 |
| **Large store** | All | Mean | 54.99 | 19.74 | 35.25 | 121.14 | 31.25 | 34.16 | 55.73 | 114.82 | 76.19 | 23.38 | 15.25 | 290.94 |
| | | St. dev. | 57.67 | 27.70 | 45.52 | 133.52 | 45.90 | 46.34 | 55.73 | 154.35 | 113.99 | 48.67 | 36.36 | 288.07 |
| | | Obs. | 5,056 | 5,056 | 5,056 | 5,056 | 5,056 | 5,056 | 5,056 | 5,056 | 5,056 | 5,056 | 5,056 | 5,056 |
| | Before Covid | Mean | 55.04 | 20.01 | 35.03 | 121.10 | 30.73 | 34.09 | 56.28 | 113.76 | 76.22 | 22.84 | 14.70 | 289.90 |
| | | St. dev. | 57.52 | 28.08 | 44.86 | 133.09 | 45.18 | 46.48 | 56.28 | 154.32 | 113.60 | 47.47 | 34.97 | 288.82 |
| | | Obs. | 3,160 | 3,160 | 3,160 | 3,160 | 3,160 | 3,160 | 3,160 | 3,160 | 3,160 | 3,160 | 3,160 | 3,160 |
| | After Covid | Mean | 54.90 | 19.28 | 35.62 | 121.20 | 32.12 | 34.26 | 54.83 | 116.58 | 76.13 | 24.29 | 16.15 | 292.69 |
| | | St. dev. | 57.94 | 27.05 | 46.61 | 134.26 | 47.07 | 46.12 | 54.83 | 154.44 | 114.66 | 50.60 | 38.55 | 286.89 |
| | | Obs. | 1,896 | 1,896 | 1,896 | 1,896 | 1,896 | 1,896 | 1,896 | 1,896 | 1,896 | 1,896 | 1,896 | 1,896 |

Continues

| Retailer type | Period | Statistic | Fruit and vegetable products (pence/per capita per week) | | | | | | | | | | | Total |
|---|---|---|---|---|---|---|---|---|---|---|---|---|---|---|
| | | | Potatoes | | | Vegetables (excl. potatoes) | | | | Fruit | | | | |
| | | | **Total** | Fresh | Processed | **Total** | Fresh green | Other fresh | Processed vegetables | **Total** | Fresh fruit | Processed fruit | Fruit juices | |
| **Online** | All | Mean | 9.78 | 3.11 | 6.67 | 25.43 | 6.38 | 6.91 | 12.14 | 22.27 | 14.34 | 5.03 | 2.89 | 57.48 |
| | | St. dev. | 32.59 | 12.01 | 24.52 | 85.38 | 23.99 | 25.76 | 12.14 | 88.24 | 61.25 | 25.19 | 16.75 | 186.75 |
| | | Obs. | 5,056 | 5,056 | 5,056 | 5,056 | 5,056 | 5,056 | 5,056 | 5,056 | 5,056 | 5,056 | 5,056 | 5,056 |
| | Before Covid | Mean | 7.50 | 2.35 | 5.15 | 19.14 | 4.78 | 5.01 | 9.35 | 14.86 | 9.64 | 3.23 | 1.99 | 41.50 |
| | | St. dev. | 29.30 | 10.76 | 22.31 | 74.84 | 20.29 | 21.31 | 9.35 | 67.28 | 43.27 | 20.52 | 12.51 | 156.30 |
| | | Obs. | 3,160 | 3,160 | 3,160 | 3,160 | 3,160 | 3,160 | 3,160 | 3,160 | 3,160 | 3,160 | 3,160 | 3,160 |
| | After Covid | Mean | 13.58 | 4.37 | 9.21 | 35.92 | 9.05 | 10.08 | 16.79 | 34.61 | 22.18 | 8.03 | 4.40 | 84.11 |
| | | St. dev. | 37.13 | 13.75 | 27.63 | 99.67 | 28.93 | 31.56 | 16.79 | 113.93 | 82.39 | 31.24 | 22.00 | 226.22 |
| | | Obs. | 1,896 | 1,896 | 1,896 | 1,896 | 1,896 | 1,896 | 1,896 | 1,896 | 1,896 | 1,896 | 1,896 | 1,896 |
| **Other retailer** | All | Mean | .25 | .10 | .14 | .41 | .17 | .16 | .08 | .62 | .34 | .17 | .12 | 1.27 |
| | | St. dev. | 2.81 | 1.38 | 1.94 | 5.28 | 2.79 | 2.53 | .08 | 9.79 | 5.04 | 7.72 | 2.24 | 13.98 |
| | | Obs. | 5,056 | 5,056 | 5,056 | 5,056 | 5,056 | 5,056 | 5,056 | 5,056 | 5,056 | 5,056 | 5,056 | 5,056 |
| | Before Covid | Mean | .23 | .08 | .15 | .21 | .07 | .06 | .08 | .47 | .20 | .21 | .05 | .91 |
| | | St. dev. | 3.01 | 1.30 | 2.07 | 2.36 | 1.03 | 1.15 | .08 | 10.33 | 3.36 | 9.71 | .98 | 11.80 |
| | | Obs. | 3,160 | 3,160 | 3,160 | 3,160 | 3,160 | 3,160 | 3,160 | 3,160 | 3,160 | 3,160 | 3,160 | 3,160 |
| | After Covid | Mean | .28 | .15 | .13 | .73 | .33 | .31 | .09 | .86 | .56 | .08 | .22 | 1.87 |
| | | St. dev. | 2.44 | 1.51 | 1.71 | 8.06 | 4.35 | 3.85 | .09 | 8.83 | 6.99 | 1.44 | 3.42 | 16.98 |
| | | Obs. | 1,896 | 1,896 | 1,896 | 1,896 | 1,896 | 1,896 | 1,896 | 1,896 | 1,896 | 1,896 | 1,896 | 1,896 |
| **All retailers** | All | Mean | 89.67 | 32.85 | 56.83 | 211.51 | 57.12 | 63.11 | 91.28 | 205.56 | 135.58 | 45.92 | 24.06 | 506.75 |
| | | St. dev. | 65.33 | 33.22 | 53.36 | 177.70 | 62.37 | 64.29 | 91.28 | 206.62 | 152.79 | 73.23 | 43.43 | 361.05 |
| | | Obs. | 5,056 | 5,056 | 5,056 | 5,056 | 5,056 | 5,056 | 5,056 | 5,056 | 5,056 | 5,056 | 5,056 | 5,056 |
| | Before Covid | Mean | 86.61 | 32.19 | 54.42 | 204.78 | 54.90 | 61.07 | 88.80 | 196.35 | 130.66 | 43.47 | 22.22 | 487.73 |
| | | St. dev. | 64.05 | 33.39 | 51.36 | 175.48 | 60.38 | 63.06 | 88.80 | 203.63 | 149.58 | 72.45 | 39.91 | 359.15 |
| | | Obs. | 3,160 | 3,160 | 3,160 | 3,160 | 3,160 | 3,160 | 3,160 | 3,160 | 3,160 | 3,160 | 3,160 | 3,160 |
| | After Covid | Mean | 94.79 | 33.95 | 60.84 | 222.75 | 60.82 | 66.51 | 95.42 | 220.91 | 143.78 | 50.00 | 27.13 | 538.44 |
| | | St. dev. | 67.10 | 32.91 | 56.33 | 180.82 | 65.40 | 66.17 | 95.42 | 210.68 | 157.71 | 74.35 | 48.59 | 362.06 |
| | | Obs. | 1,896 | 1,896 | 1,896 | 1,896 | 1,896 | 1,896 | 1,896 | 1,896 | 1,896 | 1,896 | 1,896 | 1,896 |

Source: Own elaboration based on Kantar Worldpanel data.

## Table A15: Difference in the weekly average per capita purchases of fruit and vegetables before and during COVID-19 pandemic

| | Difference of fruit and vegetable products (grams/per capita per week) | | | | | | | | | | | Total |
|---|---|---|---|---|---|---|---|---|---|---|---|---|
| | Potatoes | | | Vegetables (excl. potatoes) | | | | Fruit | | | | |
| | **Total** | Fresh | Processed | **Total** | Fresh green | Other fresh | Processed vegetables | **Total** | Fresh fruit | Processed fruit | Fruit juices | |
| **East** | | | | | | | | | | | | |
| Club and bargain store | 0.16 | -0.36 | 0.52 | 1.02 | -0.17 | 0.01 | 1.18* | 0.31 | -0.23 | -0.43 | 0.97 | 1.49 |
| Convenience | 4.66* | 4.05* | 0.62 | 21.97* | 7.78* | 11.13* | 3.07* | 26.23* | 22.68* | 0.94 | 2.61 | 52.86* |
| Discounter | 8.38 | 7.51* | 0.87 | 4.18 | -0.62 | -0.22 | 5.01* | 13.20 | 0.87 | 3.56* | 8.76* | 25.76 |
| Large store | -1.50 | -3.92 | 2.41 | -18.30 | -2.12 | -5.21 | -10.97* | -30.13 | -27.90* | -0.34 | -1.89 | -49.93 |
| Online | 46.16* | 30.58* | 15.58* | 91.31* | 30.65* | 30.06* | 30.60* | 91.55* | 57.27* | 10.02* | 24.25* | 229.02* |
| Other retailer | 0.36* | 0.32* | 0.04 | 1.79* | 0.73* | 1.15* | -0.09* | 2.07* | 1.89* | -0.05 | 0.23 | 4.21* |
| All retailers | 58.22* | 38.18* | 20.04* | 101.97* | 36.24* | 36.92* | 28.81* | 103.22* | 54.58* | 13.70* | 34.94* | 263.41* |
| **London** | | | | | | | | | | | | |
| Club and bargain store | 1.00 | 0.56 | 0.44 | 0.68 | 0.06 | 0.39 | 0.23* | 2.79 | 1.35 | 0.10 | 1.34* | 4.47 |
| Convenience | 7.98* | 6.30* | 1.68* | 15.70* | 4.59* | 7.61* | 3.50* | 18.01* | 12.67* | 1.33 | 4.02 | 41.70* |
| Discounter | 3.47 | 1.42 | 2.05 | 8.39 | 4.47 | 1.34 | 2.59 | 12.92 | 7.69 | 4.40* | 0.83 | 24.78 |
| Large store | 14.51 | 11.92 | 2.59 | 19.61 | 17.59* | 9.35 | -7.33 | 26.55 | 18.80 | 0.05 | 7.70 | 60.67 |
| Online | 39.17* | 29.63* | 9.54* | 92.68* | 34.02* | 31.39* | 27.28* | 88.66* | 54.30* | 10.40* | 23.96* | 220.51* |
| Other retailer | 0.27 | 0.26 | 0.01 | 1.04 | 0.45 | 0.41 | 0.18* | 0.62 | 1.48 | 0.06 | -0.92 | 1.93 |
| All retailers | 66.40* | 50.08* | 16.32* | 138.10* | 61.16* | 50.48* | 26.45* | 149.54* | 96.29* | 16.33* | 36.93* | 354.05* |
| **Midlands** | | | | | | | | | | | | |
| Club and bargain store | 0.42 | -0.55 | 0.97 | 0.04 | 0.02 | 0.03 | -0.02 | -0.07 | -0.80 | -0.01 | 0.74 | 0.40 |
| Convenience | 4.79* | 3.48* | 1.30* | 10.60* | 2.83* | 5.19* | 2.58* | 4.88 | 1.58 | 0.55 | 2.74* | 20.27* |
| Discounter | 6.93 | 4.86 | 2.08 | 8.14 | 3.35 | 3.62 | 1.17 | 2.53 | -5.20 | 3.15* | 4.58 | 17.60 |
| Large store | 14.79* | 6.62 | 8.17* | 1.30 | 8.87* | 5.62 | -13.19* | 5.94 | -6.28 | 2.77 | 9.45* | 22.02 |
| Online | 35.67* | 22.96* | 12.71* | 76.38* | 22.22* | 26.18* | 27.98* | 71.16* | 48.20* | 6.83* | 16.14* | 183.21* |
| Other retailer | 1.00* | 1.04* | -0.04 | 4.03* | 1.72* | 2.51* | -0.20* | 3.78* | 3.46* | 0.00 | 0.32 | 8.81* |
| All retailers | 63.60* | 38.41* | 25.19* | 100.49* | 39.02* | 43.15* | 18.33* | 88.21* | 40.96* | 13.28* | 33.97* | 252.31* |
| **North** | | | | | | | | | | | | |
| Club and bargain store | 0.20 | -0.71 | 0.90 | -0.61 | 0.09 | -0.10 | -0.59* | 0.69 | -0.11 | 0.25 | 0.56 | 0.29 |
| Convenience | 3.17* | 1.82 | 1.35* | 10.31* | 3.02* | 5.45* | 1.84* | 9.15* | 6.57* | 0.51 | 2.07* | 22.63* |
| Discounter | 8.56* | 5.85 | 2.71 | -4.30 | -2.24 | -0.02 | -2.04 | 5.90 | -6.11 | 5.10* | 6.91* | 10.15 |

Continues

| | Difference of fruit and vegetable products (grams/per capita per week) | | | | | | | | | | | Total |
|---|---|---|---|---|---|---|---|---|---|---|---|---|
| | **Potatoes** | | | **Vegetables (excl. potatoes)** | | | | **Fruit** | | | | |
| | **Total** | Fresh | Processed | **Total** | Fresh green | Other fresh | Processed vegetables | **Total** | Fresh fruit | Processed fruit | Fruit juices | |
| Large store | 9.97* | 9.61* | 0.37 | 7.63 | 9.54* | 8.65* | -10.56* | 8.68 | -0.07 | 3.22* | 5.54 | 26.29 |
| Online | 41.54* | 30.41* | 11.12* | 75.57* | 23.77* | 24.77* | 27.04* | 69.39* | 44.83* | 8.34* | 16.22* | 186.50* |
| Other retailer | 0.84* | 0.76* | 0.08 | 2.83* | 1.26* | 1.14* | 0.43* | 2.23* | 1.80* | 0.04 | 0.39* | 5.90* |
| All retailers | 64.28* | 47.74* | 16.54* | 91.43* | 35.42* | 39.90* | 16.11* | 96.03* | 46.90* | 17.44* | 31.69* | 251.74* |
| **South** | | | | | | | | | | | | |
| Club and bargain store | -0.18 | 0.09 | -0.27 | -0.10 | 0.11 | 0.09 | -0.30* | 0.25 | 0.01 | -0.42 | 0.66* | -0.03 |
| Convenience | 6.96* | 5.54* | 1.42* | 17.79* | 6.55* | 7.54* | 3.70* | 14.74* | 12.28* | 1.04* | 1.42 | 39.49* |
| Discounter | 1.19 | 0.39 | 0.80 | -4.97 | -2.46 | -1.57 | -0.94 | 0.34 | -9.16* | 3.65* | 5.84* | -3.44 |
| Large store | 0.16 | -2.54 | 2.70 | -18.14 | -3.32 | -1.84 | -12.97* | -23.41* | -30.22* | 3.37* | 3.44 | -41.40* |
| Online | 56.26* | 39.47* | 16.79* | 106.63* | 33.80* | 36.99* | 35.84* | 98.75* | 66.30* | 12.54* | 19.91* | 261.64* |
| Other retailer | 0.58* | 0.57* | 0.01 | 2.10* | 0.90* | 1.09* | 0.11* | 1.83* | 1.82* | 0.05 | -0.03 | 4.51* |
| All retailers | 64.96* | 43.51* | 21.45* | 103.31* | 35.57* | 42.30* | 25.44* | 92.50* | 41.01* | 20.23* | 31.25* | 260.77* |
| **Scotland** | | | | | | | | | | | | |
| Club and bargain store | 3.16 | -0.38 | 3.54* | 0.08 | 0.14 | -0.27 | 0.21 | 6.27* | 1.74 | 1.67* | 2.86* | 9.51* |
| Convenience | 7.23* | 4.95* | 2.28* | 8.73* | 4.12* | 4.25* | 0.36 | 12.05* | 10.24* | 1.06* | 0.76 | 28.02* |
| Discounter | 9.82 | 3.64 | 6.17* | 8.61 | 6.48 | 0.82 | 1.31 | -0.06 | -11.21 | 5.58* | 5.57 | 18.37 |
| Large store | -3.03 | -7.15 | 4.12 | 4.21 | 6.41 | 5.22 | -7.42 | -7.56 | -6.90 | 1.25 | -1.91 | -6.38 |
| Online | 33.51* | 23.72* | 9.79* | 63.33* | 17.78* | 22.13* | 23.43* | 70.86* | 45.72* | 7.45* | 17.69* | 167.70* |
| Other retailer | 0.99* | 0.67* | 0.33* | 1.98* | 0.58* | 0.78* | 0.62* | 2.09* | 1.73* | 0.07 | 0.30 | 5.07* |
| All retailers | 51.68* | 25.45* | 26.23* | 86.95* | 35.52* | 32.93* | 18.50* | 83.65* | 41.30* | 17.08* | 25.26* | 222.29* |
| **Wales** | | | | | | | | | | | | |
| Club and bargain store | 4.24 | 2.28 | 1.96 | 5.43* | 0.14 | 0.33 | 4.95* | 0.80 | -1.60* | 0.65 | 1.75 | 10.47 |
| Convenience | 6.39* | 5.15 | 1.24 | 11.12* | 4.64* | 4.95* | 1.53* | 10.39* | 8.10* | 0.64 | 1.66 | 27.90* |
| Discounter | 9.76 | 11.78 | -2.03 | -5.11 | -2.71 | 5.06 | -7.46* | -9.04 | -12.96 | 0.07 | 3.84 | -4.39 |
| Large store | 18.03 | 12.22 | 5.82 | -0.16 | 10.66 | 0.92 | -11.74 | 10.95 | -4.12 | 1.82 | 13.24 | 28.83 |
| Online | 43.13* | 30.07* | 13.07* | 83.28* | 22.37* | 26.82* | 34.09* | 92.24* | 59.47* | 10.63* | 22.15* | 218.66* |
| Other retailer | 1.08 | 1.17* | -0.09 | 3.25* | 1.53* | 1.66* | 0.06* | 3.88* | 2.22* | 0.01 | 1.65* | 8.21* |
| All retailers | 82.63* | 62.67* | 19.97* | 97.81* | 36.62* | 39.75* | 21.44 | 109.23* | 51.11* | 13.82* | 44.29* | 289.67* |

Source: Own elaboration based on Kantar Worldpanel data.
Note: '*' stands for statistically significant at 5 per cent.

## Table A16: Retailer importance on the expenditure of fruit and vegetables before and during COVID-19 lockdown

| | Period | Fruit and vegetable products (percentages) | | | | | | | | | | | Total |
|---|---|---|---|---|---|---|---|---|---|---|---|---|---|
| | | Potatoes | | | Vegetables (excl. potatoes) | | | | Fruit | | | | |
| | | **Total** | Fresh | Processed | **Total** | Fresh green | Other fresh | Processed vegetables | **Total** | Fresh fruit | Processed fruit | Fruit juices | |
| **East** | **Chi$^2$** | **1.68** | **2.25*** | **1.44** | **2.18*** | **2.36*** | **2.79*** | **1.79** | **2.20*** | **2.33*** | **1.73** | **3.28*** | **2.08*** |
| Club&bargain store | T | 2.60 | 0.98 | 3.51 | 1.05 | 0.42 | 0.50 | 1.93 | 1.18 | 0.56 | 3.15 | 0.86 | 1.33 |
| | B | 2.66 | 1.02 | 3.62 | 1.04 | 0.47 | 0.49 | 1.89 | 1.25 | 0.57 | 3.45 | 0.79 | 1.36 |
| | D | 2.52 | 0.92 | 3.35 | 1.07 | 0.35 | 0.52 | 2.00 | 1.07 | 0.53 | 2.70 | 0.96 | 1.28 |
| Convenience | T | 5.98 | 6.25 | 5.82 | 6.53 | 6.45 | 7.34 | 5.99 | 6.45 | 7.13 | 5.29 | 4.78 | 6.42 |
| | B | 5.88 | 5.96 | 5.84 | 5.84 | 5.70 | 6.19 | 5.69 | 5.80 | 6.17 | 5.22 | 4.68 | 5.83 |
| | D | 6.11 | 6.73 | 5.79 | 7.59 | 7.62 | 9.12 | 6.44 | 7.45 | 8.60 | 5.38 | 4.91 | 7.32 |
| Discounter | T | 14.95 | 17.50 | 13.51 | 17.45 | 19.74 | 20.04 | 13.84 | 19.19 | 19.93 | 19.24 | 14.62 | 17.87 |
| | B | 15.28 | 17.46 | 14.00 | 18.04 | 20.61 | 20.80 | 14.05 | 19.50 | 20.41 | 19.31 | 14.26 | 18.30 |
| | D | 14.44 | 17.57 | 12.82 | 16.55 | 18.39 | 18.85 | 13.52 | 18.72 | 19.19 | 19.15 | 15.14 | 17.22 |
| Large store | T | 60.88 | 60.74 | 60.96 | 59.00 | 57.44 | 57.24 | 61.47 | 59.14 | 59.01 | 57.97 | 62.39 | 59.33 |
| | B | 63.23 | 63.76 | 62.92 | 61.85 | 60.08 | 60.37 | 64.28 | 62.07 | 61.95 | 60.21 | 66.71 | 62.14 |
| | D | 57.35 | 55.80 | 58.14 | 54.63 | 53.35 | 52.39 | 57.20 | 54.74 | 54.52 | 54.60 | 56.25 | 55.06 |
| Online | T | 15.40 | 14.31 | 16.00 | 15.74 | 15.63 | 14.57 | 16.70 | 13.87 | 13.20 | 14.25 | 17.11 | 14.86 |
| | B | 12.76 | 11.63 | 13.42 | 13.05 | 12.89 | 11.93 | 14.01 | 11.27 | 10.79 | 11.71 | 13.33 | 12.22 |
| | D | 19.37 | 18.71 | 19.71 | 19.88 | 19.86 | 18.67 | 20.77 | 17.78 | 16.87 | 18.07 | 22.49 | 18.87 |
| Other retailer | T | 0.19 | 0.21 | 0.19 | 0.21 | 0.31 | 0.31 | 0.07 | 0.16 | 0.17 | 0.10 | 0.23 | 0.19 |
| | B | 0.18 | 0.18 | 0.19 | 0.17 | 0.24 | 0.22 | 0.07 | 0.11 | 0.10 | 0.09 | 0.22 | 0.15 |
| | D | 0.21 | 0.25 | 0.19 | 0.29 | 0.42 | 0.45 | 0.07 | 0.24 | 0.28 | 0.11 | 0.24 | 0.25 |
| All retailers | T | 100.00 | 100.00 | 100.00 | 100.00 | 100.00 | 100.00 | 100.00 | 100.00 | 100.00 | 100.00 | 100.00 | 100.00 |
| | B | 100.00 | 100.00 | 100.00 | 100.00 | 100.00 | 100.00 | 100.00 | 100.00 | 100.00 | 100.00 | 100.00 | 100.00 |
| | D | 100.00 | 100.00 | 100.00 | 100.00 | 100.00 | 100.00 | 100.00 | 100.00 | 100.00 | 100.00 | 100.00 | 100.00 |

Continues

| | Period | Fruit and vegetable products (percentages) | | | | | | | | | | | Total |
|---|---|---|---|---|---|---|---|---|---|---|---|---|---|
| | | Potatoes | | | Vegetables (excl. potatoes) | | | | Fruit | | | | |
| | | **Total** | Fresh | Processed | **Total** | Fresh green | Other fresh | Processed vegetables | **Total** | Fresh fruit | Processed fruit | Fruit juices | |
| **London** | **Chi$^2$** | **1.74** | **2.14*** | **1.55** | **2.18*** | **2.46*** | **2.46*** | **1.90** | **2.17*** | **2.08*** | **2.99*** | **2.13*** | **2.10*** |
| Club&bargain store | T | 2.03 | 0.74 | 2.79 | 0.70 | 0.48 | 0.64 | 0.93 | 1.28 | 0.71 | 3.16 | 0.77 | 1.12 |
| | B | 2.01 | 0.70 | 2.80 | 0.71 | 0.51 | 0.62 | 0.94 | 1.30 | 0.66 | 3.44 | 0.61 | 1.13 |
| | D | 2.06 | 0.79 | 2.78 | 0.69 | 0.43 | 0.67 | 0.92 | 1.25 | 0.77 | 2.75 | 1.00 | 1.11 |
| Convenience | T | 7.82 | 8.38 | 7.48 | 9.77 | 8.97 | 9.95 | 10.26 | 11.52 | 13.03 | 9.24 | 8.03 | 10.33 |
| | B | 7.32 | 7.83 | 7.02 | 9.51 | 8.87 | 9.30 | 10.15 | 11.10 | 12.54 | 9.10 | 7.49 | 9.95 |
| | D | 8.56 | 9.25 | 8.17 | 10.16 | 9.13 | 10.91 | 10.42 | 12.13 | 13.73 | 9.45 | 8.80 | 10.88 |
| Discounter | T | 11.47 | 13.54 | 10.25 | 14.79 | 17.71 | 16.98 | 10.86 | 16.49 | 16.00 | 20.39 | 11.94 | 15.16 |
| | B | 11.74 | 13.95 | 10.40 | 15.36 | 18.53 | 17.81 | 11.07 | 16.83 | 16.42 | 20.35 | 12.53 | 15.58 |
| | D | 11.07 | 12.91 | 10.03 | 13.94 | 16.50 | 15.74 | 10.54 | 16.01 | 15.41 | 20.44 | 11.07 | 14.55 |
| Large store | T | 66.13 | 64.96 | 66.82 | 61.92 | 60.91 | 60.06 | 64.10 | 59.33 | 59.89 | 55.05 | 64.25 | 61.26 |
| | B | 68.67 | 67.65 | 69.29 | 64.39 | 63.09 | 62.69 | 66.65 | 62.02 | 62.49 | 58.12 | 66.68 | 63.85 |
| | D | 62.29 | 60.71 | 63.18 | 58.25 | 57.71 | 56.16 | 60.25 | 55.50 | 56.20 | 50.63 | 60.74 | 57.47 |
| Online | T | 12.22 | 12.24 | 12.21 | 12.57 | 11.67 | 12.14 | 13.62 | 11.01 | 10.14 | 11.88 | 13.78 | 11.82 |
| | B | 9.91 | 9.74 | 10.02 | 9.82 | 8.75 | 9.40 | 10.96 | 8.39 | 7.72 | 8.71 | 11.19 | 9.19 |
| | D | 15.71 | 16.16 | 15.45 | 16.68 | 15.93 | 16.20 | 17.64 | 14.74 | 13.59 | 16.47 | 17.51 | 15.67 |
| Other retailer | T | 0.34 | 0.15 | 0.45 | 0.24 | 0.26 | 0.24 | 0.23 | 0.36 | 0.22 | 0.28 | 1.24 | 0.31 |
| | B | 0.35 | 0.12 | 0.48 | 0.22 | 0.24 | 0.18 | 0.23 | 0.37 | 0.17 | 0.29 | 1.50 | 0.30 |
| | D | 0.32 | 0.18 | 0.39 | 0.28 | 0.30 | 0.32 | 0.23 | 0.36 | 0.29 | 0.26 | 0.87 | 0.32 |
| All retailers | T | 100.00 | 100.00 | 100.00 | 100.00 | 100.00 | 100.00 | 100.00 | 100.00 | 100.00 | 100.00 | 100.00 | 100.00 |
| | B | 100.00 | 100.00 | 100.00 | 100.00 | 100.00 | 100.00 | 100.00 | 100.00 | 100.00 | 100.00 | 100.00 | 100.00 |
| | D | 100.00 | 100.00 | 100.00 | 100.00 | 100.00 | 100.00 | 100.00 | 100.00 | 100.00 | 100.00 | 100.00 | 100.00 |

Continues

| | Period | Fruit and vegetable products (percentages) | | | | | | | | | | | Total |
|---|---|---|---|---|---|---|---|---|---|---|---|---|---|
| | | Potatoes | | | Vegetables (excl. potatoes) | | | | Fruit | | | | |
| | | **Total** | Fresh | Processed | **Total** | Fresh green | Other fresh | Processed vegetables | **Total** | Fresh fruit | Processed fruit | Fruit juices | |
| **Midlands** | **Chi$^2$** | **1.22** | **1.47** | **1.15** | **1.85** | **1.81** | **2.17*** | **1.78** | **1.83** | **1.94** | **1.64** | **1.88** | **1.71** |
| Club&bargain store | T | 3.44 | 0.96 | 4.82 | 1.18 | 0.25 | 0.34 | 2.44 | 1.50 | 0.68 | 4.29 | 0.92 | 1.67 |
| | B | 3.54 | 1.02 | 5.01 | 1.18 | 0.24 | 0.34 | 2.45 | 1.57 | 0.72 | 4.49 | 0.95 | 1.71 |
| | D | 3.29 | 0.87 | 4.54 | 1.17 | 0.27 | 0.35 | 2.42 | 1.40 | 0.62 | 4.00 | 0.89 | 1.60 |
| Convenience | T | 5.73 | 6.90 | 5.07 | 6.57 | 6.94 | 7.15 | 5.87 | 7.62 | 8.49 | 6.65 | 4.06 | 6.89 |
| | B | 5.50 | 6.72 | 4.79 | 6.24 | 6.79 | 6.62 | 5.59 | 7.46 | 8.29 | 6.69 | 3.62 | 6.65 |
| | D | 6.07 | 7.19 | 5.49 | 7.06 | 7.17 | 7.94 | 6.31 | 7.86 | 8.80 | 6.60 | 4.68 | 7.25 |
| Discounter | T | 19.26 | 23.83 | 16.72 | 23.41 | 26.71 | 26.49 | 18.83 | 24.91 | 24.95 | 26.19 | 21.98 | 23.41 |
| | B | 19.77 | 24.11 | 17.23 | 24.20 | 27.66 | 27.67 | 19.27 | 25.51 | 25.51 | 26.86 | 22.67 | 24.07 |
| | D | 18.50 | 23.37 | 15.98 | 22.19 | 25.27 | 24.73 | 18.14 | 24.01 | 24.10 | 25.21 | 21.01 | 22.41 |
| Large store | T | 58.65 | 56.60 | 59.78 | 55.67 | 53.62 | 53.47 | 58.72 | 54.21 | 54.46 | 51.14 | 59.03 | 55.51 |
| | B | 60.28 | 58.50 | 61.32 | 57.71 | 55.32 | 55.44 | 61.03 | 56.18 | 56.54 | 52.63 | 61.38 | 57.46 |
| | D | 56.16 | 53.48 | 57.55 | 52.55 | 51.03 | 50.50 | 55.15 | 51.27 | 51.31 | 48.98 | 55.73 | 52.55 |
| Online | T | 12.50 | 11.30 | 13.17 | 12.75 | 11.81 | 11.97 | 13.99 | 11.39 | 11.03 | 11.53 | 13.37 | 12.13 |
| | B | 10.48 | 9.31 | 11.17 | 10.34 | 9.54 | 9.56 | 11.48 | 9.00 | 8.68 | 9.14 | 10.78 | 9.79 |
| | D | 15.57 | 14.57 | 16.09 | 16.43 | 15.30 | 15.59 | 17.86 | 14.96 | 14.61 | 15.01 | 17.02 | 15.66 |
| Other retailer | T | 0.42 | 0.41 | 0.43 | 0.43 | 0.65 | 0.59 | 0.16 | 0.37 | 0.39 | 0.19 | 0.64 | 0.40 |
| | B | 0.43 | 0.34 | 0.49 | 0.32 | 0.46 | 0.38 | 0.18 | 0.28 | 0.26 | 0.19 | 0.61 | 0.32 |
| | D | 0.41 | 0.52 | 0.35 | 0.60 | 0.96 | 0.90 | 0.12 | 0.50 | 0.57 | 0.20 | 0.68 | 0.53 |
| All retailers | T | 100.00 | 100.00 | 100.00 | 100.00 | 100.00 | 100.00 | 100.00 | 100.00 | 100.00 | 100.00 | 100.00 | 100.00 |
| | B | 100.00 | 100.00 | 100.00 | 100.00 | 100.00 | 100.00 | 100.00 | 100.00 | 100.00 | 100.00 | 100.00 | 100.00 |
| | D | 100.00 | 100.00 | 100.00 | 100.00 | 100.00 | 100.00 | 100.00 | 100.00 | 100.00 | 100.00 | 100.00 | 100.00 |

Continues

| | Period | Fruit and vegetable products (percentages) | | | | | | | | | | | Total |
|---|---|---|---|---|---|---|---|---|---|---|---|---|---|
| | | Potatoes | | | Vegetables (excl. potatoes) | | | | Fruit | | | | |
| | | **Total** | Fresh | Processed | **Total** | Fresh green | Other fresh | Processed vegetables | **Total** | Fresh fruit | Processed fruit | Fruit juices | |
| **North** | **Chi²** | **1.67** | **2.52*** | **1.36** | **2.17*** | **2.65*** | **2.58*** | **1.68** | **2.42*** | **2.39*** | **2.75*** | **2.21*** | **2.16*** |
| Club&bargain store | T | 4.73 | 1.73 | 6.44 | 1.98 | 0.52 | 0.83 | 3.69 | 2.10 | 1.06 | 5.74 | 1.23 | 2.48 |
| | B | 4.81 | 1.78 | 6.59 | 2.00 | 0.52 | 0.86 | 3.72 | 2.15 | 1.07 | 5.98 | 1.30 | 2.52 |
| | D | 4.60 | 1.65 | 6.20 | 1.94 | 0.52 | 0.80 | 3.65 | 2.03 | 1.04 | 5.41 | 1.13 | 2.40 |
| Convenience | T | 5.95 | 7.51 | 5.06 | 6.51 | 6.89 | 6.97 | 5.95 | 7.17 | 7.96 | 6.15 | 4.43 | 6.69 |
| | B | 5.80 | 7.68 | 4.69 | 6.18 | 6.56 | 6.47 | 5.73 | 6.95 | 7.69 | 6.09 | 4.07 | 6.43 |
| | D | 6.18 | 7.24 | 5.60 | 7.02 | 7.39 | 7.72 | 6.30 | 7.50 | 8.37 | 6.23 | 4.94 | 7.09 |
| Discounter | T | 18.84 | 23.96 | 15.91 | 24.04 | 28.30 | 28.29 | 18.41 | 26.71 | 27.70 | 26.42 | 21.34 | 24.32 |
| | B | 19.11 | 24.37 | 16.03 | 25.14 | 29.83 | 29.83 | 18.99 | 27.59 | 28.68 | 27.22 | 21.62 | 25.17 |
| | D | 18.41 | 23.29 | 15.75 | 22.35 | 25.93 | 25.97 | 17.51 | 25.43 | 26.24 | 25.29 | 20.94 | 23.02 |
| Large store | T | 59.70 | 56.68 | 61.42 | 56.76 | 53.72 | 53.64 | 60.85 | 54.65 | 54.19 | 51.73 | 63.20 | 56.36 |
| | B | 61.73 | 58.72 | 63.50 | 58.48 | 55.27 | 55.25 | 62.71 | 56.57 | 56.01 | 53.68 | 65.72 | 58.22 |
| | D | 56.60 | 53.43 | 58.34 | 54.11 | 51.33 | 51.21 | 57.94 | 51.84 | 51.48 | 48.97 | 59.60 | 53.55 |
| Online | T | 10.46 | 9.74 | 10.87 | 10.36 | 10.04 | 9.88 | 10.90 | 9.10 | 8.82 | 9.75 | 9.51 | 9.85 |
| | B | 8.25 | 7.12 | 8.91 | 7.94 | 7.42 | 7.31 | 8.69 | 6.54 | 6.37 | 6.81 | 7.06 | 7.41 |
| | D | 13.83 | 13.92 | 13.78 | 14.10 | 14.09 | 13.74 | 14.36 | 12.86 | 12.48 | 13.91 | 13.01 | 13.53 |
| Other retailer | T | 0.33 | 0.37 | 0.30 | 0.34 | 0.53 | 0.39 | 0.19 | 0.26 | 0.27 | 0.21 | 0.29 | 0.30 |
| | B | 0.30 | 0.32 | 0.28 | 0.26 | 0.39 | 0.28 | 0.16 | 0.20 | 0.18 | 0.22 | 0.22 | 0.24 |
| | D | 0.37 | 0.46 | 0.33 | 0.47 | 0.75 | 0.55 | 0.23 | 0.35 | 0.40 | 0.20 | 0.39 | 0.40 |
| All retailers | T | 100.00 | 100.00 | 100.00 | 100.00 | 100.00 | 100.00 | 100.00 | 100.00 | 100.00 | 100.00 | 100.00 | 100.00 |
| | B | 100.00 | 100.00 | 100.00 | 100.00 | 100.00 | 100.00 | 100.00 | 100.00 | 100.00 | 100.00 | 100.00 | 100.00 |
| | D | 100.00 | 100.00 | 100.00 | 100.00 | 100.00 | 100.00 | 100.00 | 100.00 | 100.00 | 100.00 | 100.00 | 100.00 |

Continues

| | Period | Fruit and vegetable products (percentages) | | | | | | | | | | | Total |
|---|---|---|---|---|---|---|---|---|---|---|---|---|---|
| | | Potatoes | | | Vegetables (excl. potatoes) | | | | Fruit | | | | |
| | | **Total** | Fresh | Processed | **Total** | Fresh green | Other fresh | Processed vegetables | **Total** | Fresh fruit | Processed fruit | Fruit juices | |
| **South** | **Chi²** | **2.75*** | **3.65*** | **2.32*** | **3.04*** | **3.28*** | **3.74*** | **2.46*** | **2.87*** | **3.40*** | **2.08*** | **2.26*** | **2.91*** |
| Club&bargain store | T | 1.88 | 0.59 | 2.63 | 0.67 | 0.18 | 0.31 | 1.30 | 0.92 | 0.41 | 2.54 | 0.50 | 0.94 |
| | B | 1.96 | 0.59 | 2.79 | 0.67 | 0.18 | 0.32 | 1.30 | 0.95 | 0.41 | 2.72 | 0.45 | 0.97 |
| | D | 1.76 | 0.59 | 2.40 | 0.66 | 0.19 | 0.30 | 1.29 | 0.87 | 0.39 | 2.29 | 0.58 | 0.91 |
| Convenience | T | 6.11 | 6.94 | 5.63 | 6.78 | 6.91 | 7.36 | 6.24 | 7.51 | 8.27 | 6.45 | 5.19 | 7.00 |
| | B | 5.73 | 6.47 | 5.28 | 6.18 | 6.25 | 6.66 | 5.77 | 6.99 | 7.54 | 6.32 | 5.06 | 6.47 |
| | D | 6.68 | 7.69 | 6.13 | 7.70 | 7.93 | 8.43 | 6.98 | 8.26 | 9.37 | 6.64 | 5.38 | 7.81 |
| Discounter | T | 14.84 | 17.27 | 13.42 | 17.75 | 20.32 | 20.71 | 13.58 | 19.10 | 19.22 | 20.81 | 14.73 | 17.93 |
| | B | 15.43 | 17.86 | 13.96 | 18.56 | 21.25 | 22.02 | 13.93 | 19.79 | 19.97 | 21.62 | 14.81 | 18.66 |
| | D | 13.95 | 16.34 | 12.65 | 16.50 | 18.87 | 18.72 | 13.04 | 18.08 | 18.10 | 19.65 | 14.61 | 16.84 |
| Large store | T | 61.50 | 60.90 | 61.85 | 59.66 | 58.46 | 57.35 | 62.31 | 58.64 | 58.79 | 55.50 | 64.42 | 59.47 |
| | B | 64.54 | 64.36 | 64.65 | 62.77 | 61.53 | 60.36 | 65.52 | 61.67 | 62.13 | 57.60 | 67.55 | 62.54 |
| | D | 56.96 | 55.39 | 57.81 | 54.87 | 53.70 | 52.76 | 57.36 | 54.17 | 53.78 | 52.51 | 59.98 | 54.85 |
| Online | T | 15.39 | 14.07 | 16.17 | 14.93 | 13.84 | 14.00 | 16.44 | 13.63 | 13.12 | 14.51 | 14.84 | 14.43 |
| | B | 12.07 | 10.54 | 13.00 | 11.66 | 10.60 | 10.45 | 13.36 | 10.44 | 9.84 | 11.53 | 11.78 | 11.19 |
| | D | 20.36 | 19.69 | 20.73 | 19.96 | 18.88 | 19.38 | 21.19 | 18.34 | 18.06 | 18.72 | 19.19 | 19.30 |
| Other retailer | T | 0.28 | 0.23 | 0.30 | 0.22 | 0.29 | 0.27 | 0.13 | 0.21 | 0.19 | 0.19 | 0.31 | 0.22 |
| | B | 0.27 | 0.18 | 0.32 | 0.17 | 0.20 | 0.19 | 0.12 | 0.16 | 0.12 | 0.20 | 0.34 | 0.18 |
| | D | 0.29 | 0.31 | 0.28 | 0.30 | 0.42 | 0.41 | 0.13 | 0.27 | 0.30 | 0.18 | 0.27 | 0.29 |
| All retailers | T | 100.00 | 100.00 | 100.00 | 100.00 | 100.00 | 100.00 | 100.00 | 100.00 | 100.00 | 100.00 | 100.00 | 100.00 |
| | B | 100.00 | 100.00 | 100.00 | 100.00 | 100.00 | 100.00 | 100.00 | 100.00 | 100.00 | 100.00 | 100.00 | 100.00 |
| | D | 100.00 | 100.00 | 100.00 | 100.00 | 100.00 | 100.00 | 100.00 | 100.00 | 100.00 | 100.00 | 100.00 | 100.00 |

Continues

| | Period | Fruit and vegetable products (percentages) | | | | | | | | | | | Total |
|---|---|---|---|---|---|---|---|---|---|---|---|---|---|
| | | Potatoes | | | Vegetables (excl. potatoes) | | | | Fruit | | | | |
| | | **Total** | Fresh | Processed | **Total** | Fresh green | Other fresh | Processed vegetables | **Total** | Fresh fruit | Processed fruit | Fruit juices | |
| **Scotland** | **Chi$^2$** | **1.90** | **2.83*** | **1.60** | **2.44*** | **2.17*** | **2.80*** | **2.48*** | **2.64*** | **2.92*** | **2.47*** | **2.29*** | **2.41*** |
| Club&bargain store | T | 5.88 | 1.64 | 7.77 | 2.02 | 0.40 | 0.48 | 4.11 | 2.08 | 1.14 | 5.35 | 1.28 | 2.68 |
| | B | 5.74 | 1.67 | 7.66 | 2.00 | 0.40 | 0.48 | 4.05 | 1.87 | 0.97 | 5.13 | 1.08 | 2.55 |
| | D | 6.09 | 1.59 | 7.93 | 2.06 | 0.41 | 0.49 | 4.19 | 2.39 | 1.42 | 5.64 | 1.58 | 2.87 |
| Convenience | T | 7.53 | 9.11 | 6.82 | 7.27 | 6.96 | 7.83 | 7.07 | 7.81 | 8.91 | 5.87 | 5.28 | 7.55 |
| | B | 7.10 | 8.76 | 6.32 | 7.06 | 6.44 | 7.49 | 7.16 | 7.45 | 8.45 | 5.47 | 5.43 | 7.24 |
| | D | 8.16 | 9.69 | 7.53 | 7.58 | 7.76 | 8.36 | 6.93 | 8.34 | 9.61 | 6.44 | 5.06 | 8.01 |
| Discounter | T | 17.79 | 23.90 | 15.06 | 23.67 | 28.44 | 27.52 | 18.01 | 24.75 | 26.19 | 23.33 | 19.46 | 23.18 |
| | B | 17.85 | 23.81 | 15.03 | 24.35 | 29.10 | 28.67 | 18.40 | 25.63 | 27.45 | 23.57 | 19.29 | 23.85 |
| | D | 17.70 | 24.04 | 15.10 | 22.63 | 27.44 | 25.77 | 17.41 | 23.42 | 24.27 | 22.99 | 19.71 | 22.16 |
| Large store | T | 60.42 | 57.70 | 61.63 | 58.02 | 56.02 | 56.01 | 60.67 | 56.71 | 56.02 | 56.40 | 61.03 | 57.83 |
| | B | 62.99 | 60.47 | 64.18 | 60.10 | 58.09 | 57.79 | 62.94 | 58.92 | 57.89 | 59.29 | 63.98 | 60.05 |
| | D | 56.61 | 53.19 | 58.01 | 54.84 | 52.89 | 53.27 | 57.17 | 53.40 | 53.17 | 52.33 | 56.64 | 54.49 |
| Online | T | 8.13 | 7.32 | 8.49 | 8.82 | 7.98 | 7.89 | 9.99 | 8.48 | 7.57 | 8.93 | 12.60 | 8.55 |
| | B | 6.14 | 5.02 | 6.66 | 6.35 | 5.86 | 5.39 | 7.33 | 6.00 | 5.15 | 6.43 | 9.88 | 6.16 |
| | D | 11.08 | 11.08 | 11.09 | 12.59 | 11.19 | 11.71 | 14.08 | 12.19 | 11.27 | 12.45 | 16.64 | 12.16 |
| Other retailer | T | 0.26 | 0.32 | 0.23 | 0.20 | 0.20 | 0.26 | 0.16 | 0.18 | 0.17 | 0.12 | 0.36 | 0.20 |
| | B | 0.19 | 0.26 | 0.15 | 0.14 | 0.12 | 0.18 | 0.12 | 0.13 | 0.10 | 0.10 | 0.35 | 0.14 |
| | D | 0.36 | 0.41 | 0.34 | 0.30 | 0.32 | 0.39 | 0.23 | 0.26 | 0.28 | 0.15 | 0.37 | 0.29 |
| All retailers | T | 100.00 | 100.00 | 100.00 | 100.00 | 100.00 | 100.00 | 100.00 | 100.00 | 100.00 | 100.00 | 100.00 | 100.00 |
| | B | 100.00 | 100.00 | 100.00 | 100.00 | 100.00 | 100.00 | 100.00 | 100.00 | 100.00 | 100.00 | 100.00 | 100.00 |
| | D | 100.00 | 100.00 | 100.00 | 100.00 | 100.00 | 100.00 | 100.00 | 100.00 | 100.00 | 100.00 | 100.00 | 100.00 |

Continues

| | Period | Fruit and vegetable products (percentages) | | | | | | | | | | | Total |
|---|---|---|---|---|---|---|---|---|---|---|---|---|---|
| | | **Potatoes** | | | **Vegetables (excl. potatoes)** | | | | **Fruit** | | | | |
| | | **Total** | Fresh | Processed | **Total** | Fresh green | Other fresh | Processed vegetables | **Total** | Fresh fruit | Processed fruit | Fruit juices | |
| **Wales** | **Chi²** | **1.73** | **1.84** | **1.73** | **2.37*** | **2.34*** | **2.76*** | **2.27*** | **3.36*** | **3.49*** | **3.86*** | **2.90*** | **2.57*** |
| Club&bargain store | T | 4.80 | 1.82 | 6.52 | 1.85 | 0.29 | 0.67 | 3.65 | 1.70 | 0.72 | 4.78 | 1.30 | 2.31 |
| | B | 4.64 | 1.82 | 6.31 | 1.72 | 0.29 | 0.66 | 3.33 | 1.75 | 0.84 | 4.75 | 1.25 | 2.25 |
| | D | 5.04 | 1.84 | 6.82 | 2.06 | 0.29 | 0.68 | 4.14 | 1.62 | 0.54 | 4.83 | 1.37 | 2.40 |
| Convenience | T | 4.64 | 6.36 | 3.65 | 5.36 | 5.71 | 6.14 | 4.61 | 5.64 | 6.28 | 4.59 | 4.03 | 5.35 |
| | B | 4.44 | 6.31 | 3.34 | 5.20 | 5.41 | 5.87 | 4.60 | 5.45 | 6.05 | 4.51 | 3.77 | 5.16 |
| | D | 4.95 | 6.44 | 4.11 | 5.61 | 6.16 | 6.55 | 4.61 | 5.93 | 6.64 | 4.70 | 4.39 | 5.62 |
| Discounter | T | 18.06 | 21.94 | 15.82 | 23.30 | 27.83 | 27.88 | 17.29 | 25.67 | 25.97 | 28.39 | 18.80 | 23.33 |
| | B | 18.44 | 22.15 | 16.25 | 24.49 | 29.50 | 29.33 | 18.07 | 27.06 | 27.25 | 30.28 | 19.63 | 24.45 |
| | D | 17.48 | 21.61 | 15.17 | 21.46 | 25.31 | 25.65 | 16.09 | 23.63 | 24.04 | 25.66 | 17.67 | 21.65 |
| Large store | T | 61.32 | 60.09 | 62.03 | 57.27 | 54.71 | 54.12 | 61.06 | 55.86 | 56.19 | 50.93 | 63.37 | 57.41 |
| | B | 63.55 | 62.17 | 64.36 | 59.14 | 55.97 | 55.82 | 63.38 | 57.94 | 58.33 | 52.54 | 66.16 | 59.44 |
| | D | 57.92 | 56.81 | 58.54 | 54.41 | 52.81 | 51.50 | 57.46 | 52.77 | 52.95 | 48.59 | 59.54 | 54.36 |
| Online | T | 10.91 | 9.47 | 11.74 | 12.03 | 11.17 | 10.95 | 13.30 | 10.83 | 10.58 | 10.95 | 12.02 | 11.34 |
| | B | 8.66 | 7.31 | 9.46 | 9.35 | 8.70 | 8.20 | 10.53 | 7.57 | 7.38 | 7.43 | 8.95 | 8.51 |
| | D | 14.32 | 12.87 | 15.13 | 16.13 | 14.89 | 15.15 | 17.60 | 15.67 | 15.43 | 16.05 | 16.22 | 15.62 |
| Other retailer | T | 0.27 | 0.32 | 0.25 | 0.19 | 0.29 | 0.25 | 0.09 | 0.30 | 0.25 | 0.36 | 0.48 | 0.25 |
| | B | 0.26 | 0.24 | 0.28 | 0.10 | 0.13 | 0.10 | 0.09 | 0.24 | 0.16 | 0.49 | 0.25 | 0.19 |
| | D | 0.29 | 0.44 | 0.21 | 0.33 | 0.54 | 0.47 | 0.10 | 0.39 | 0.39 | 0.17 | 0.80 | 0.35 |
| All retailers | T | 100.00 | 100.00 | 100.00 | 100.00 | 100.00 | 100.00 | 100.00 | 100.00 | 100.00 | 100.00 | 100.00 | 100.00 |
| | B | 100.00 | 100.00 | 100.00 | 100.00 | 100.00 | 100.00 | 100.00 | 100.00 | 100.00 | 100.00 | 100.00 | 100.00 |
| | D | 100.00 | 100.00 | 100.00 | 100.00 | 100.00 | 100.00 | 100.00 | 100.00 | 100.00 | 100.00 | 100.00 | 100.00 |

Source: Own elaboration based on Kantar Worldpanel data.
Note: T stands for the entire period, B for before COVID-19 lockdown and D during the lockdown period. $Chi^2$ is the test for the null hypothesis that the distributions before and during COVID-19 are the same. *' indicates that the null hypothesis is rejected at 5 per cent significance.
